# Proceedings of the World Society of Cardiovascular and Thoracic Surgeons 29th Annual Congress, Sofia, Bulgaria, 2019: Sofia, Bulgaria. 6-8 September 2019

**DOI:** 10.1186/s13019-019-0971-2

**Published:** 2019-09-06

## O1 Renal failure incidence post sequential bilateral lung transplantation

### Florentina Popescu, Jorge Mascaro

#### Cardiopulmonary Transplantation, Queen Elizabeth Hospital, Birmingham, UK

##### **Correspondence:** Florentina Popescu

Acute renal dysfunction post lung transplant is one of the morbidities encountered that will impact the outcome and costs of both hospitalisation and community care. This is a retrospective study analysing 51 cases of sequential bilateral lung transplant over the last 3 years. As primary variables we analysed pre and post-operative eGFR, ischaemic time, bypass time, intra/post-operative blood products, inotropic support peri - operatively, CVVH required, haemodialysis on discharge, duration of postoperative ventilation, length of stay in intensive care unit, return to theatre. Secondary variables analysed were bilateral lung transplant, and comorbidities. There were 29 patients who developed post-operative renal impairment, (57%) out of which 18 patients required CVVH within the first week of ITU stay. Out of these patients, there were 7 patients who were discharge to the ward on haemodialysis. Out of the 29 patients who developed post-operative renal impairment (eGFR<60), only 4 patient had pre-operative renal impairment (eGFR <60). Out of the 7 patients discharged to the ward on dialysis, 6 patients required post-operative inotropes, only one pre-operative inotropes and 6 patients required intra-operative blood products. 18 patients requiring CVVH post-transplant had a median bypass time of 300 mins and an ischaemic time of 350 mins. Out of the 18 CVVH patients, 17 patients required blood transfusion intra-operatively and 15 patients post-operatively. Out of the CVVH patients 15 patients had a prolonged ventilation (>1 day). A prolonged ischaemic time, as well as a prolonged operative time, mainly due to bilateral lung transplantation, as well as usage of blood products intra-operatively and inotropic support post operatively seems to be associated with a high incidence of renal impairment post sequential bilateral lung transplantation.

## O3 Examination of the Methods of Surgery in Treatment of Pulmonary Aspergilloma

### Reza Bagheri^1^, Seyed ziaollah Haghi^1^, Reza Afghani^2^, Shahrzad Lari^1^, Vahab Azmounfar^3^, Saeed Hakimian^4^, Mohammad Firoozabadi^4^, Elham Lotfian^5^

#### ^1^Lung disease Research Center, Mashhad University of Medical Sciences, Mashhad, Iran; ^2^Thoracic Surgeon, department of general surgery, 5th of Azar Hospital, Faculty of Medicine, Golestan University of Medical Sciences, Gorgan, Iran; ^3^Medical student, Student Research Committee, Faculty of medicine, Mashhad university of medical science, Mashhad, Iran; ^4^Medical student, Mashhad University of Medical Science, Mashhad, Iran; ^5^biologist, research expert, Lung disease Research Center, Emam Reza hospital, Mashhad university of medical science, Mashhad, Iran

##### **Correspondence:** Reza Bagheri

**Background**

Although surgery has had a high level of morbidity and mortality, it is the more preferable treatment for this disease; an issue that is still controversial.

Materials and Methods:

In a descriptive study, 30 patients with pulmonary Aspergilloma who were treated via surgery, were examined retrospectively. In this study, the patients were examined based on their age, sex, pre-surgery clinical symptoms, the injured part, the method of surgery and the post-surgery problems.

**Results**

These patients include 21 men and 9 women with the average age of 48.13. The most common symptom was hemoptysis (90%), the cough with sputum and drug-resistant pneumonia were also seen. The most common problematic parts include LUL and RUL. The surgery was carried out on 21 patients by lobectomy and 9 patients (30 %) by segmentectomy. After the surgery, residual space, wound infection, Bronchopleural fistula were seen respectively in 5 cases (16.7%), 3 cases (10%), and 2 cases (6.7%). The mortality rate was 1 person.

**Conclusions**

In comparison of the method of surgery in treatment of pulmonary Aspergilloma (lobectomy and segmentectomy) and the sex, clinical symptoms, post-surgery side effects and mortality, no meaningful relationship was seen. As a result, these methods of surgery are successful.

**Key words**

Pulmonary, Aspergilloma, surgery method

## O6 Endoscopic transthoracic sympathectomy is a useful option for severe intractable angina and catecholaminergic polymorphic ventricular tachycardia

### Mitsuhiro Kamiyoshihara, Hitoshi Igai, Natsumi Matsuura, Ryohei Yoshikawa, Fumi Ohsawa, Tomohiro Yazawa

#### Department of General Thoracic Surgery, Japanese Red Cross Maebashi Hospital, Maebashi, Gunma, Japan

##### **Correspondence:** Mitsuhiro Kamiyoshihara

**Background**

Intractable angina and catecholaminergic polymorphic ventricular tachycardia is a rare but severe cardiac disease that carries a risk of sudden cardiac death. The purpose of this study was to assess the feasibility and usefulness of endoscopic transthoracic sympathectomy (ETS) for this disease.

**Materials and methods**

From June 2010 to September 2019, six patients who were unable to tolerate, or who were refractory to, medical therapy underwent ETS. Under general anesthesia, the pleural cavity was entered through two 5-mm incisions in the subaxillary area. The thoracic sympathetic chain was identified, and the T2 to T4 sympathetic chain was cauterized and resected using a bipolar energy device on the right and left sides, respectively.

**Results**

The mean patient age was 51 years, and the male to female ratio was 5:6. The diseases included intractable angina in five patients and catecholaminergic polymorphic ventricular tachycardia in one patient. Of these, three patients had a previous history of cardiac arrest. The mean operative time was 81 ± 10 min. Blood loss was minimal. The median postoperative stay was 14.8 days and the median follow-up period was 47.2 months. There were no major complications in the intra- and postoperative courses. Postoperatively, the frequency of cardiac attacks decreased from 3.1 times per day to 0.7 every few months. No syncopal events have occurred to date. The mean medicine dosage decreased from 8.1 drugs to 3.2.

**Conclusions**

TS is a safe and effective treatment option for patients with intractable angina and arrhythmia refractory to medical management.

## O7 The novel use of oral antibiotic monotherapy in prosthetic valve endocarditis caused by Finegoldia magna: a case study

### Siobhan Chien^1^, David Gorman^1^, Charilaos-Panagiotis Koutsogiannidis^1^, Vipin Zamvar^1^, Ramanish Ravishankar^2^

#### ^1^Department of Cardiothoracic Surgery, Royal Infirmary of Edinburgh, Edinburgh, UK; ^2^University of Edinburgh, Edinburgh, UK

##### **Correspondence:** Siobhan Chien

**Background**

Finegoldia magna, a Gram-positive anaerobic coccus, is part of the human normal microbiota as a commensal of mucocutaneous surfaces. However, it remains an uncommon pathogen in infective endocarditis, with only eight clinical cases previously reported in the literature. Currently, infective endocarditis is routinely treated with prolonged intravenous antibiotic therapy. However, recent research has found that switching patients to oral antibiotics is non-inferior to prolonged parenteral antibiotic treatment, challenging the current guidelines for the treatment of infective endocarditis.

**Case presentation**

This case report focuses on a 52-year-old gentleman, who presented with initially culture-negative infective endocarditis following bioprosthetic aortic valve replacement. Blood cultures later grew Finegoldia magna. Following initial intravenous antibiotic therapy and surgical replacement of the prosthetic aortic valve, the patient was successfully switched to oral antibiotic monotherapy, an unusual strategy in the treatment of infective endocarditis inspired by the recent publication of the POET trial. He made excellent progress on an eight-week course of oral antibiotics and was successfully discharged from surgical follow-up.

**Conclusions**

This case is the 9th reported case of Finegoldia magna infective endocarditis in the literature. Our case also raises the possibility of a more patient-friendly and cost-effective means of providing long-term antibiotic therapy in suitable patients with prosthetic valve endocarditis, and suggests that the principles highlighted in the POET trial can also be applicable to post-operative patients after cardiac surgery.

## O8 Comparison of short-term outcomes for cabg surgery between the royal infirmary edinburgh and the literature

### Bosire Oroko, Vipin Zamvar

#### University of Edinburgh, Edinburgh, UK

##### **Correspondence:** Bosire Oroko

**Introduction**

The efficacy of CABG surgery is a widely researched area. Studies commonly report and compare various outcomes of these procedures. The aim of this project is to compare the CABG surgery outcomes reported in the literature with outcomes at the Royal Infirmary Edinburgh.

**Methods**

A literature search for relevant RCTs and observational studies was conducted. Demographic and short-term outcome data were collected and totalled to determine a combined ‘literature’ value for each individual demographic/outcome. Outcomes at the RIE were obtained from a database of prospectively collected data on all patients undergoing cardiac surgery. Data were compared using Chi-squared testing.

**Results**

65 studies were selected. The literature group had 1,668,490 CABG patients and the RIE group had 1,338. 25 outcomes were compared between these groups. There were significantly higher rates of post-operative AF and chest infections in the RIE group (RR 1.43, 8.35 respectively). The rest of complication rates at the RIE were lower or the difference was statistically insignificant.

**Conclusions**

Large differences in demographics and outcome reporting between studies in the literature and the RIE made comparison difficult. Caution should be taken giving meaning to these results. Outcomes should be more clearly and consistently defined in future research so that outcomes can be compared across studies in the literature.

## O9 Myocardial revascularization using the two internal mammary arteries: advantages and disadvantages

### Housni Larguat, Aksas Sana, Boudiaf Elhadj

#### EHS Dr m.a Maouche, algiers, Algeria

##### **Correspondence:** Housni Larguat

**Introduction**

Myocardial revascularization represents a major chapter in cardiac surgery, and is a common practice in our department at EHS Dr. maaouche mohand amokrane, the use of both internal mammary arteries is the gold standard (1) with a permeability exceeding twenty years however, only one internal mammary artery associated with veins is commonly used (2) due to lack of technicality and fear of complications (2), (3). From January 2018 to October 2018 we operated 70 patients referred to us for myocardial revascularization, 56 benefited from revascularization in all arterial. The purpose of our study is to determine the advantages and disadvantages of using both mammary arteries.

**Patient and Method**

From January 2018 to the end of October 2018, 70 consecutive patients underwent myocardial revascularization including 58 men and 12 women, the average age is 60 years with extremities of 39 to 86 years. 53.06% of patients were diabetic, 69.38% had hypertension, 59% were obese, 77% of our patients had a coronary event in their history (43% NSTEMI, 34% STEMI), 14 patients were left ventricular dysfunction with an ejection fraction of less than 40%. 7.46% benefited from double bypass, 61.19% from triple bypass, 20.89% from a quadruple bypass and 10.44% from a fivefold bypass. Both breast arteries were used in 85.71%. The use of the mammary Y (right breast cut and anastomosed to the left breast which is pedicled) was the reference fitting in the majority of the cases ie 80%, whereas the use of the saphenous vein was reserved only in case of defective or deficient breast graft. The average duration of extracorporeal circulation is 72.9 min (33-154), the average duration of clamping is 52.68min (22-108), the average duration of intubation is 13.44 hours (5-120h).

**Result**

The operative mortality was 5.7% (04 patients), 03 patients were resumed for bleeding, 01 patient (1.42%) had a postoperative myocardial infarction, 01 patient (1.42%) had a stroke, 02 patients (2.85%) had a mediastinitis, one of which required a resumption in the operating room with the realization of an epiplooplasty the postoperative course of this patient was very satisfactory, 05 patients (7% ) had poorly perfused cutaneous ischemia requiring twice-daily bandaging to achieve total healing after 02 to 03 months.

**Conclusion**

The all arterial coronary bypass graft using the two left and right internal mammary arteries is the best choice for myocardial revascularization, with a permeability reaching twenty years and remains superior to the other grafts, mediastinitis and cutaneous ischemia remain the main complications but their incidence is low. The use of the mammary Y allows a complete revascularization providing a revascularization for each territory reached, the technique requires a learning curve but remains perfectly reproducible.

**References**

(1) Anelechi C. Anyanwu, MD, David H. Adams, MD, Total Arterial Revascularization for Coronary Artery Bypass A Gold Standard Searching for Evidence and Application JOURNAL OF THE AMERICAN COLLEGE OF CARDIOLOGY VOL72, NO.12, 2018.

(2) Randomized comparison of single versus bilateral internal thoracic artery grafts in 3102 CABG patients: Major cardiovascular outcomes at ten years of follow up David P Taggart, Arterial Revascularization Trial (ART) ESC 2018.

(3) Randomized trial to compare bilateral vs. single internal mammary coronary artery bypass grafting: 1-year results of the Arterial Revascularisation Trial (ART) David P. Taggart Douglas G. Altman Alastair M. Gray Belinda Lees Fiona Nugara Ly-Mee Yu Helen Campbell Marcus Flather on behalf of the ART Investigators European Heart Journal, Volume 31, Issue 20, 1 October 2010, Pages 2470–2481,

## O11 Narrow ostium of aorta during isolated aortic aortic valve replactment: choice of correction

### Konstantin Vakulenko, Volodymyr Popov, Olexandr Bolshak

#### National Institute of Cardio-Vascular Surgery named after Amosov, Kiev, Ukraine

##### Correspondence: Volodymyr Popov

**PURPOSE** of this investigation is to research possibilities of original method of posterior aortoplasty (PA) during aortic valve replacement (AVR) in patents (pts) with narrow ostium of aorta (NOA). To determine significance of patient-prosthesis mismatch (PPM).

**Materials and methods**

In analyzed group were included 825 pts with isolated aortic stenoses with NOA wich were consecutive operated in Institute from 01.01. 2010 till 01.01.2017. There were 464 (56,2%) males and 361 (43,8%) females in average age 57,5+8,4 yy. 315 (38,2%) pts belonged to III NYHA class and 510 (61,8%) - to IV. Body surface area (BSA) was 1,95+0,08 m². Average diameter of fibrotic annulus was 2,04+0,03 cm. Peak gradient on aortic valve was:103,7 + 15,3 (87-145) mm Hg. Operations were performed by following methods: group A – AVR (model 23) + original method of reconstruction by PA (n=89); group B - AVR with model 21 mm (n=379); group C- AVR with model 19 mm (n=357). The following patches were used: Vascutek’s (n=57), autopericardial (n = 11), bovine biocor SJM (n = 21). Only bileaflet prosthesis were used. Оperations were performed in conditions of moderate hypothermia (27-32° C) and ante-retrograde crystalloid cardioplegia (mainly Custadiol).

**Results**

The hospital mortality were: group A - 4,5%; group B - 2,7; group C - 4,9% (p< 0,05). At discharge of index of effective orifice area (IEOA) (cm²/m²) and peak gradient on aortic prosthesis (mm Hg)(PGAP) were marked for: group A - 0,95 + 0,03 and 22,3 + 2,7; group B = 0,88 + 0,03 and 26,3 + 3,8; group C - 0,82 + 0,04 and 35,3 + 5,2 (p< 0,05).

At the remote period (average was 7,3± 0,9 yy) 753 (92,6%) pts were followed–up at 7 years after operation. In group A (n = 83) survival rate 83.4% and stability of good results 63.5% were observed. In group B (n = 343) survival rate 78.3% and stability of good results 53.4% were marked. In group C (n = 327) survival rate 49.3% and stability of good results 33.3% were occured.

At remote period IEOA (cm²/m²) and PGAP (mm Hg) were marked for: group A - 0,92 + 0,03 and 21,3 + 2,3; group B - 0,84 + 0,04 and 29,3+ 3,9; group C - 0,78 + 0,04 and 42,3+ 4,7 (p< 0,05).

**Conclusion**

Reconstruction of NOA during AVR by proposed original method of posterior aortoplasty is effective intervention especially at remote period in group A. PPM was marked significantely in group C.

## O12 Surgical treatment of poststenotic aneurysms of ascending aorta

### Volodymyr Popov, Ivan Kravchenko, Vitaly Kravchenko, Vasily Lazorishinetz

#### National Institute of Cardio-Vascular Surgery named after Amosov, Kiev, Ukraine

##### **Correspondence:** Volodymyr Popov

**Aim**

To research possibilities of surgical treatment of poststenotic aneurysms of ascending aorta (PAAA) by different methods.

**Methods**

During 2000-2018 yy 701 patients (pts) with aortic stenoses (AS) and PAAA were consecutively operated in Institute. The average age of pts was 61,2±8,9 (18 –71) yy. Males 411 (58,6%), females 290 (41,4%). At all group 25 (3,6 %) pts were in II NYHA class, 323 (46,1%) pts were in III NYHA class and 353 (50,3%) pts in IV. The following operations were performed: aortic valve replacement (AVR) + wrapping tape operation (WTO) of AA – 257 pts (group A), AVR + Robischek’s operation – 307 pts (group B), replacement of AA by vascular graft (n=137): Benthal’s (n= 128) and Wheat’s (n= 9) operations (group C). In all cases in group A after AVR nylon tape (diameter 1 cm) was wrapped on AA from the basement of noncoronary cusp by 7-9 tours and fixated between them in proximal and distal part of AA and resection of AA in incision’s area. All operations were performed with CPB, moderate hypothermia (27 - 32 C), combined retro-antegrade crystalloid cardioplegia(mainly Custadiol).

**Results**

Hospital mortality were 0,4% in group A, 1,1% in group B and 2,9% in group C (p < 0.05). Cross-clamping time (min) were: (group A) - 73,5±8,4, (group B) - 102,6±14,7 and (group C) - 134,9±19,8 (p < 0.05).

During remote period (average 11,4±1,6 yy) we followed-up 649 pts. Actuarial survival at 10 years after operation was occured in group A – 89,5% (n=249), in group B – 86,3% (n=283), and group C – 76,3% (n= 117) (p<0.05).

Echo examination of diameter of AA for group A (cm): preoperative (PRE) 4,5±0,5, postoperative (POST) (6–7 days) 3,6±0,4, remote period (RP) 3,7±0,4; for group B: preoperative 4,8±0,5, postoperative – 3,9±0,4, remote period 4,1±0,3 and for group C: preoperative 5,6±0,8, postoperative – 3,3±0,4, remote period 3,4±0,2. Reoperations (AA’s graft replacement) were absents in group A and C. Benthall operation was occurred in group B after 3 years of Robischek’s reconstruction in 2 cases.

**Conclusion**

On the basis of our experience we recommend the expedient method of wrapping tape operation for moderate forms of AAA (diameter of AA till 5,5 cm) during AVR. Reconstruction of AA for PAAA by WTO is safe, chiper and prevents AAA at the remote period.

## O13 Isolated mitral valve replacement without usage of donor blood

### Volodymyr Popov, Bogdan Gumenyuk

#### National Institute of Cardio-Vascular Surgery named after Amosov, Kiev, Ukraine

##### **Correspondence:** Volodymyr Popov

**Aim**

To study features of mitral valve replacement (MVR) without use of donor blood or its components.

**Material**

489 patients (pts) with isolated mitral valve disease were operated (MVR) in National Institute of CVS in period from 01.01.2000 till 01.01.2014. All operations were carried out without using of donor blood or its components during treatment, and while cardiopulmonary bypass (CPB) haemoconcentrated columns, or a sell-savers were not applied. There were 214 males and 275 females. Mean age was 51,8± 6,2 yy. To IV class by NYHA classification belonged 312 (63,8 %) pts, 178 (30,3 %) pts to III class and 29 (5,9 %) pts to II class. At 372 (76,1%) pts 468,7 ± 51,4 ml of blood on citrate was taken before the cross-clamping. Diuresis was stimulated on the beginning of operation by 80 mg furosemide and 100 ml mannite. Patient was completed by 6 % refortane in a doze 250-300 ml before starting CPB, and the water balance on this stage was in the ranges + 284,4±39,6 ml. Retrograde autological priming (RAP) was occurred in 81 (16,6%) pts in doses 588,5±71,2ml. All operations were carried out in conditions of moderated hypothermia and retrograde crystaloid cardioplegia in a combination to external heart cooling. By the time the end of perfusion the water balance did not exceed ranges of 700-900,0 ml. After a stop of CPB its contents by maximum was returned into patient, including full evacuation of the CPB’s reservoir. Average time of cross-clamping was 53,4±7,2 min, bloodless - 242,2±34,8 ml.

**Results**

Hospital mortality (HM) among 489 pts was 1,0 % (5 pts died). Duration of stay on artificial lung ventilation was 5,5 ± 0,8 hours, in intensive care unit - 59,5 ± 7,5 hours, average time of the postoperative period was 9,1 ± 0,7 days. At discharge moderate anemia (reduction of hemoglobin from 145 ± 9,8 g/L to 105 ± 14,3 g/L from initial) was marked.

**Conclusion**

We recommend our method of MVR without use of donor blood and its components. Exceptions for it are significant anemia (hemoglobin less than 120) , weight of patients less than 65 kg, arterial hypertension with hypertrophic left ventricle.

## O14 Pre-and postcondition of coronary artery and myocardium for isolated mitral valve replacement

### Volodymyr Popov, Alex Gurtovenko, Bogdan Gumenyuk

#### National Institute of Cardio-Vascular Surgery named after Amosov, Kiev, Ukraine

##### **Correspondence:** Volodymyr Popov

**Objective**

To present analysis of pharmacological supporting of conditions of coronary artery and myocardium (pre and postcondition) during isolated mitral valve replacement (MVR).

**Materials and methods**

During 2015-2016 y 203 patients (pts) with isolated pathology of mitral valve disease were operated by MVR in department of surgery of acquired valve diseases. There were 91 (44,8%) males, 112 (55,2%) females. Average age was 64,4±8,7 yy. NYHA class in all group were followings: III class – 77 (37,9%), IV class – 126 (62,1%) pts. Concomitant procedures: TV’s plasty (n=9), LA’s plasty (n=41), Maze (n=29). Pharmacological supporting of conditions of coronary artery and myocardium (PSCCAM) was performed by applying during 20 minutes of 100 ml solution with following drugs: papaverine 40mg + verapamil 5mg + ATP 50,0 mg.

All pts were divided at 3 groups: group A - PSCCAM was applied before starting of CPB - 37 pts; group B - PSCCAM was applied before starting of CPB and the same dose was used after declamping of aorta - 39 pts; group C - only MVR 127 pts.

Systemic hypothermia 32-34 C, cardiopulmonary bypass, retrograde cardioplegic solution (Custadiol) (in dose 20 ml/kg) were occured in all pts. Average time of improvement of cardioplegia solution was 21,2±3,9 minutes. Average cross-clamping time (min) were: 69,3±8,1 and reperfusion time - 29,1±4,5. Absence of using blood product in 48,5%.

**Results**

There weren’t any pts of hospital mortality. Average doses of dobutamin (1,8±0,6 mcrg/min/kg) were marked (hours) for: group A - 24,8±7,2; group B - 19,1±5,7; group C - 39,5±5,4 (p <0,05). Average level of MB KFK (U/L) at 2-td postoperative day were occured for: group A - 56,3±7,2; group B - 53,4±6,8; group C - 61,1±9,3 (p<0,05). Duration of stay on artificial lung ventilation (hours) were: group A - 7,1±0,9; group B - 6,9±0,7; group C - 7,4±0,5 (p>0,05). Average time of staying in intensive care unit (hours) were: group A - 47,2±5,6, group B - 43,3±6,5, group C - 48,7±5,7 (p <0,05).

**Conclusion**

Both variances of pharmacologic supporting of conditions of coronary artery and myocardium (group A, B) had improved myocardial protection compare with group C (p<0,05).

## O15 Surgical treatment of myxomas of the heart

### R.Vitovskiy, Volodymyr Popov, V. Isaienko, V.Lazorishinetz

#### National Institute of Cardio-Vascular Surgery named after Amosov, Kiev, Ukraine

##### **Correspondence:** Volodymyr Popov

**Purpose** – to determine the possibilities of surgical treatment of myxomas (MH) of the heart.

**Material and methods**

In Institute for period from 1.01.1969 to 1.01.2018 916 patients (pts) with the verified primary tumors of heart were operated . The MH were exposed at 818 (89,3%) patients, from them in 718 (87,8%) cases – MH the left atrium (LA). The myxomas of right atrium (RA) were determined in 73 (9,5%) supervisions, MH in LV and RV – for 8 (1,0%) cases accordingly. Multicentral growth of MH with a defeat two or three chambers of heart was discovered at 11 (1,3%) patients. Average age of pts with MH were 47,5 ± 3,4 years(3- 78). By III and to the IV functional classes of NYHA classification were taken - 297 (36,3%) and 69 (8,4%) patients accordingly. At macroscopic research MH it was certain that tumors it was been: villiferous – in 472 (57,7%) cases and compact new formations of ovoid or rounded form in 346 (42,3%) accordingly. All operations were performed with CPB, moderate hypothermia (32-34 C), antegrade crystalloid cardioplegia .

**Results**

At surgical treatment MH hospital mortality was 4,8% (39 cases). In the last 17 years 455 operations were executed without fatal outcomes. Reasons of fatal outcomes it was been: neurological complications – at 16 (46,2%) pts; material embolism - in 12 (30,8%) pts, infarct of myocardium – in 3 (7,7%) pts; septic complication – in 1 (2,6%) pts.

In a follow-up period the results of surgical treatment MH were studied for 698 patients (89,6% written) in terms from 6 months to 47 years (on the average 19,5±4,2 years). Survivability in terms to 20 years was 79,7%. In a follow-up period in I NYHA class were 547 (78,4%) patients, in II l class – 103 (14,8%). Relapses MH discovered for 16 (2,1%) patients in period from 2 to 12 years (on the average 3,5±0,4 years) after operation. All were reoperated.

**Conclusion**

The adopted tactics ensure the effectiveness of surgical treatment of MH, confirmed by given good follow-up results.

## O17 The role of la’s diameter in recovery of sinus rhythm during valve correction

### Volodymyr Popov, Victoria Roy, Katerina Pukas, Alina Topchiy

#### National Institute of Cardio-Vascular Surgery named after Amosov, Kiev, Ukraine

##### **Correspondence:** Volodymyr Popov

Purpose of investigation is to research possibilities of intraoperative renewal of sinus rhythm during mitral valve replacement (MVR).

**Methods and materials**

In analyzed group of 391 patients are included with isolated mitral valve disease who were operated in Institute from 01.01.2009 to 01.01.2019. There were 170 (43,5%) males and 221 (56,5%). females. Average age of patients was 60,3 + 9,5 yy. 131 (33,5%) patients belonged to III NYHA class, 260 (66,9%) patients - to IV class. Valve’s correction: mitral (mainly MVR) (n = 337), aortic (n = 43), mitral-aortic (n = 11) were performed in all patients. Average being of permanent form of atrial fibrillation was 2,9 + 0,4 yy. Operations of left Maze – III-box (n=120) and Maze – IV- box (n=271) were performed in all cases by radio-frequency method + sew-tecnique. Ligation (n=27) and resection of LA’s auricle (n=364) in both groups were occured. Reduction of left atrium’s (LA) dilatation was occurred in 273 (69,8%) pts by 3 methods: paraannular plasty of LA (62 pts), triangular plasty of LA (original method) (68 pts) and arch plasty of LA (original method) (143 pts). Оperations were done in conditions of moderate hypothermia (32-34° C), retrograde crystalloid cardioplegia (Custadiol). Time of cross-clamping was 75,1 + 10,4 min. There were no complications attributed with method of operation.

**Results**

Among 391 operated patients 7 patient died on a hospital stage (hospital mortality-1,8%) because of pneumonia (n=2), brain damage (n=1), MOF (n=4) . Inotropic support (dobutamine) was in within 2,45 + 0,32 mcgr/min/kg during first 49,5 + 5,2 hours. Duration of staying on artificial lung ventilation was 7,3 + 0,8 hours and in intensive care unit 63,4+ 7,2 hours. Sinus rhythm renewed at discharging was registered in 82,4 %. In LA’s plasty group (n=273) diameter of LA were decreased at postoperative period: 63,7 + 2,3 (before), 51,3 + 1,4 (after), 52,3 + 1,6 (remote period). Dinamic of LA’s diameter in alternative group (n=118): 63,7 + 2,5 (before), 61,3 + 1,3 (after), 70,3 + 0,8 (remote period). Renewal of sinus rhythm in group pts with LA’s plasty was higher, than in alternative group at the hospital period: 86,8% (n=237/273) and 72,0% (n= 85/118) (р<0,01). 371 (97,1%) pts were followed during 7 years after operation. In group with renewed sinus rhythm it was retained 7 year: 95,2% and alternative group - 46,1% (р<0,01).

**Conclusion**

Valve’s correction with concomitant operation Maze-III, IV allows successfully renew sinus rhythm on a hospital stage and stabilize it well during remote period after operation. Element of left atrium’s plasty with reduction of diameter of LA less than 50 mm is important factor for sinus rhythm renewal and stability.

## O18 The remote results after isolated aortic valve replacement

### Konstantin Vakulenko, Volodymyr Popov

#### National Institute of Cardio-Vascular Surgery named after Amosov, Kiev, Ukraine

##### **Correspondence:** Volodymyr Popov

**Aim**

The purpose of the research is to analyzed the characteristics of a remote period after isolated aortic valve replacement (AVR).

**Material and methods**

In the analyzed group included 754 patients discharged after isolated AVR at the Institute for the period 2006-2007. This represented 96.7% of discharged on hospital stage. There were 403 (53.4%) men, women 351(46.6%). The age of patients ranged from 20 to 72 years (mean 53,7 ±9.4 years). By NYHA classification there were followed-up: II class 55 (7.3%) patients, III class 303 (40.2%) patients and IV class 396 (52.5%) patients. Atrial fibrillation was observed in 21 (2.8%) patients. Only mechanical prostheses were implanted: (Saint Jude, On-X, Carbomedics, ATS). Concomitant CABG was observed in 103 (13.7%) patients.

**Results**

Average followed-up at remote period 9.4 ± 0.7 yy At 10 years we had observed: survival rate was 71.3%, stability of good results was occurred 57.3%, freedom from thrombembolic complications were observed in 95.3%, freedom from reoperations was observed in 97.1%. Reoperations were occured: thomboses (panus) of aortic prostheses (n=2), prosthetic endocardytis (n=3). Atrial fibrillation was marked in 37 (4.9%) patients. A-V blocade was occured in 22 (2.1%) patients. The main risk factors for remote period: IV functional class, atrial fibrillation, left atriomegaly (diameter of atrium 6.0 cm or more), ejection fraction less than 0.4, high pulmonary hypertension (PSP > 70 mm.Hg), left ventriculomegaly (ESVI > 95 ml/m.q), progressive ischemic heart disease.

**Conclusion**

At the remote period good results of the operation by mechanical aortic prostheses was observed in the most cases. Operation should be better perform in II-III functional class, with sinus rhythm and good myocardial contractility.

## O19 Reconstruction of left part of the heart for mitral valve diseases

### Volodymyr Popov, Oleksandr Bolshak , Viktoriya Roy, Vasiliy Lazorishinetz

#### National Institute of Cardio-Vascular Surgery named after Amosov, Kiev, Ukraine

##### **Correspondence:** Volodymyr Popov

**Objective**

To determined possibillities of correction of the left parts of the heart by preservation of MV’s apparatus and reduction of LA during MVR.

**Methods**

During 2007 - 2016 yy. 91 adult patients (pts) were operated with mitral valve diseaes (MVD) and giant LA (diameter 60 mm and more) at Institute (group A). Average age was 49,2± 5,6 yy. 63 (68,9%) pts were in IY NYHA class and 28 (31,1%) in III class. There were used bileaflet prostheses (n = 56) monodisc type with orientation of the large margin to the posterior leaflet (n = 35). LA’s plasty was performed by Kawazoe’s method. Preservation of posterior leaflet (all pts) and translocatioon of anterior leaflet’s papillary muscles (n=45) was performed together with MVR. Concomitant procedure were occured on aortic valve (n =21) and tricuspid vave (n = 9). Combined ante-retrograde crystaloid cardioplegia and moderate hypothermia (30 -32 C) were used. Cross-clamping time of aorta was 93,4 ± 11,2 minutes. Control group (mitral inssuffiency) - only MVR without preservation of MV (n= 41) (group B).

**Results**

There were 2 deaths at the hospital period (hospital mortality (HM) - 2,2%) (group A). The reasons of deaths were heart failure (1), brain damage (1). There aren’t any episods of bleeding, thrombembolic events or prostheses’s failure at the hospital and remote period. At the remote period (average was 5,3± 1,4 yy) 83 pts were followed –up. Sinus rhytm was preserved at 43 (47,2%) pts and there weren’t any deaths or unsatisfactive results. Data of echo for group A - end-systolic volume index (ESVI) (ml/m.sq.) - preoperative 62,8 ± 6,4, postoperative (6 -11 dd) - 54,4 ± 7,4 and at the remote period 49,6 ± 5,2 and diameter of LA (mm) preoperative - 62,4 ± 4,2, postoperative - 46,4 ± 4,2, remote period - 45,8 ± 3,6. No hospital mortality in group B. Data of echo for group B - ESVI - preoperative 81,8 ± 9,2, postoperative (6 -11 dd) - 74,6 ± 8,4 and remote period 72,4 ± 8,2 and diameter of LA (mm) preoperative - 72,4 ± 4,2, postoperative - 67,4 ± 6,2, remote period – 70,1 ± 8,4. In group B (n=41) there were episods of thrombembolic events (n=3), heart failure (n=4), prostheses’s failure (0). Sinus rhytm wasn’t marked in any pts and there were two deaths, unsatisfactive results (n=4 - progressive heart failure).

**Conclusion**

Reconstruction of the left part of the heart for MVD by preservation of MV and LA’s plasty during MVR was allowing to improve indixes of LV’s and LA’s morphometry, contractility during early and at the remote period comparing with group B. There weren’t any specific complications at the postoperative period in group A.

## O21 Sulcus ventriculo-pulmonalis cordis and its significance in adhesive pericarditis

### Iovcho Topalov^1^, Georgi Marinov^2^, Dragomir Dardanov^3^

#### ^1^University Hospital “Heart and Brain”, Pleven, Bulgaria; ^2^Medical University – Varna, Varna, Bulgaria; ^3^University Hospital “Alexandrovska”, Medical University – Sofia, Sofia, Bulgaria

##### **Correspondence:** Dragomir Dardanov

Sulcus ventriculo-pulmonalis cordis is described as a normal structure of human heart. This groove is difficult to be distinguished on hearts from healthy individuals. It is situated on the border of muscle parts of conus pulmonalis to the base of arteria pulmonalis. Its filling with adipose tissue covered by epicardium makes it hardly visible. The importance of the sulcus is noticeable in right ventricle hypertrophy which develops in patients with adhesive pericarditis. In this situation the sulcus is filled with fibrous tissue and a triangular stenosing band is formed. This band obstruct the blood flow from right ventricle to pulmonary artery and right heart failure develops. Authors of the study propose an original operative method for removing of the described fibrous band. The knowledge about sulcus ventriculo-pilmonalis cordis and its stenosing band in patients with adhesive pericarditis is key moment in pericardialysis.

## O22 Impact on patient outcomes before and after implementation of CABG clinical pathway

### Kee Soon Chong, Paneer Selvam, Jeswant Dillon

#### Cardiothoracic Surgery Department, Institut Jantung Negara, Kuala Lumpur, Malaysia

##### **Correspondence:** Kee Soon Chong

**Background**

Isolated CABG is the most commonly performed adult cardiac surgery. CABG pathway with clearly defined and reproducible goals would improve clinical outcome. There are 4 specific goals. 1. Surgery within 72 hours after admission. 2. Extubation within 12 hours. 3. Transfer out of ICU within 48 hours. 4. Discharge within postoperative day 6.

**Method**

Retrospective analysis of patient outcomes after implementation of CABG pathway in 2017, compared with matched patients in 2016. Inclusion criteria: elective isolated CABG with good LVEF ≥50%. Exclusion criteria: renal impairment (male Cr >130mmol/L, female Cr >103mmol/L), history of stroke and history of COAD/asthma. The data of 4 specific goals, post-operative complications, mortality and cost per patient were analysed and compared.

**Results**

1422 cases of isolated CABG performed 2017, 368 patients (25.9%) were enrolled into CABG pathway. In 2016, 1549 cases of isolated CABG performed, 347 patients (22.4%) were eligible. Demographics, pre-morbidities and Euroscore II were similar in both groups. The intubation period was shorter after pathway implementation (12.7±5.3 hours vs 16.6± 12.1 hours, p<0.001). Similarly, shorter ICU stay (31.4±17.7 hours vs 39.7±30 hours, p=0.006) and earlier hospital discharge (6.8±1.9 days vs 7.7±3.5 days, p<0.001) were observed. Respiratory complication rate was seen lower (1.6% vs 4.6%). Clinical outcomes in these highly selected good risk patients were excellent. 30 days mortality was 0% in 2017 and 0.27% in 2016, in comparison to benchmark mean Euroscore II (1.74±0.85% and 1.73±0.64%). Better clinical performance in 2017 translated into significant decrease in total cost of CABG/patient (-4.6%, p=0.006).

**Conclusions**

Reduction in cost and hospital stay with improve clinical outcome were clearly evidenced from our findings. However, elements of CABG pathway may differ in each institution depending on the clinical support and personnel. As good as the benefits have been shown, for a successful and sustainable implementation of CABG pathway, constant audit and improvement is absolutely paramount.

## O23 Non-toxicity of intrapleural sericin administration in the nervous system of rats

### Alkin Yazicioglu^1^, Tuba Sahinoglu^2^, Serkan Uysal^3^, Mehmet Sahin^1^, Funda Demirag^4^, Erdal Yekeler^1^

#### ^1^Department of Thoracic Surgery, Ankara City Hospital, Ankara, Turkey; ^2^Department of Thoracic Surgery, Konya Numune Hospital, Konya, Turkey; ^3^Department of Thoracic Surgery, Hacettepe University Medical Faculty, Ankara, Turkey; ^4^Department of Pathology, Ataturk Chest Disease and Thoracic Surgery Training and Research Hospital, Ankara, Turkey

##### **Correspondence:** Alkin Yazicioglu

**Background**

Sericin is a natural macromolecular adhesive protein that is derived from the cocoons of silkworm. There has been no study to date in literature evaluating the potential for neurotoxicity associated with sericin pleurodesis. The present study evaluates the potential of intrapleural sericin administration to cause neurotoxicity.

**Methods**

Adult, male, Wistar-Albino rats aged 12 weeks, weighing 211-256 gr (n=22) were divided randomly into two groups, each comprising 11 rats (Ethics Committee: Kobay AS, 292/2018). A left thoracotomy was performed following intramuscular anesthesia. The A group was administrated sericin 30 mg and B group constituted sham thoracotomy group. The rats were fed ad-libitum, all were sacrificed on day 13. The brain and cerebellum were excised en-bloc; T9-L3 segment was excised and a sampling was made from sciatic nerve.

**Results**

The brain specimens were evaluated for the presence of subarachnoid hemorrhage, congestion, eosinophilic material, edema, necrosis, gliosis, conspicuous capillary endothelium, and pyknotic nuclei. The cerebellar specimens were evaluated for the presence of shrinkage in the Purkinje cells, capillary hemorrhage, gliosis, necrosis, edema and congestion. The medulla spinalis was evaluated for hemorrhage in the central canal, as well as for ependymal cell degeneration, edema and necrosis. The sciatic nerve was evaluated for congestion, edema and inflammation.

A subarachnoid hemorrhage was observed in the brain specimens of four rats (36.4%) in the control group and six rats (54.5%) in the sericin group (p=0.416). A capillary hemorrhage in the cerebellum was observed in six rats (54.5%) in the control group and in one rat (9.1%) in the sericin group (p<0,05; p=0,024). A hemorrhage was observed in the central canal in three rats (27.3%) in the control group, whereas no hemorrhage was observed in the sericin group (p=0.082). Congestion in the fibers of the sciatic nerve was observed in five rats (45.5%) in the control group and in seven rats (63.6%) in the sericin group (p=0.416). The observation of capillary hemorrhage in the cerebellar specimens was significantly more common in the control group.

**Conclusions**

The administration of intrapleural sericin does not cause neurotoxicity in rats, and can be safely used in pleurodesis procedures.

## O24 Mediastinitis in patients post open heart surgery – prevention and predicting factors

### Hristo Stoev, Zaprin Vazhev, Todor Gonovski, Asen Ivanov, Konstantin Dimitrov

#### St. George university hospital– plovdiv, Department of cardiac surgery, Medical university-plovdiv, Department of cardiac and vascular surgery

##### **Correspondence:** Hristo Stoev

**Background**

Deep wound infections are a serious complication after open heart surgery and are directly related to patient survival both in the short and long term. Despite prevention, their appearance continues to be significant - 0.5% - 6.8% and the associated in-hospital mortality ranges from 7% to 35%.

**Materials and methods**

The report presents 7- year experience of the Cardiac surgery department with prophylaxis, diagnosis and treatment of patients who develop deep wound infections after open heart surgery covering the period of January 2011 to January 2018. For this period 4563 patient with different types of cardiac pathology we operated, 94 (2.06%) of them developed deep wound infection. Patients are divided into groups depending on the type of surgery – CABG, valve correction, aortic surgery or combined procedures. The study is retrospective, and the clinical data used is from the hospital records.

**Results**

Sex ratio is 1,5:1 – males/females. The mean age for both sexes is 67.5 years. In hospital mortality was evaluated - 22.34% (21 patients). In 100 of the patients PCT and CRP were tested during the first postoperative day. 15 of them PCT was more 2 ng/ml and 9 (60%) patients developed deep wound infection. In all these patients, bacterial agent was isolated from wound and vacuum-assisted therapy was used. In 2 cases we used titanium plates for sternal reconstruction along with mobilization of omentum majus.

**Conclusions**

Open heart surgery with cardiopulmonary by-pass leads to a risk of developing mediastinitis. Improved surgical techniques and research on specific biomarkers could reduce the development of this life-threatening complications.

**Keywords**

Open heart surgery, extracorporeal circulation, deep wound infection, biomarkers

## O25 Redo mitral valve replacement on fibrillating heart – is the outcome better?

### Dimitar Petkov, Jordan Makedonski, Angel Doichev, Gencho Nachev

#### Department of cardiosurgery, University Hospital Sv.Ekaterina, Sofia, Bulgaria

##### **Correspondence:** Dimitar Petkov

**Introduction**

Nowadays needs for redo cardiac surgery is more common. Number of patients requiring reoperation for significant mitral regurgitation is increasing. The aim of present work is to review our experience with redo cases for mitral valve replacement on fibrillating heart.

**Patients and methods**

Between 2011 and may 2019 a total number of 111 patients, 55 female and 56 male, with average age 62 years (from 42 to 75), underwent redo mitral surgery in our institution. Those were divided in two groups. Group 1 – patients operated on fibrillation heart without aortic cross-clamping (32 patients, average age 61 years, 11 female and 21 males) and Group 2 – patients operated with aortic cross-clamping and cardioplegic arrest (79 patients, average age 62 years, 44 female and 35 male). In all patients mitral valve replacement was performed. In 52 patients at least one more major cardiac procedure was also performed during the same surgery.

**Results**

In hospital mortality in group 1 was 3 patients (9,3%) while in group 2 it was 14 patients (17,7%). The CPB time was shorter in group 1 (average 95 min) compared to group 2 (108 min). Complications rate were as follows: heart failure – 4 patients (12,5%) in group 1 versus 18 patients (22.78%) in group 2; acute renal failure – 2 patient (6,25%) in group 1 versus 19 patients (24%) in group 2; sepsis – 2 patients (6,25%) in group 1 versus 9 patients (11,4%) in group 2; CNS complications – 1 patients (3,12%) in group 1 versus 4 patients (5%) in group 2.

**Conclusions**

Our experience shows that redo cases for mitral valve replacement on fibrillating heart have better outcome. The mortality and morbidity are reduces. We believe that the technique is easier and straightforward.

## O26 Benefits of rapid deployment aortic valve replacement with a mini upper sternotomy

### Charilaos-Panagiotis Koutsogiannidis, Saumya Maheshwari, Vipin Zamvar, Vincenzo Giordano, Kelvin Lim, Renzo Pessotto

#### Cardiothoracic Surgery Department, Royal Infirmary of Edinburgh, Edinburgh, UK

##### **Correspondence:** Charilaos-Panagiotis Koutsogiannidis

**Background**

In 2014 rapid deployment aortic valves were introduced into our clinical practice. These valves are more expensive but facilitate minimally invasive approaches to aortic valve replacement.

Purpose of this study is to investigate the benefits of minimally invasive-rapid deployment aortic valve replacement in various subgroups.

**Materials and methods**

In three years, 714 patients underwent isolated aortic valve replacement in our centre. Based on the implanted aortic valve prosthesis and the surgical approach, we divided them into two groups: 61 patients (8.5%) who received a rapid deployment aortic valve replacement with a J-shaped mini upper sternotomy (MIRDAVR group) and the rest (653, 91.5%) who had either full sternotomy (using conventional or rapid deployment valve) or minimally invasive approach with conventional valve (CONVAVR group).

We retrospectively analysed data from out cardiac surgery database. Pre-operative characteristics and demographics, intra-operative times and post-operative outcomes were recorded and analyzed. Outcomes were also calculated in two different subgroups: octogenarians and high-risk patients (logEuroscore>10%).

**Results**

Pre-operative characteristics and demographic details showed that there were more women (62.3 vs. 43.6%) and more elderly patients (78.3 vs. 68.9 years old) in MIRDAVR group. In post-operative outcomes MIRDAVR group had significantly shorter mean aortic cross clamp (47.3 vs. 80.1 min) and cardiopulmonary bypass periods (63.7 vs. 104 min) than CONVAVR group. MIRDAVR had less intensive care unit stay and ventilation times compared to CONVAVR group, however, this did not reach statistical significance.

In octogenarians, MIRDAVR group had not only significantly shorter intraoperative periods but also significantly less intensive care unit stay (30.9 vs. 65.6 hours) and ventilation times (11.7 vs. 21.2 hours) compared to CONVAVR group. In high risk patients (logEuroscore>10%), MIRDAVR group had also significantly shorter cardiopulmonary bypass periods but not significantly less aortic cross clamp time. Also, the differences between the two groups in intensive care unit stay and ventilation times were no more significant.

**Conclusions**

In our cohort, aortic valve replacement with a rapid deployment prosthetic valve through a minimally invasive surgical approach provided shorter intra-operative periods and better outcomes. Furthermore, this correlation becomes much stronger in octogenarians but weaker in high-risk patients.

## O27 Liver dysfunction predicts poor outcomes in adult extracorporeal membrane oxygenation support

### Nikola Dobrilovic^1,2^, Robert March^2^, Omar Lateef^2^, Karl Karlson^1^, Jaishankar Raman^2^

#### ^1^Division of Cardiac Surgery, Boston University, Boston, Massachusetts, USA; ^2^Department of Cardiovascular and Thoracic Surgery, Rush University, Chicago, Illinois, USA

**Introduction**

Extracorporeal membrane oxygenation (ECMO) support can provide critically ill patients an opportunity to survive otherwise lethal illness. With roughly half of patients surviving ECMO, it remains unclear how and when to apply this labor-intensive, costly resource. Clear predictors of outcomes have yet to be defined. We examine the role of liver dysfunction in adult ECMO patients as a potential prognostic marker.

**Methods**

This study reports a nine-year, retrospective, single institution experience examining all adult patients for whom ECMO support was utilized. Trends in liver function were examined (total bilirubin, ALT, and AST). Bilirubin was considered to be elevated when > 15 as per ELSO reporting standards. ALT and AST were considered elevated when > 20 times the upper limit of normal. This study was approved by our institution's IRB committee (160662707-IRB01).

**Results**

All adult patients cannulated for ECMO during the study period (n=153) were included without exception. Mean age was 50 (range 19-82) years, 87 male, 66 female. Forty-four percent (68/153) of adult patients met at least one criterion for liver dysfunction. Mean duration of ECMO support was 17 days. Overall hospital mortality was 56% (86/153).

Hospital mortality in patients with elevated bilirubin was 88.5% (vs. 49% when not elevated; Odds Ratio 8.0, 95% CI 2.3-28.1 p=0.001), and similarly 84.1% for elevated ALT (vs. 45.0%; OR 6.5, 95% CI 2.7-15.8, p<0.001), 82.8% for elevated AST (vs. 38.9%; OR 7.5, 95% CI 3.4-16.7, p<0.001), 83.1% when either ALT or ALT were elevated (vs. 39.2%; OR 10.3, 95% CI 4.6-23.5, p<0.001), and 84.1% when both ALT and AST were elevated (vs. 45.0%; OR 6.5, 95% CI 2.7-15.8, p<0.001).

**Conclusion**

Increases in total bilirubin, ALT, and AST levels all correlated with early mortality despite good cardio-pulmonary support. Profound liver dysfunction in patients supported with ECMO is a poor prognostic sign associated with exceedingly high mortality. It is expected that liver function studies will play a significant role in patient selection criteria regarding 1) initiation of ECMO, 2) as a criterion for termination of ECMO, and 3) possibly as a trigger for use of liver support devices.

## O28 Application of super uniport VATS without muscle and nervous injured

### Zhangfan Mao, Gaoli Liu, Dong Ping, Haifang Hu, Shaowen Zhang, Shize Pan

#### Thoracic surgery department of Renmin Hospital, Wuhan University, Wuhan, Hubei, China

##### **Correspondence:** Zhangfan Mao

**Background**

Uniport VATS is popular all over the world. Most thoracic surgery can be finished by uniport VATS no matter simple pulmonary bulla resection or double sleeve lobectomy. But the incision of uniport VATS is made by electrotome with the injury of the chest wall muscles and intercostal nerves, This is the main cause of postoperative pain. We made the 15mm uniport incision by blunt separation without using of electrotome to perform bulla resection, wedge resection, and simple lobectomy.

**Method**

The incision was made by scalpel and located in the fourth or fifth intercostal space of the axillary midline. The uniport was about 10mm in size. Subcutaneous tissue and muscles of the chest wall and intercostal were bluntly separated with the vascular clamp. After inserting the wound protector, the diameter of the hole is about 15-20 mm. 10 mm thoracoscopy and 5 mm double-joint oval forceps for good thoracic exploration. When the 12mm endo stapler was inserted for resection, the 5mm thoracoscopy was used instead. We named it “super uniport”.

**Result**

A total of 21 operations were performed by super uniport VATS including 15 bulla resection, 5 wedge resection, and 1 simple lobectomy. The procedure and operation time were similar with uniport VATS in bulla resection, wedge resection. But it was more difficult and more time cost in the right inferior lobectomy than usual VATS. Pain scores were obviously improved in super uniport VATS (P<0.05).

**Conclusion**

Incision size and pain score of super uniport VATS were improved because of no damage in muscles and intercostal nerves. Although it will make the operation more difficult, it can be used in simple thoracoscopic surgery.

## O29 Combined surgical treatment of a patient with multiple aorto-arterial aneurysmal disease with synchronic sfa obliteration, not suitable for evar

### Elena Domuschieva^1^, Borislav Denchev^1^, Valentin Govedarski^2^, Todor Zahariev^1,2^

#### ^1^Virgin Mary University Hospital, Department of Vascular Surgery, Burgas, Bulgaria; ^2^Saint Ekaterina University Hospital, Department of Vascular Surgery, Sofia, Bulgaria

##### **Correspondence:** Elena Domuschieva

**Introduction**

Aortic aneurysms represent a complex and difficult surgical problem to solve. Around 75% of them affect the abdominal aortic segment. During their progression in time, as well as their predisposition to rupture, it is of paramount importance to adequately diagnose the asymptomatic types, as well as to choose the correct surgical strategy of conduct. In rarer cases (around 10%-15%) we are talking about multiple aorto-arterial aneurysmal disease, with multilevel localization of more than one arterial segment. Despite the rapid evolution of endovascular surgical treatment of the latter, in some cases the possibilities of stent-graft implantation are limited and even impossible. This is valid especially in the case of multilevel aorto-arterial aneurysmal disease, combined with obliterative distal arterial lesions.

**Clinical case**

We are presenting the clinical case of a 71-year-old patient with an asymptomatic abdominal aortic aneurysm (AAA). CT-angiography demonstrated a large AAA (8 cm/12 cm), bilateral aneurysms of the common iliac arteries (CIA), accompanied by kinking and elongation, as well as bilateral aneurysms of the common femoral arteries (CFA). A distal occlusion of the right SFA was also concluded. The AAA was with infrarenal localization and a proximal aortic neck, that was too high with further kinking, as well as an 80° angulation in the ventral direction. The overall workup findings did not allow us to proceed with an EVAR. We moved on with a combined surgical treatment that has further resection of AAA, disconnection of the iliac aneurysms, resection of the bilateral CFA aneurysms with interposition of a bifurcated 16 × 8 mm vascular graft. Effective TEA of the right SFA with a follow-up re-thrombosis and subsequent revision by a distal femoro-popliteal supragenicular bypass prolongation.

**Result**

The patient was discharged on the 10th post-operative day without any surgical or angiological complications.

**Discussion**

The combined surgical treatment in the case of multiple aorto-arterial aneurysmal disease with distal arterial occlusions is an important prognostic factor as a whole. In these type of circumstances, the presence of contraindications for endovascular stent grafting is greatly defined by the correct preoperative clinical and CT-angiographic interpretation. Performing combined arterial operations of the abdominal aorta in two and more distal arterial segments allows for radical problem solving in connection with the treatment of multiple aorto-arterial aneurysmal disease as well as distal obliterative lesions of the SFA.

**Key words**

Multiple aorto-arterial aneurysmal disease, SFA, surgical strategy, EVAR.

The patient provided written consent to the publication of clinical or personal details.

## O30 Evaluation of aortic valve sparing techniques in ascending aortic aneurysm and dissection

### Mostafa Tolba, Mostafa Elhelali, Salah El-Din Khalaf, Mohamed Fouda, Mohamed Saleh

#### Cardiac surgery department, Nouvel Hopital Civil, Strasbourg university, France and Cardiothorathic surgery department , Mansoura university , Egypt

##### **Correspondence:** Mostafa Tolba

**Background**

The aortic valve-sparing operation captured the interest of surgeons and cardiologists due to the known limitations of prosthetic valves. On the one hand, mechanical prostheses require lifelong anticoagulation and present associated thromboembolic and hemorrhagic morbidity. On the other hand, biological prostheses are likely to require future reoperations.

**Objectives**

Aim of this study was to evaluate the two main surgical technique of valve sparing (Yacoub procedure) and (David procedure) incuding the survival rate and the occurrence of valve related morbidity that included aortic insufficiency grade 3 or greater, aortic valve-related reoperation and aortic valve endocarditis after both techniques. Patients and methods: Our study conducted at cardiothoracic surgery department at university hospitals of Strasbourg and our department at Mansoura university in the period between January 2015 November 2016 and we did follow-up of 6 months , 1 year and 2 years for all patients included in the study, after taking approval from the medical ethical committee at both Universities and a written consent from all patients included at our study . Results: mid term results of both main techniques of valve sparing root replacement (David and Yacoub) are comparable as regard the survival rate and the occurrence of valve related morbidity that included aortic insufficiency grade 3 or greater, aortic valve-related reoperation and aortic valve endocarditis after both techniques , even the occurrence of postoperative complication after both technique is quit similar with no statistically significant difference between both techniques , apart from longer bypass time that is associated with Yacoub technique which may affect the choice of the surgeon to prefer doing valve sparing root replacement with David procedure to shorten bypass time. Conclusions: Valve-sparing aortic root replacement is an attractive option for treating aortic root pathology both main techniques of valve sparing root replacement (David and Yacoub) are comparable as regard the survival rate and the occurrence of valve related morbidity.

**Key words**

Aortic root aneurysm repair , aortic dissection repair , Aortic valve sparing root repalcement procedure.

## O31 Outcome predictors for surgical AF ablation concomitant to mitral valve surgery

### Simon Pecha, Johannes Petersen, Christian Meyer, Hermann Reichenspurner

#### University Heart and Vascular Center, Hamburg, Germany

##### **Correspondence:** Simon Pecha

**Objectives**

Concomitant surgical ablation is an established procedure, recommended in guidelines for patients with atrial fibrillation (AF) undergoing cardiac surgery. AF is very common among patients with mitral valve disease. We therefore analyzed predictors of rhythm outcome in a large patient collective receiving mitral valve surgery and concomitant ablation.

**Methods**

Between 2003 and 2016, 419 patients with persistent (n= 266, 63.5%) or paroxysmal (n= 153, 36.5 %) AF underwent surgical AF ablation concomitant to mitral valve surgery. 209 (49.8%) patients received isolated MVR, while 210 (50.1%) patients received combined mitral valve procedures. The lesions were either limited to a pulmonary vein isolation (n= 39, 9.3%), a complete left atrial lesion set 256 (61.1%), or biatrial lesions (n= 124, 29.6%). Follow-up rhythm evaluations were based on either 24 h-Holter ECG or event recorder interrogation at 3, 6, and 12 months postoperatively.

**Results**

Mean patients age was 66.1+/-14.6 years, 238 (56.8%) were male. There were no major ablation-related complications. Survival rate after 1 year follow-up was 93%. After 1-year follow up, freedom from AF was 65.2%, showing significantly better results in patients with paroxysmal AF compared to those with persistent AF (75.6%vs.58.3%, p=0.0014). Logistic regression analysis confirmed smaller left atrial diameter (p=0.023), and paroxysmal AF (0.0011) as statistically significant predictors for freedom from AF. Neither energy source, nor additional surgical procedure significantly influenced rhythm results. Regarding only patients with persistent AF, those receiving a biatrial lesion set showed a trend towards higher rates of freedom from AF, but without statistically significant differences (biatrial 66.9% vs..left-atrial 55.3%, p=0.067).

**Conclusion**

Surgical AF ablation, concomitant to mitral valve surgery is a safe and effective procedure. Statistically significant predictors for freedom from AF after 12 months were preoperative paroxysmal AF and smaller left atrial diameter.

## O32 Mid and long term outcome and factors influencing survival after off-pump coronary artery bypass grafting

### Permyos Ruengsakulrach^1^, Kosin Thupvong^1^, Vitoon Pitiguagool^1^, Piyapan Pamornsing^1^, Siriwasan Akanitthapichat^1^, Jamorn Udomkusonsri^1^, Mantana Chudtong^2,3^, Pairote Satiracoo^2,3^

#### ^1^Division of Cardiovascular Surgery, Bangkok Heart Hospital, Bangkok Hospital Group, Bangkok, Thailand; ^2^Department of Mathematics, Faculty of Science, Mahidol University, Bangkok, Thailand; ^3^Centre of Excellence in Mathematics, CHE, Bangkok, Thailand

##### **Correspondence:** Permyos Ruengsakulrach

**Background**

To evaluate the mid and long term outcome and factors influencing survival after Off-pump Coronary Artery Bypass Grafting (OPCAB).

**Materials and methods Results**

All 1827 consecutive patients who had undergone OPCAB between January 2001 and December 2018 were studied. The study was approved by Bangkok Hospital Institution Review Board, approval number 2019-23. Demographics, clinical baseline, and surgical details were collected. The mean age was 63.1±10.5 years and 79.6% was male. The incidence of common risk factors was as follows: diabetes 49%, hypertension 74.1%, cerebrovascular accident 6.6%, peripheral vascular disease (PVD) 2.6%, heart failure 21.8%, cardiogenic shock 5.6%, recent myocardial infarction 14.6%, and previous cardiac surgery 2.8%. The mean left ventricular ejection fraction (LVEF) was 54.8±15.2%. Significant left main disease (LM ≥50% stenosis) was found in 34.6%. The mean last preoperative serum creatinine was 1.2±0.9 mg/dl. The average number of grafts was 4.4±1.3. The left internal thoracic artery (LITA), right internal thoracic artery, radial artery, gastroepiploic artery and saphenous vein graft (SVG) were used as a graft in 94.9, 19.4, 56.4, 14.7 and 52.5% of the patients, respectively. Perioperative intra-aortic balloon pump (IABP) was used in 16.5%. Conversion to On-Pump CABG was 1%. One hundred and sixty-eight (9.2%) patients died during follow-up. The 1-yr, 3-yr, 5-yr and 10-yr survival rate were 95.7, 94.3, 92.2 and 86.2%, respectively with the mean follow-time of 5.2 years. In Cox regression analysis, covariate selection (11 out of 24 covariates) to identify the best-fit model was performed based on Akaike Information Criterion. Age (P<0.001), diabetes (P=0.013), PVD (P=0.009), serum creatinine (P <0.001), left main disease (P=0.04), LVEF (P <0.001), LITA (P=0.04), SVG (P=0.01) and used IABP (P<0.001) were significantly influencing the survival rate.

**Conclusions**

The 5-yr and 10-yr survival rates after OPCAB were 92.2 and 86.2%, which was comparable with On-Pump CABG, previously reported. Aging, Diabetes, PVD, LM disease, poor LVEF, high preoperative serum creatinine, the use of perioperative IABP and SVG were associated with poor survival rate while LITA grafting was associated with longer survival rate after OPCAB.

## O33 Chemical pleurodesis using a tetracycline for management of post-operative recurred spontaneous pneumothorax

### HongKyu Lee, WeonYong Lee, HoHyun Ko, ByungMo Gu

#### Department of thoracic and cardiovascular surgery, Hallym University Sacred Heart Hospital, Anyang-si, Gyeonggi-do, Republic of Korea

##### **Correspondence:** ByungMo Gu

**Purpose**

Chemical pleurodesis has been used as a non-surgical treatment to reduce the recurrence rate in patients with persistent air leakage or frequent recurrence in spontaneous pneumothorax(SP). Although there is no clear guideline for the treatment of recurred pneumothorax after surgical treatment, chemical pleurodesis with tetracycline has been used as one of the important treatment methods. The purpose of this study is to determine the efficacy of chemical pleurodesis using a tetracycline for management of postoperative recurred spontaneous pneumothorax.

**Material and Methods**

Retrospectively, we reviewed patients who underwent video-assisted thoracic surgery (VATS) as a therapy for SP at the single center institute from January 2010 to December 2016. Among these, patients who had postoperative ipsilateral recurrence were included in this study. Patients underwent pleural drainage with chemical pleurodesis were compared with patients pleural drainage alone.

**Result**

Total of 932 patients who underwent VATS for SP were reviewed and postoperative ipsilateral recurrence occurred in 67 (7.1%) patients. Median age was 17 years (13 to 38). Mean follow up time was 60 month (14 to 88), and median time of recurrence was 10.5 month (1 to 45 months). Treatment modalities for postoperative recurred pneumothorax were observation (n=12), pleural drainage alone (n=16), pleural drainage with chemical pleurodesis (n=34), and re-VATS (n=5). The mean days of hospital staying were 5.3 days (3 to 10) in pleural drainage alone and 7.1 (4 to 20) in pleural drainage with chemical pleurodesis. 8 of 16 (50 %) with pleural drainage alone had recurrence and 15 of 34 (44.1%) with pleural drainage with chemical pleurodesis had recurrence. Chemical pleurodesis using a tetracycline did not show a significant difference in recurrence rate compared with pleural drainage alone (p=0.320).

**Conclusions**

Chemical pleurodesis using a tetracycline did not significantly decrease recurrence rate in management of postoperative recurred spontaneous pneumothorax compared with pleural drainage alone. Therefore, chemical pleurodesis with tetracycline to reduce recurrence rate should be reconsidered and further studies are needed to find out alternative drugs which reduce the recurrence rate statistically significantly.

## O34 Reducing OR cancellations and hospital cost: nurse –led intervention at a tertiary care hospital in LMIC

### Sadaaqat Ali, Mahim Akmal, Tahira Faiz

#### AKUH, Pakistan

##### **Correspondence:** Sadaaqat Ali

**Background**

Surgical cancellations at the day of surgery present major difficulties to both health care organizations as well patients and families. In a low middle income country (LMIC), cancellations stress an already resource limited system and result in significant financial burden and loss of resources. Due to a limited number of tertiary care hospitals, most families have to travel great distances and outpatient visits for preoperative assessment are unfeasible. We describe a protocol using screening phone calls and patient’s family counseling prior to admission to reduce same-day cancellations rates.

**Objective**

To determine whether implementation of nurse –led protocol involving screening phone call and patient’s family counseling 48 hours prior to surgery results in reducing day-of-surgery cancellations.

**Methods and measures**

Pre-intervention data to calculate rate of cancellations was collected. Patient’s families were called 48 hours prior to surgery. A standard assessment checklist was used to cover pertinent points. A brief counseling session was also carried out regarding the surgery .In case of any concerns, attending surgeon and cardiologist were informed and a follow up call made on the day before surgery. A satisfaction survey regarding the effectiveness of the phone call was carried out after discharge from the hospital.

**Results**

Post-intervention, data from 59 screened patients was collected. Day of surgery cancellation rate decreased from 18.5% to 1.8 %( p<0.05) (fig). An average cost of $200 (Pakistan Rupee 23150) was saved per cancellation. Most families reported overall satisfaction with the phone call.

**Conclusion**

Implementation of standardized protocol for preoperative assessment has decreased day of surgery cancellations, resulting in better resource utilization and reducing overall hospital costs. It has also helped in improving communication between patient’s family and heath care providers and improved overall patient satisfaction.

## O35 Root management strategy in Stanford A aortic dissection

### Yunxing Xue, Qing Zhou, Dongjin Wang

#### Department of Thoracic and Cardiovascular Surgery, The Affiliated Drum Tower Hospital of Nanjing University Medical School, Nanjing, China

##### **Correspondence:** Yunxing Xue

**Objective**

There is no standard method for root management in Stanford A aortic dissection (TAAD) involving the aortic root. We retrospective observed the results of different surgical methods for TAAD patients and analyzed the risk factors of aortic insufficiency (AI) and aortic dilation in the midterm follow-up period.

**Methods**

The clinic data of 351 acute Stanford A aortic dissection patients received surgical therapy at Department of Thoracic and Cardiovascular Surgery, Nanjing University Medical School Affiliated Nanjing Drum Tower Hospital from January 2008 to December 2015 were analyzed retrospectively. There were 272 male and 79 female patients, aging from 22 to 83 years with a mean age of (52±13) years. According to root size, aortic valve structure and the status of dissection involvement, these patients were devided into three major groups: 218 cases with root reconstruction using Dacron felts, 34 cases with root reconstruction concomitant with aortic valve resuspension repair and 99 cases in with Bentall procedure. Proper shape based on the status of dissection involvement of Dacron patch was cut and put between the middle and outerlayer of aorta, then inside the inner layerone band Dacron felt was sutured with the aorta and the new middle layer with Dacron patch as mentioned above. In some cases the prolapsed aortic valve were re-suspended to the aortic cusp. Clinical outcomes among the 3 procedures were compared by χ2 test and analysis of variance.

**Results**

Cross-clamp, cardiopulmonary bypass, and circulatory arrest times of all the patients were (250±78), (171±70) and (31±10) minutes, respectively. The 30-day mortality was 9.2% (33/351), while no difference among the 3 procedures (9.6%, 8.8% and 9.1%). In the average follow-up time of (26±23) months (range from 0.5 to 90.0 months), survival rates were similar among the 3 procedures (77.7%, 77.4% and 77.8%). Only one patient received redo Bentall procedure because of severe aortic regurgitation and dilated aortic root (50 mm).

**Conclusions**

The indication of root management of acute Stanford A aortic dissection is based on the diameter of aortic root, structure of aortic leaflets, and the dissection involvement. For most acute Stanford A aortic dissection patients, aortic root reconstruction is a feasible and safe method.

## O36 Surgical repair for acute myocardial infarction induced ventricular septal defect

### Dongjin Wang, Yunxing Xue, Qing Zhou

#### Department of Thoracic and Cardiovascular Surgery, The Affiliated Drum Tower Hospital of Nanjing University Medical School, Nanjing, China

##### **Correspondence:** Yunxing Xue

**Background**

Ventricular septal defect (VSD) induced by acute myocardial infarction (AMI) is rare but lethal with a high mortality even received surgical repair. Time interval from onset to operation is possibly beneficial for patients. Our aim was to assess the association of time interval with surgical repair effects for patients with VSD following AMI.

**Methods**

From January 2003 to December 2017, 14 patients with VSD induced by AMI have received surgical therapy in our department. We retrospectively reviewed the patients’ clinical manifestations, surgical methods, and outcomes. According to the time interval from AMI onset and operation, we divided into two groups, Group 1 (number=9) as more than one week and Group 2 (number=5) as less than one week. Comparing study was done and differences were analyzed.

**Results**

The mean age of the entire group was 65.5±3.3 years with male percentage of 78.6%(11/14). VSDs were anterior apical in 10 (71.4%) and posterior inferior in 4 (28.6%) patients. Average size of VSD was 15.8±5.8mm. Compared with Group 1, Group 2 revealed worse left ventricular function (LVEF 40.8±10.3% VS 30.4±2.3%, P=0.035), higher rate of urgent procedure (11.1% VS 100.0%, P=0.003). The cardiopulmonary bypass time and aortic clamp time was 203.9±52.3 and 152.4±44.8 minutes. All patients underwent concomitant coronary artery bypass graft surgery. The mortality rate was 14.3% (2/14), higher in Group 2 but no significant differences (20.0% VS 11.1%, P=1.000). Mechanical support (IABP and ECMO) were more common in Group 2, both preoperative (IABP, 22.2% VS 80.0%, P=0.091; ECMO, 0 VS 20.0%, P=0.357) and intraoperative period (IABP, 0 VS 60.0%, P=0.027; ECMO, 0 VS 40.0%, P=0.110). No resistant shunt and death was found during follow-up.

**Conclusions**

VSD following AMI is safer for more than one week, but surgical treatment is also acceptable for patients requiring urgent surgery due to hemodynamic instability. Mechanical assistive devices such as IABP and ECMO can improve perioperative success rate.

## O37 Antegrade implantation of previously fenestrated stent for arch repair for acute Stanford type A aortic dissection

### Qing Zhou, Yunxing Xue, Dongjin Wang

#### Department of Thoracic and Cardiovascular Surgery, The Affiliated Drum Tower Hospital of Nanjing University Medical School, Nanjing, China

##### **Correspondence:** Yunxing Xue

**Background**

The best surgical strategy for acute Stanford type A aortic dissection (aTAAD) involving the arch is controversial. We have used a novel method that antegrade implanting a previously fenestrated stent for arch repair, which have revealed acceptable results.

**Methods**

From December 2014 to December 2016, 81 aTAAD patients (52 male, 29 female) underwent ascending aorta replacement and fenestrated stent graft implantation. The fenestrated stent graft was implanted into the true lumen of aortic arch and proximal descending aorta with the fenestration opening at the ostia of three head vessels in the arch. The proximal end of the stent graft was anastomosed to the distal end of the Dacron tube graft that replaced the proximal ascending aorta. All patients had contrast enhanced computed tomography angiography before discharge and during follow up.

**Results**

The cardiopulmonary bypass time was 213 ± 49 minutes, aortic cross-clamp time was 133 ± 39 minutes, and selective cerebral perfusion and lower body arrest time was 27 ± 8 minutes. There were 5 in-hospital deaths due to circulation failure (mortality 6.2%). 5 patients died during follow-up period. The surviving patients had contrast enhanced CT scans in the 3rd, 6th, and 12th months. The flow up CT revealed increasing false lumen thrombosis.

**Conclusion**

In patients with aTADD, the previously fenestrated stent graft results in excellent aortic remodeling of the aortic arch and descending aorta without increasing morbidity and mortality.

## O38 Results of false lumen status of different methods for aortic arch treatment in Stanford A aortic dissection

### Qing Zhou, Yunxing Xue, Dongjin Wang

#### Department of Thoracic and Cardiovascular Surgery, The Affiliated Drum Tower Hospital of Nanjing University Medical School, Nanjing, China

##### **Correspondence:** Yunxing Xue

**Objective**

Different methods are applied for aortic arch treatment in Stanford A aortic dissection, but it’s lack of long term results of false lumen status of different methods. This retrospectively study will analyze the effect of the false lumen status of different methods in a single center.

**Methods**

From January 2010 to December 2015, 139 cases(of which 108 malesand 31 females) were finally selected after excluding the cases who died during hospitalization, whose perioperative clinical data were incomplete, follow-up information were incomplete, and DeBakey type II aortic dissection and the cases with descending aorta dilatation. The average age was 50.3 ± 11.6 years (22-76 years). According to the methods for aortic arch and descending aorta, 139 cases were divided into 5 groups: 24 cases in AR((including ascending aorta replacement, ascending aorta + hemi-arch replacement and ascending aorta + island-arch replacement), 9 cases in AR+SET(including ascending aorta + hemi-arch replacement + stent elephant trunk and ascending aorta + island-arch replacement + stent elephant trunk), 42 cases in Arch+SET(ascending aorta + arch replacement + stent elephant trunk), 22 cases in AR+ TBS(ascending aorta + triple branched stent) and 42 cases in AR+FS(ascending aorta + arch fenestrated stent). Statistical analysis the size of true lumen and the status of false lumen among these five groups in the level of aortic arch, the distal end of stent, diaphragm, celiac artery, renal artery and iliac artery postoperatively.

**Results**

Different levels of descending aorta in each group have showed varying degrees of true lumen open and thrombosis of false lumen during follow-up period. Among them, cases with aortic arch treatment and stent implantation have showed higher ratio of thrombosis of false lumen than AR group. The thrombosis of false lumen in the arch level was higher in the Arch+SET group and AR+FS group, while AR+TBS and AR+SET group all showed decreased ratio of thrombosis of false lumen.

**Conclusions**

Effects of different stents were similar, which all promoted the process of thrombosis of false lumen.

## O39 Axillary-carotid bypass with TEVAR for complex arch disease - traditional method to solve difficult problem

### Shuchun Li, Yunxing Xue, Qing Zhou, Dongjin Wang

#### Department of Thoracic and Cardiovascular Surgery, The Affiliated Drum Tower Hospital of Nanjing University Medical School, Nanjing, China

##### **Correspondence:** Yunxing Xue

**Objective**

Thoracic endovascular aortic replacement (TEVAR) has become a routine treatment for aortic disease with good results and low mortality and morbidity. But TEVAR surgery involving the arch needs to consider the treatment of the supra-arch vessels. This article will retrospectively analyze the single center’s experiences about the technique of using axillary-carotid bypass method to extend the landing zone for preserving the supra-arch vessels.

**Methods**

From January 2015 to December 2018, 285 patients with aortic disease (dissection, aneurysm) were treated using TEVAR in our center. Among the 285 cases, 65 patients (22.8%) were involving the aortic arch. Among them, 42 cases (64.6%) with axillary-carotid bypass, 3 cases (4.6%) with two staged de-branch procedure (supra-arch vessels debranch with artificial vessels), 2 cases (3.1%) with chimney method, and 14 cases (21.5%) with physician-modified fenestrated stent technique. Retrospective statistical analysis of the clinical features, technical methods and follow-up results of patients with axillary-carotid bypass method. All patients have taken orally aspirin for 3 months after the operation.

**Results**

The patient's lesion type included 28 cases of aortic dissection, 9 cases of penetrating ulcer, and 5 cases of aortic aneurysm. There were 11 cases involving Zone-2 and 31 cases involving Zone-3. There were no deaths during both perioperative period and follow-up term, the technical success rate was 100%. There was no perioperative stroke or paraplegia. The average follow-up time was 14 months, and the CT confirmed that all bypass patients with unobstructed flow in the artificial blood vessels during follow-up term. No re-intervention was observed.

**Conclusions**

The axillary-carotid bypass method is a simple surgical method and the technical success rate is high. Although the trauma risk is increased, there is no additional increase in mortality and complication morbidity rates. It can be used as a technical method to preserve the supra-arch vessels in TEVAR operation.

## O40 Modified left ventricular reconstruction method for ischemic heart disease with left ventricular aneurysm

### Jun Pan, Yunxing Xue, Qing Zhou, Dongjin Wang

#### Department of Thoracic and Cardiovascular Surgery, The Affiliated Drum Tower Hospital of Nanjing University Medical School, Nanjing, China

##### **Correspondence:** Yunxing Xue

**Objective**

Left ventricular aneurysm is one of the complications after myocardial infarction, and traditional surgery has certain limitations. To summarize the clinical effects and follow-up results of our center using modified left ventricular reconstruction surgery for left ventricular aneurysm.

**Method**

From January 2002 to December 2016, we performed a modified left ventricular reconstruction for 33 patients (22 males and 11 females) with post myocardial infarction ventricular aneurysm. The average age of 56.8 (48-65) years old. The preoperative ventricular aneurysm size was 62.8 mm with an average left ventricular end diastolic diameter and an average EF of 34.7%. All patients underwent a modified left ventricular reconstruction surgery, that is, after the ventricular aneurysm was opened and the boundary was defined, a suitable size of polyester sheet was lining the ventricle cavity, and the purse was sutured after suturing the border after resection. The outer layer was sutured continuously with two lengths of 3-5 cm felt strips. 25 patients underwent coronary artery bypass surgery. 18 patients underwent ventricular septal perforation repair. 13 patients underwent mitral valvuloplasty and 3 patients underwent mitral valve replacement.

**Result**

Perioperative mortality was 9.1% (3/33), with an average follow-up of 72.9 (1-171) months. There was no death during follow-up and there was no reoperation due to mitral regurgitation. At the postoperative follow-up, the mean left ventricular end-diastolic diameter was 58.8 mm, with an average EF of 39.4%.

**Conclusions**

Modified left ventricular reconstruction surgery is performed at the same time as the removal of ventricular aneurysm, and the effect of left ventricular remodeling and ventricular systolic function retention is clear. Perioperative, long-term follow-up results showed satisfactory results. At the same time, revascularization does not affect the surgical outcome of this group of patients.

## O41 Clinical outcomes of different locations of primary intimal tear in Stanford A aortic dissection

### Chang Liu, Yunxing Xue, Qing Zhou, Dongjin Wang

#### Department of Thoracic and Cardiovascular Surgery, The Affiliated Drum Tower Hospital of Nanjing University Medical School, Nanjing, China

##### **Correspondence:** Yunxing Xue

**Objective**

Primary intimal tear is the leading cause of aortic dissection. The relationships between the location of primary intimal tear in Stanford A aortic dissection and the clinical manifestation and outcomes are not clearly.

**Methods**

From January 2011 to December 2016, 476 TAAD patients (365 male, 111 female) were enrolled in this retrospective research. With the aid of Preoperative CT scan and intraoperative observing, we confirmed the location of primary intimal tear of all patients, the results revealed that 229 patients with primary intimal tear in the ascending aorta (Group As), 90 patients located in the aortic arch (Group Ar) and 157 patients in the other site or with multiple location (Group O). The clinical data and perioperative information were analyzed related to the location of primary intimal intimal.

**Results**

The ratio of hypertension was higher in the Group Ar (84.44%, p=0.06), while the ratio of Marfan syndrome was significantly lower in the Group O (0.64%, p=0.06). Cerebral malperfusion distributed differently and more predominant in Group As and Group Ar, and more patients with aortic valve regurgitation (Grade≥2) were in Group As. As different range of dissection inducing by different location of intimal tear, we have performed different surgical methods. More root replacement operations in Group Ar and more total arch replacement in Group As. With similar operative time, ICU stay was shorter in Group Ar. Mortality and morbidity were similar in all patients. Logistic regression analysis showed that risk factors for postoperative death included cardiac tamponade (p=0.02 OR=4.66 CI 1.25-17.44), coronary perfusion malperfusion (p=0.05 OR=2.67 CI 0.96-7.39) and cardiopulmonary bypass time (p= 0.01 OR=1.01 CI 1.00-1.02). The causes of death mainly include multiple organ dysfunction, circulatory failure and respiratory infections.

**Conclusion**

The clinical manifestation and clinical outcomes were different according to the location of primary intimal tear location in patients with Stanford A aortic dissection.

## O42 Trans-apical transcatheter aortic valve replacement in complex aortic valve disease with J-Valve system

### Qing Zhou, Yunxing Xue, Shuchun Li, Dongjin Wang

#### Department of Thoracic and Cardiovascular Surgery, The Affiliated Drum Tower Hospital of Nanjing University Medical School, Nanjing, China

##### **Correspondence:** Yunxing Xue

**Objective**

J-Valve system is one special designed for trans-apical TAVR (Transcatheter aortic valve replacement) with three U-shape graspers. With the unique structure, J-Valve is suitable for both aortic stenosis and aortic regurgitation. We used the J-Valve system for patients with severe peripheral vessel disease and/or with artic regurgitation and received good clinical results.

**Methods**

From January 2018 to December 2018, 12 patients (6 male, 6 female) underwent trans-apical transcatheter aortic valve replacement using J-Valve system. High risk patients with diseased peripheral vessel (fragile or calcification) and with valve disease aortic regurgitation were enrolled. 11 patients were suffered with both aortic stenosis (≥2,0-4) and aortic regurgitation (≥2,0-4), only one patient was only aortic stenosis but she had severe calcified aorta (porcelain aorta). The average preoperative STS score and EuroScore was 12.0% and 9.80%, respectively. There patients had prior heart surgery history and one of them received bioprosthetic aortic valve valve-in-valve therapy.

**Results**

The technique success rate was 100% and one patient was dead because of heart failure after TAVI (mortality 8.33%). One patient suffered stroke and recovered well, no more other complication was observed perioperative. During the follow-up period, one patient was died because of acute heart attack. Other 11 patients completed TTE check. The rate of perivalvular leak (larger than moderate) was 0% and the rate of mild and trace leak was 18.18%. The postoperative average flow rate was 2.1m/s and average transvalvular pressure difference was 15mmHg.

**Conclusions**

J-Valve system is a safe and effect method for high risk aortic valve disease patients not suitable for peripheral routine TAVI.

## O43 Modified veneplication technique a modo Topalov-Guirov for prophylaxis of venous thromboembolism

### Dragomir Draganov, Iovcho Topalaov ^1^, Kuzman Guirov^2^, Svetozar Marangozov^2^

#### ^1^University hospital “Heart and brain”, Pleven, Bulgaria; ^2^Department of Vascular surgery and angiology, Military Medical Academy, Sofia, Bulgaria

##### **Correspondence:** Dragomir Draganov

The venous thromboembolism (VTE) is one persistent problem that still hasn’t found its solution. The authors share their experience in the prophylaxis of VTE patients with embologenic clots, distal from external iliac vein . The widespread opinion is that in those cases there are no recommendations for the implantation of a vena cava filter which to require any kind of anticoagulant therapy with a direct anticoagulant for the rest of the patient’s life. The aforementioned methodic is not relatively new – the plication of the femoral vein is a well-known technique in the prophylaxis of PTE. The authors modify this method in a way in which the non-resorbable monofilament suture materials are replaced by resorbable, monofilament threads which degrade after a period of 3 to 6 months. The other modification of the same methodic is that the sutured walls of the vein are not in contact with each other, thus the surgical threads form a vast grid in the lumen of the vessel that is enough to stop the embologenic blood clot. The experience of the authors is based on 63 patients , all suffering from DVT and monitored for a period of 1 to 3 years. None of those patients manifested any sort of a PTE complication. The anticoagulant therapy shows no signs of alteration in regards to the classical therapy in such cases. The authors can safely conclude that the displayed method of surgical prophylaxis is a lot more sparing for the patient’s well-being rather than the implantation of a vena cava filter, including a one which can be removed after a particular length of time.

## O44 Is the late peripheral catheter embolectomy a procedure with limited potential?

### Lefter Nasto^1^, Tanyo Kavrakov^1^, Valentin Vasilev^2^

#### ^1^Clinic of Vascular Surgery, University Hospital, Stara Zagora, Bulgaria; ^2^Clinic of Vascular Surgery, University Hospital, Burgas, Bulgaria

##### **Correspondence:** Lefter Nasto

**Introduction**

Peripheral embolic ischemia of the extremities is a condition of acutely decreased blood flow, due to a occluded artery, which leads to ischemic tissue damage, that can threaten the limb of a patient and requires immediate revascularization. Accurate and timely diagnosis is important to salvage the patient’s limb and sometimes the patient’s life. Surgical catheter embolectomy has been the treatment of choice for arterial embolism for more than 50 years, but its efficacy and predictive factors for limb salvage are closely related with duration from symptom onset to surgery. Depending of the time from the onset of the symptoms arterial embolectomies could be divided on: Early embolectomy - 8 to 12 hours after onset is considered the optimal time for this procedure. Late embolectomy - 12 hours after onset of the symptoms.

**Methods**

We have reviewed 373 arterial embolectomies performed for a period of 10 years, computer records, operative journals and medical documentation of these patients were retrospectively studied and recorded. 26 patients treated between May 2016 - February 2019 with diagnosis of late arterial embolism, untreated at least 12 hours from the onset of symptoms were analyzed.

The patients were divided in 4 groups according to the stage of AAI.

**Results**

All of the patients who admitted with I stage - viable ischemia had excellent post treatment outcome.

6 patients with IIa stage of AAI were with salvage extremities, 2 patients with PAD were with absent peripheral pulses.

Overall mortality rate in group with IIb - immediate ischemia was 44% ( 4 of 9 ), necessity of amputation was 11% and 50% of operated patients in this group were with normal arterial pulses post operatively and no complains.

In patients with stage III - irreversible ischemia mortality rate was 14% ( 1 of 7 ), amputation rate 71% ( 5 of 7 ), 2 patients had developed a demarcation line on the forefoot, whereat we decided to perform one-step late embolectomy and transmetatarsal amputation.

**Conclusion**

According to our observation, early and late post-treatment follow-up, clinical and operative experience, appending the general status and comorbidity of the patients, we educe that there isn’t a strong correlation between delay in embolectomy the mortality and limb salvage rates.

Despite the controversy among the authors and vascular surgeons performing late embolectomy at selective group of patients is recommended and could be beneficiall.

It could reduce amputations and contributes to improving the quality of life among the affected patients.

## O45 Application of extracorporeal membrane oxygenation during percutaneous coronary interventions in patients with acute coronary syndrome

### Borys Todurov^1^, Andrii Khokhlov^1^, Olexandr Druzhyna^1,2^, Oleh Loskutov^1,2^, Olexandr Postupalskyi^1^, Stepan Maruniak^1,2^, Sergii Veremchuk^2^

#### ^1^State Institution “Heart Institute Ministry of the Health of Ukraine”, Kyiv, Ukraine; ^2^Department of Anesthesiology and Intensive Care, Shupyk NMAPE, Kyiv, Ukraine

##### **Correspondence:** Stepan Maruniak

**Introduction**

To date, surgical interventions – percutaneous coronary intervention (PCI) and coronary artery bypass grafting (CABG) are the main method of treatment of acute coronary syndrome. At the same time, PCI in patients with acute heart failure may be accompanied by significant hemodynamic instability, which requires the use of additional methods to life support, such as extracorporeal membrane oxygenation (ECMO).

**The aim**

To evaluate the effectiveness of application of ECMO in PCI in patients with acute coronary syndrome with acute heart failure.

Materials and methods: This retrospective study included patients who had undergone PCI with the application of ECMO in the Heart Institute Ministry of Health of Ukraine during 2014-2018. Hemodynamic and biochemical parameters, ECMO application time, mortality and complications were analyzed.

**Results**

According to the analysis, ECMO was used during endovascular recanalization in 23 cases (0.4%). At that, the share of men was 82.6%, women – 17.4%, the average age – 65.2 ± 7.1 years. All patients were in Killip class IV. The main infarct-related artery was the anterior interventricular branch of the left coronary artery and TIMI 0 was determined in 11 patients (47.8%).

In 9 (39.2%) cases ECMO was applicated to the beginning of PCI, and in 13 (60.8%) – during the procedure for cardiac arrest (5 (37,5%) patients) and for the development of life-threatening arrhythmias (9 (62,5%) patients).

All 9 patients treated with ECMO before to the beginning of PCI were discharged, while in patients, which ECMO was connected during the PCI, the hospital mortality rate was 92.3%.

**Conclusions**

The prophylactic application of ECMO during PCI in patients with acute heart failure gives good results of short-term survival. Further research with a larger sample size is needed to fully assess its efficacy.

## O46 Ten years experience with re-do adult cardiac surgery: single centre retrospective study

### Ivilin Todorov, Georgi Vasilev, Veneta Grigorova, Dimitar Nikolov

#### Clinic of Cardiac Surgery, Acibadem City Clinic Tokuda Hospital, Sofia, Bulgaria

##### **Correspondence:** Ivilin Todorov

**Background**

An increasing number of patients are being referred for re-do cardiac surgery and will continue to increase.This patients have a specific set of problems both for the surgeon and the anesthesiologist such as in gaining sufficient exposure, limiting blood loss, associated pulmonary hypertension, valvular dysfunction and require special attention at all stages of management. Patients who need Re-do cardiac surgery are frequently compromised and have a little reserve to compensate the surgical related trauma and other evolving problems.

**Aim**

To study the in hospital outcome of patients undergoing re-do cardiac surgery in a single center.

Methods: Data was collected from 330 patients for 10 years period. Only adult re-do's were included in this retrospective study. The following factors were collected in the retrospective study: duration since previous surgery, intraoperative complications, postoperative complications, duration of CPB and cross clamping time, duration of ventilation, amount of blood loss, site of canulation , surgical approach- sternotomy or thoracotomy, intra aortic balloon pump insertion, need of renal replacement therapy, tracheostomy and total circulatory arrest.

**Result**

The mean age was 64.10 years; 198 (60%) male, 132 (40%) female. The mean time from previous operation was 90 months . The mean duration of cardiopulmonary bypass time was 114.64 minutes (20-333 minutes), mean cross clamp time was 64.17 minutes (10-209 minutes). The duration of total circulatory arrest ranged from 8-80 minutes.The mean duration of ventilation was about 42.52 hours (1hr to 1368 hrs). Re-exploration for bleeding was required in 30 patients (9.09%). Mean chest tube drainage was about 682.50 ml. Sixty-two patients need postoperative renal substitution therapy (18.7%).Mean duration of in hospital stay was 10.81 days (5 days to 136 days). The mortality rate was 15.45% (51 out of 330 patients).

**Conclusion**

Redo surgery patients are with increased risk of morbidity and mortality than first-time surgery patients and this sub-group of patients can present new challenges especially when non-elective and multiple procedures are warranted.

## O47 Isolation of pulmonary veins in patients with atrial fibrillation without mitral valve disease undergoing elective heart surgery

### Dimitar Kyuchukov, Boyan Baev, Rumen Iliev, Stanislava Stoycheva, Gencho Nachev

#### Univerity Hospital “St.Ekaterina”, Medical University, Sofia, Bulgaria

##### **Correspondence:** Dimitar Kyuchukov

Atrial fibrillation (AF) is one of the major cause of mortality and morbidity, especially in patients with heart operation. The aim of this study was to evaluate the efficacy of bipolar radiofrequency isolation of pulmonary veins (PVI) in patients with atrial fibrillation without mitral valve disease undergoing cardiac surgery with cardiopulmonary baypass (CPB).

**Materials and Methods**

The study covers a period of 12 years from 2005 to 2017 and includes 51 patients with permanent or persistent AF underwent an open heart surgery for aortic valve disease or coronary artery disease. The inclusion of patients is prospective. All patients were operated under CBP with aortic x-clamping and cardioplegical arrest with blood cardioplegia delivered ante- and retrograde fashion according to our hospital protocol. The average age of patients was 63.45 (52-81) years. 32 (62.7%) of them were men. The PVI performance was the main criterion according to whom the patients were divided into two groups. The control group included 22 (43.1%) patients operated during first two years of the period from Jan.2005 to Oct.2007 and had no PVI procedure. The group of patients underwent PVI were operated between Nov. 2007 to Mar.2017 and included 29 (56.9%) patients. All patients were followed for the rhythm type and left ventricle ejection fraction (LVEF) on the first, sixth and twelfth months.

**Results**

With comparable pre- and intraoperative surgical characteristics, patients with PVI showed statistically superior levels of successful sinus rhythm conversion reaching nearly 90% immediately after the surgery and successfully sustained in 67% by the end of the follow-up period. No difference in LVEF alterations in both groups. No perioperative complications registered and the mortality rate in both groups was zero.

Conclusion: Performing PVI is a safe and effective method for treating AF.

**Key words**

Atrial fibrillation, Pulmonary vein isolation, Sinus rhythm, operation under extracorporeal blood circulation.

## O48 The role of postoperative Trimetazidine therapy on myocardial preservation and graft patency in coronary artery bypass surgery patients

### Asen Ivanov^1,2^, Zaprin Vazhev^1,2^, Todor Gonovski^1,2^, Hristo Stoev^1,2^, Konstantin Dimitrov^1,2^

#### ^1^Department of Cardiovascular Surgery, Medical University, Plovdiv, Bulgaria; ^2^**“**St. George**”** University Hospital, Plovdiv, Bulgaria

##### **Correspondence:** Asen Ivanov

**Objective**

Coronary artery bypass surgery remains the gold standard in treatment of patients with ischemic heart disease. However, the increased oxidative stress caused by the release of free radicals during the ischemia-reperfusion time is a well-known pathophysiological process during and after coronary revascularization procedures. It may lead to reversible and irreversible myocardial injury. The focus of this prospective single-blinded randomized controlled trial is to investigate and analyze the effectiveness of the drug trimetazidine on reducing the postoperative myocardial ischemia-reperfusion injury as well as to evaluate the impact on the early graft patency.

**Methods**

The study included 90 patients divided into two subgroups operated on elective manner receiving isolated coronary artery bypass surgery between March 2018 and October 2018. The first subgroup received a regular therapy of 35mg trimetazidine in a regimen twice daily immediately after the extubation. The rest of the medication therapy was identical to all of the participants. Preoperative and postoperative levels of several blood-based biochemical markers including malondialdehyde(MDA), creatinine kinase-MB fraction(CK-MB) and troponin T(TnT) were measured. The data was classified and analyzed by the timing of sample collection: 12 hours before and 6 months after the surgical procedure.

**Results**

The data suggests that postoperative treatment with trimetazidine leads to decrease in MDA production, CK-MB and TnT levels therefore to oxidative stress reduction and better myocardial cell protection by antioxidant status augmentation. The follow-up was 6 months after the surgery. In total, 90 control percutaneous coronary angiographies were performed with 0% cases of graft failure from 270 total distal anastomoses performed. The Quality of life assessment by Minnesota Living with Heart Failure Questionnaire was conducted and revealed excellent results. Conclusions: Postoperative trimetazidine therapy leads to improvement of the myocardial cell metabolism and thus reduction in post CABG ischemia-reperfusion injury. The long-term therapy effects on graft patency and systolic ventricular function remain to be seen.

**Keywords**

Trimetazidine, coronary artery bypass grafting, oxidative stress, postoperative results.

## O49 Medistinal and cervical evolution of pancreatic pseudo-cysts after acute pancreatitis

### Cvetan Minchev^1^, Emanuil Manolov^1^, Anton Angelov^1^, Slavi Bizyokov^1^, Petko Karagyozov^2^

#### ^1^Thoracic Surgery Department, Acibadem City Clinic Tokuda Hospital, Sofia, Bulgaria; ^2^Gastroenterology Department, Acibadem City Clinic Tokuda Hospital, Sofia, Bulgaria

##### **Correspondence:** Slavi Bizyokov

**Background**

Pancreatic cysts are real cysts and pseudo-cysts. True pancreatic cysts occur very rarely. They have an epithelial capsule and are usually congenital. Pancreas pseudo-cysts that are without their own epithelial capsule are more common. According to their causes, they are often inflammatory, traumatic, retentive, parasitic and neoplastic.

**Case report**

We represent a 47-year-old woman with unclear ascites, abdominal pain and weight reduction. A history of total laparo-hysterectomy with adnexectomy, in relation to uterine leomyomas, adenomyosis, with no evidence of malignancy. She goes to Gastroenterology department with CT data for pseudo-cysts of the pancreas with mediastinal evolution. Laboratory, imaging and endoscopic studies performed. established chronic calcification pancreatitis, presence of one pancreatic and one peripancreatic fluid collection, ascites, thrombosis of v porta. During the hospitalization, the patient develops dyspnea, performed chest CT, established pleural and mediastinal collections with high levels of pancreatic enzymes.Translated into a thoracic surgery department, where left VATS was performed and drainage to the right, mediastinotomy and evacuated pleural collections. From biochemical analysis - high content of pancreatic enzymes. A few days later, due to complaints of shortness of breath and swelling of the neck with pain and edema, we performed cervicotomy, mediastinotomy and paraesophageal posterior mediastinum drainage.Due to constant secretion from the drains, gastroenterologists performed pancreatic sphincterotomy; endoscopic prosthesis of ductus pancreaticus, transgastric drainage of peripancreatic fluid collection; biliary sphincterotomy; ndoscopic prosthesis of ductus choledochus. On the second day after the procedure - without secretion from the mediastinal drains, which were removed on the 3rd postoperative day. 6 months after the described treatment, the patient is in a very good general condition with normalization of the weight.

**Conclusions**

The case report presented here is an example of good collaboration between interdisciplinary teams with a view to adequate treatment of complicated pseudo-cysts with mediastinal and cervical evolution after pancreatitis using modern video-assisted and endoscopic maneuvers.

The patient provided written consent to the publication of clinical or personal details.

## O50 Multimodal low-dose opioid anesthesia in elderly patients undergoing coronary artery bypass grafting with cardiopulmonary bypass

### Taisiia Danchyna^2,3^, Borys Todurov^1,2^, Oleh Loskutov^1,2^, Druzhyna Oleksandr^1,2^, Volodymyr Kolesnykov^1,2^, Bohdan Dimov^1,2^

#### ^1^“Heart Institute Ministry of Health of Ukraine”, Kyiv, Ukraine; ^2^Anesthesiology and Intensive Care department Shupyk NMAPE, Kyiv, Ukraine; ^3^National Military Medical Clinical Centre, Kyiv, Ukraine

##### **Correspondence:** Bohdan Dimov

**Aim**

Study of efficiency of multimodal low-dose opioid general anesthesia in elderly patients, undergoing coronary artery bypass grafting (CABG).

**Materials**

The study included 36 patients, aged 69.5±6.2 years, undergoing CABG under general anesthesia with cardiopulmonary bypass (CPB). Anesthesia was maintained with sevoflurane 1.5-2 MAC, ketamine 0.5 mg/kg, IV lidocaine infusion 1.5-2 mg/kg/h. Fentanyl was administered at the main pain stages of the operation. Level of the stress response was determined by measuring of lactate and cortisol in blood. Postoperative pain assessment was based on Visual Analogue Scale (VAS).

**Results**

The average duration of anesthesia was 257,4±19,1 min., CPB - 55±10min. Average total fentanyl dose was 0.4±0.03 mcg/kg/h. Hemodynamic and bispectral index parameters (BIS=42.25±1.6) indicated an adequate anesthesia.

Stress indicators were within the normal range (average cortisol value=479.3±26.4 nmol/l, lactate=1.6±0.2 mmol/l, glucose 6.42±0.9 mmol/l).

Positive verbal contact was after 18.6±3.4 min. after termination of sevoflurane inhalation.

All patients were extubated during first 3 hours after the end of the operation.

Blood gases analyses were satisfactory, with no significant changes.

Postoperative analgesia provided by tromethamine ketorolac IM 30 mg every 8-12 hours for 2 days. The average VAS pain level was 4.6±1.2 points.

**Conclusions**

It was found that multimodal low-opioid anesthesia technique according to the proposed scheme provided an adequate analgesic effect and allowed us to refuse the intraoperative use of routine doses of fentanyl during CABG with CPB, which was confirmed by the absence of hemodynamic and endocrine-metabolic shifts when we performed it.

## O51 Tracheal resections and reconstructions for primary Adenoid cystic carcinoma - 12 years of experience and follow up

### Tsvetan Minchev^1^, Emanuil Manolov^1^, Asen Kelchev^2^, Anton Angelov^1^, Slavi Bizokov^1^, Margarita Kateva^3^

#### ^1^Acibadem City Clinic Tokuda Hospital, Thoracic surgery, Sofia, Bulgaria; ^2^Acibadem City Clinic, Cardio Surgery, Sofia, Bulgaria; ^3^UMBAL Sofia Med, Reconstructive surgery, Sofia, Bulgaria

##### **Correspondence:** Tsvetan Minchev

**Background**

Adenoid cystic carcinoma (ACC) is the second-most common primary malignant tracheal neoplasm after squamous cell carcinoma, and it is a low-malignancy neoplasm having a prolonged clinical course. We present tracheal resections and reconstructive operations for ACC and patient follow up. We present also total tracheolaryngeal resection for ACC and trachea substitution.

**Patients and methods**

This are a prospective study over a 12-year period. Fifteen patients with resection surgery for ACC, aged 36 to 68 years was operated. Proximal resection was performed in 5 patients with a distal at 8. A left pulmonemectomy with carina resections with AV ECMO and a laryngotracheal resection with total trachea substitution with a vascularized skin flap were performed. The operative access was cervicotomy 5 and 10 with sternotomy.

**Results**

No intra and preoperative mortality. The average length of the resected trachea is 3.5 cm (3 to 5.5 cm). In one patient, the whole trachea and larynx are resected. All patients without one have R0 resection lines. Control bronchoscopy in 3, 7 postoperative day was performed. Frequent bronchoaspiration was required in the patient with a tracheal substitution. All patients performed postoperative radiotherapy. They are tracked for 1 to 11 years with bronchoscopy, CT and PET over a different period of time. All alive and up to now. One patient had a local relapse 8 years after surgery.

**Conclusion**

ACC is a rare primary tracheal malignancy. This disease is commonly misdiagnosed as asthma. Surgical resection followed by radiotherapy is widely recommended protocol for treatment of localized tracheal tumours and provides the best chance of prolonged survival. Tracheal substitution Is method of choice for extended tracheal resections with excellent postoperative result.

## O52 VATS/ Laparoscopic resection of the oesophagus and gastroplasty - the initial experience with 20 patients

### Tsvetan Minchev^1^, Veselin Marinov^2^, Emanuil Manolov^1^, Anton Angelov^1^, Slavi Bizokov^1^

#### ^1^Thoracic surgery, Acibadem City Clinic, Tokuda Hospital, Sofia, Bulgaria; ^2^General surgery, Acibadem City Clinic, Tokuda Hospital, Sofia, Bulgaria

##### **Correspondence:** Tsvetan Minchev

**Background**

Esophageal cancer is a common malignancy, for which surgery is the most effective treatment. Compared with traditional surgery, video-assisted thoracoscopic and laparoscopy minimally invasive surgery enables less trauma, better visibility, reduced bleeding and postoperative pain, and lower incidence of surgical complications through a minimally invasive, safe, and highly cost-effective approach in favor of early rehabilitation after surgery. We have performed video-assisted thoracoscopic and laparoscopy minimally invasive surgery for 20 patients in our hospital for a period of 3 years.

**Methods**

For a period of last 3 years 20 patients underwent minimally invasive esophagectomy. No patient selection for tumor size and localization. There were 3 females. The mean age was 65 years. Indications for surgery were oesophageal cancer (n = 8), and gastric cardia cancer (n = 12). Surgical approaches included thoracoscopic/laparoscopic esophagectomy with a cervical anastomosis (n = 8), minimally invasive Ivor Lewis esophagectomy (n = 12).

**Results**

Median operative time was 6.5 hours. Median intensive care unit stay was 1 day; median length of stay was 7 days with no operative or hospital mortalities. No perioperative mortality. There were 2 major complication respectively: partial gastric necrosis in one patient treated with Ovesco clip and one carinal necrosis treated with omentoplasty.

**Conclusions**

Minimally invasive esophagectomy is technically feasible and safe in our center, Therefore, the promotion and application of this surgical approach will undoubtedly benefit the majority of patients with esophageal cancer.

## O53 Contemporary results of mitral valve repair – a nine-year experience

### Georgi Manchev, Vassil Gegouskov, Valya Goranovska, Boyan Markov

#### Division of Cardiac Surgery, St. Anna University Hospital, Sofia, Bulgaria

##### **Correspondence:** Georgi Manchev

**Objective**

We sought to review the early and late outcomes of mitral valve reconstructive surgery in patients with various etiologies of mitral regurgitation.

**Methods**

326 patients underwent mitral valve repair as a standalone or combined procedure in the period of 2010 – 2018. Median age was 67 years. The majority of patients had ischemic mitral regurgitation (44.5%) and a lesser proportion had degenerative mitral valve disease (26.7%). All patients except one had an annuloplasty ring/band implanted. Construction of neochords, leaflet resection and suturing techniques, or chordal transection were adjunctive procedures. 17.1% of patients were followed up by echocardiographic assessment with a mean interval from the operation 34.8 months (3 months to 8.2 years). Survival data was obtained through pnone calls, local, and national patient registries.

**Results**

Early functional class improvement was demonstrated in 89% of patients. 85.6% of patients were discharged with no or trivial mitral regurgitation. 23.2 % of patients were readmitted during the follow-up period due to progressive mitral regurgitation. 5 reoperations were performed at our institution for recurrent mitral valve disease. Overall survival at 2, 5, and 9 years, was, 91, 77, and 56%, respectively.

**Conclusion**

Mitral valve repair is a viable alternative to mitral valve replacement in an ever sicker mitral valve patient. In some patients recurrent mitral valve dysfunction is inevitable and expectable. However, primary mitral valve replacement should be reserved for the most advanced mitral valve pathology.

## O54 Resection of locally advanced lung cancer with cardiopulmonary bypass

### Tsvetan Minchev^1^, Dimitar Nikolov^2^, Ivilin Todorov^2^, Veselin Georgiev^2^, Georgi Vasilev^2^, Plamen Purvanov^2^, Veneta Grigorova^2^, Emanuil Manolov^1^

#### ^1^Acibadem City Clinic, Tokuda Hospital, Thoracic surgery, Sofia, Bulgaria; ^2^Acibadem City Clinic, Tokuda Hospital, Cardio Surgery, Sofia, Bulgaria

##### **Correspondence:** Tsvetan Minchev

**Background**

Resection of non-small cell lung cancer (NSCLC) on cardiopulmonary bypass (CPB) has rarely been reported in the literature. Hence, we have reviewed our experience in the role of CPB for the surgical treatment of locally advanced NSCLC.

**Methods**

All patients undergoing lung resection for bronchogenic carcinoma on CPB/ECMO in our institution for a period of 12 years.

**Results**

Тwenty-one patients underwent lung resections on CPB for bronchogenic carcinoma during the study period. Cardiopulmonary bypass was performed for tumors invading carina (left carinal pulmonectomy n=14) the superior vena cava (n = 2), the descending aorta (n = 1), or the infiltration of the left pulmonary artery (n=2) with the left atrium (n = 2). All patients were discharged home after 5 to 8 days. One patient died postoperatively with ascending thrombosis of jugular vein. Two patients are alive with recurrent disease at follow-up 24 and 50 months post-surgery, respectively. One patients have brain metastases 2 mounts after surgery. In the long term all other patient alive at the moment and are tracked over a period of 3 months to 10 years.

**Conclusions**

This study confirms the safety of CPB for NSCLC invading the great vessels and/or the left atrium in well-selected patients, and its utility for ventilation support during left pulmonectomy and carinal resection.

## O55 Sizing the aortic annulus, valve profiles and types of heart block related to Edwards Intuity sutureless bioprosthesis implantation

### Weon Yong Lee, Byung Mo Gu, Ho Hyun Ko

#### Cardiothoracic surgery, Hallym university sacred heart hospital, Anyang, Korea

##### **Correspondence:** Weon Yong Lee

**Background**

Sutureless aortic valve implantation has been an alternative to conventional aortic valve replacement. We compared the differences between echocardiographic measurement of aortic annulus and the size of operatively used prostheses, and evaluated the effectiveness of Edwards Intuity Sutureless aortic valve (Intuity valve) related to valve profiles and types of heart block occurred after implantation

Materials and Methods : Intuity valves were implanted in 5 patients with aortic stenosis between March, 2017 and December 2018. The mean age was 76.2 years, and female patients were 80% (4/5). They all had 3 aortic leaflets with calcific degeneration. Preoperatively, the maximal/mean pressure gradients were 93.2/58.8 mmHg. The mean aortic valve area was 0.58±0.08 cm2 (0.40±0.10 cm2/m2). The mean aortic annular size measured by echocardiography was 19.58±1.62 mm. Their cardiac rhythms were normal sinus and only one patient had 1st degree atrioventricular block preoperatively.

**Results**

There were no surgical mortalities and major complications. The size of implanted valves were 23mm in 1, 21mm in 2, and 19mm in 2 patients. The sizes of used prosthesis were approximately 1 mm larger than echocardiographic measurements of aortic annulus. Postoperatively, 1 patient with preexisting 1st degree heart block experienced complete atrioventricular block and recovered sinus rhythm with left bundle branch block (LBBB) at 8th postoperative day. LBBB occurred in another case. Their rhythm changes were fixed. The postoperative echocardiography at 7th postoperative day showed that the maximal/mean pressure gradients and aortic valve area were 20/13 mmHg and 1.0 cm2 in 19mm. 22.5/13mmHg and 1.6cm2 in 21mm, and 24/12mmHg and 1.7cm2 in 23mm valve, respectively.

**Conclusions**

Intuity valve implantation would be a reliable alternative to conventional aortic valve replacement, but various heart blocks caused by Intiuty valve need careful monitoring.

## O56 Direct true lumen cannulation technique in acute type - a aortic dissection

### Syed Shahabuddin, Shahid Ahmed Sami

#### Department of surgery, Aga Khan University Hospital, Karachi, Pakistan

##### **Correspondence:** Syed Shahabuddin

**Background**

Surgery for ascending aortic dissection has remained a challenge for cardiac surgeons. Various approaches have been used to achieve arterial flow including femoral, axillary and ascending aorta into false or true lumen. We share our experience of 10 cases where we adapted the technique of direct true lumen cannulation.

**Methods**

All patients undergoing surgical repair of Type-A Aortic Dissection by utilizing the technique of direct true lumen cannulation under vision from January 2012 to-date were reviewed.

**Surgical Strategy**

After entering the chest and full heparinization right atrial cannulation was performed. CO2 was insufflated. Patient was put in trendlenberg position, heart drained to achieve transient hypotension. Ascending aorta was opened in the middle and true lumen identified. The aortic cannula, already connected and primed, inserted into true lumen under direct vision and snugged with vascular tape and occlusion further ensured using IVC clamp. Cardiopulmonary bypass established and cooling started and the rest of the procedure completed in routine fashion.

**Results**

There were 10 patients. The age range was 25 to 60 years. Six patients underwent Aortic root replacement three of them also required graft to right coronary. In four patients aortic valve was resuspended and ascending aorta was replaced. One of them required AVR for Aortic regurgitation on TOE after coming off bypass. The mean CPB, Aortic cross clamp and circulatory arrest time were 237, 142 and 35minutes respectively. One patient died. Nine patients discharged home without any neurological complications or significant morbidity and followed up in the outpatient clinic. The follow up ranged from 2 days to 3 years.

**Conclusion**

Direct true lumen cannulation ensures antegrade perfusion to brain and other organs through true lumen. It is a viable alternative to other methods and the familiarity with the procedure makes it as good as other options.

## O57 Urgent thrombectomy of incidental diagnosed floating thrombi by multidetector computed tomography from the aortic arch

### Veneta Grigorova^1^, Georgi Vasilev^1^, Ivilin Todorov^1^, Galina Kirova^2^, Dimitar Nikolov^1^

#### ^1^Department of Cardiac Surgery, Acibadem Ciy Clinic Tokuda Hospital, Sofia, Bulgaria; ^2^Department of Medical Imaging, Acibadem Ciy Clinic Tokuda Hospital, Sofia, Bulgaria

##### **Correspondence:** Veneta Grigorova

**Objective**

Floating thrombus into the aortic arch is a very rare diagnosis, usually detected within a visceral, peripheral or cerebral embolisation. A friable floating thrombus in the high blood flow and pressure vessel, especially in the aortic arch might lead to serious complications as a life-threatening stroke, as well as peripheral embolisation. Optimal treatment and management remain controversial. We present and discuss our experience in managing floating thrombi in the aortic arch as well as in ascending aorta with surgical treatment.

**Methods**

Between June 2008 and March 2019 consecutive patients diagnosed with floating thrombus in the aortic arch and ascending aorta were reviewed and perioperative outcomes were assessed.

**Results**

Nine patients (3 male and 6 female) with thrombus formation in the aortic arch, diagnosed during multidetector computed tomography (MDCT) with a median age of 61 years (range, 51-69 years) were presented. All patients suffered peripheral embolic events. Eight patients underwent open urgent thrombectomy of the ascending aorta or aortic arch preventing new thromboembolic incidents. One of the patients died due to a stroke during the preparation for the operation. All eight patients had an uneventful postoperative recovery course. There was no recurrent thrombus or embolic event during follow-up.

**Conclusion**

Floating thrombus in the aortic arch is an unarguable source of systemic emboli, which should be treated as fast as possible. The surgical treatment remains the method of choice with very good clinical results. Nevertheless in inoperable or high risk patients the conservative anticoagulation therapy might be considered.

## O58 Experience of National Research Cardiac Surgery Center in surgical and endovascular treatment of cardiac diseases in peripartum women

### Selbi Meiramova^1^, T. Lesbekov^1^, Y. Kuatbayev^2^, Zh. Ashirov^2^, D. Shustov^1^, Y. Pya^1^

#### ^1^National Research Cardiac Surgery Center, Nur-Sultan, Kazakhstan; ^2^City Cardiology Center, Shymkent, Kazakhstan

##### **Correspondence:** Selbi Meiramova

**Background**

Pregnant women are a special category of patients in every field of medicine. Peculiarities of physiology and correspondingly pathophysiology of different type of disorders during pregnancy, limitations of some diagnostic and treatment procedures associated with increased risk for mother and fetus cause necessity in precise and strong evidence based approach. Pregnancy itself cause physiological changes in the background of which cardiac lesion may manifest itself or deteriorate.

**Objective**

Our aim is demonstration of results of endovascular and surgical treatment in our center.

Materials and methods: Since 2013 until May, 2019 there were 23 endovascular and surgical procedures in peripartum women.

9 (39%) – ECMOimplantation. 4 (17,4%) – mitral balloon valvuloplasty, 3 women (13%) had open surgeries on cardiopulmonary bypass (subaortic membrane excision, mitral and aortic valves replacement; right atrial thrombectomy, mitral valve replacement, tricuspid valve repair; mitral valve repair), 3(13%) – C-cection followed by open surgery on CPB (aortic valve replacement; mitral valve replacement; Davis’s procedure, brachiocephalic trunk replacement, tricuspid valve repair), 1 (4,3%) transcathethervalve in valve implantation, 1 (4,3%) – cava-filter placement, 2 (8,6%)–open surgeries after delivery or termination of pregnancy.

**Results**

Maternal mortality occurred in 6 cases (26%): 4 ECMO patients (2 of which had presented with Eisenmenger syndrome, 1 septic patient, 1 patient with primary pulmonary arterial hypertension), 1 patient was operated 32 days after C-cection (mitral valve replacement, tricuspid valve repair, ECMO) and died because sepsis, 1 patient was operated after termination of pregnancy due to frozen fetus(mitral valve replacement, tricuspid valve repair, ECMO), became septic and died.14 of women (61%) had viable fetus and gave birth naturally or by C-section.

**Conclusion**

Peripartum women with severe and symptomatic lesions can be treated surgically and/or with endovascular techniques in case of clear indications and highly experienced multidisciplinary team, because these patients remain category of high risk both for mother and fetus.

## 059 Rare case of a migration of arterial stent, placed in the iliac vein into the right pulmonary artery - late outcomes after surgical treatment

### Yankov Georgi, Anatoli Semkov, Mihail Plochev, Danail Petrov

#### Thoracic Surgery department, MHATPD “St. Sophia”, Medical University Sofia

##### **Correspondence:** Anatoli Semkov

Vascular stenting was introduced in 1969 by Dotter. The purpose of this procedure is to achieve normal blood flow and patency of venous vessel. Despite the benefits of this procedure, there are some serious complications such as rethrombosis, injury and migration of the stent.

We present the case of a 56 year old woman who was hospitalised in the Department of Thoracic Surgery of SHATPD “St. Sophia” with X-ray and CT data for foreign body looking like a vascular prosthesis in the interlobar part of the right pulmonary artery. Fibrobronohoscopy was done and it revealed to no pathological changes.

Four years ago in the Department of Vascular Surgery was implanted non-drugeluting arterial stent 8/39 through conventional inguinofemoral approach and venotomy of the common femoral vein. The procedure was done because of deep venous thrombosis of the right external iliac vein. In the past the patient also had an accident of pulmonary embolism which was treated conservatively.

The patient was operated in the Clinic of Thoracic Surgery via right sided posterolateral thoracotomy . Longitudinal arteriotomy of the interlobar part of the right pulmonary artery was performed and the stent was removed. Through neointimal proliferation and partial organized thrombus in the proximal part of the stent and in the beggining of 4th segmental artery in this area was performed thromboendarterectomy and the arteriotomy was sutured with 5/0 continuous suture. The postoperative course was uneventful and the patient was discharged on the fourth postoperative day. There is no data for complications at the last follow up – 01.05.2019.

Despite reports in the literature for the removal of migrated stents by mini-invasive interventional methods in our case this was not possible, because of the long period for which the foreign body was in the right pulmonary artery and the proximal partial occlusion of organized mural thrombus. The high risk of tissue damage by the rigid arterial stent via catheters was the main indication for open intervention and thromboendarterectomy of right pulmonary artery.

## 060 Long - term results of surgery in cases benign tracheal diseases - single institution study

### Yankov Georgi, Anatoli Semkov, Vladimir Stanoev, Mihail Plochev, Danail Petrov

#### Thoracic Surgery department, MHATPD “St. Sophia”, Medical University Sofia

##### **Correspondence:** Anatoli Semkov

**Aim**

To present our experience and evaluate results after surgery in patients with injury and postintubation stenoses of the trachea.

**Materials and methods**

A total of 85 surgical interventions on the trachea were performed for a period of 20 years (1.5.1999 – 30.04.2019) in 79 patients with mean age 39.4 years (15-77), 53 males and 26 females. In 80 patients the diagnosis was tracheal stenosis and rupture in 5 patients. Operations were 73 resections and reconstructions, 5 sutures and 4 resections of pediculated benign endotracheal tumors. In the past years 10 patients with small lacerations without displacement of the borders were treated conservatively.

**Results**

No perioperative death was observed. Major complications were observed in 5 patients: restenosis and reresections in 4 patients and reoperation due to prolaps of the proximal mucosa in 1. Minor complications were observed in 3 patients – insufficiency of the suture line with successful conservative treatment. The mean inhospital stay was 13.7 days (7-30).

**Conclusion**

Surgical approach in selected patients with tracheal is followed by excellent short and long term results.

## O61 Short term outcome of hand-sewn bovine pericardial conduits for RVOT reconstruction

### Ravi Agarwal, Santosh Wadile, Roy Varghese, Sivakumar K

#### The Madras Medical Mission, Chennai, Tamilnadu, India

##### **Correspondence:** Ravi Agarwal

**Background**

Right ventricle to pulmonary artery conduit is an essential part of surgical reconstruction for some forms of congenital heart disease. Ideal conduit should be easily available, cost effective and should have longevity. This study reviews our experience with implantation of hand sewn valved bovine pericardial conduits for right ventricular outflow tract reconstruction.

**Methods**

This is a retrospective analysis of patients who underwent surgical correction using a RV to PA conduit at our hospital. The conduits were hand-sewn using either gluteraldehyde treated bovine pericardium (St. Jude®) or decellularized bovine pericardium (Synkroscaff®). PTFE membrane or bovine pericardium was used to fashion a bicuspid valve in the conduit.

**Results**

35 patients underwent surgical correction with RV to PA conduit (21 males and 14 females). The median age was 1 year 11 months (range 1 month to 26 years) and median weight was 8 kg (range 2.8 to 51 kg). The diagnosis was TOF with pulmonary atresia or absent pulmonary valve in 23 patients; truncus arteriosus 7 patients, TGA with VSD & PS 4 patients and one patient had RV to PA conduit implanted as part of Ross procedure.

Follow up ranged from 1 month to 3 years; 7 patients required conduit related re-interventions (balloon dilatation and stenting). In 6 of these patients the conduit was prepared from decellularized pericardium while in 1 patient it was made of gluteraldehyde treated pericardium (p= 0.008). It was also noted that the conduit intervention rate was significantly higher if the conduit size was 14 mm or smaller.

**Conclusion**

Hand-sewn bovine pericardial conduits are cost effective and can be easily prepared on table if required. They provide effective palliation and have excellent short term results. The use of decellularized pericardium and smaller conduit size are risk factors for early conduit re-intervention.

## O62 Long-term survival after radical surgery for pulmonary large-cell neuroendocrine carcinoma

### Lili Oheda^1^, Georgy Yankov^1^, Mihail Plochev^1^, Emilia Naseva ^2^, Danail Petrov^1^

#### ^1^Thoracic Surgery Department, Medical University of Sofia, University Hospital for Active Treatment of Pulmonary Deseases “St. Sophia”, Sofia, Bulgaria; ^2^Faculty of Public Health Medical University of Sofia, Sofia, Bulgaria

##### **Correspondence:** Lili Oheda

**Aim**

To perform a retrospective analysis of postorerative outcomes in patients with large cell neuroendocrine lung carcinoma (LCNELC).

**Materials and methods**

A total of 29 patients were operated for LCNELC between 2000-2017. They were 3(10,34%) women and 26(89,66%) men, mean age 61,2 years. Radical surgery and systematic mediastinal lymph node dissection were performed in all cases. The operative volume included: pulmonectomy in 10 (34,48%) patients - intrapericardial (4), partial atrial resection (1) and chest wall resection (1); lobectomy in 17 (58,62%) patients, one intrapericardial; pyramidectomy in 1 (3,45%) patient and S1-S2 segmentectomy in 1 (3,45%) patient. The S1-S2 segmentectomy was performed 3 months after a radical resection of the solitary brain metastasis from the pulmonary tumor. All of the tumors expressed chromogranin, synaptophysin and NCAM in immune-histochemistry studies. pStage was as follows: stage IA- 2 (6.90%); stage IB- 2 (6.90%); stage IIA -4 (13.79%); stage IIB-10 (34.48%); stage IIIA - 7 (24.14%); stage IIIB- 3 (10,34) and stage IVA-1 (3.45%) patients. Adjuvant chemotherapy and/or radiotherapy were administered to all patients. They were followed either until their death or to the end of the study. The mean follow-up was 41 months. The survival was calculated by Kaplan-Meier method.

**Results**

One patient died in the early postoperative period of massive pulmonary thromboembolism. Two patients had postoperative bleeding, one of them was reoperated for definitive hemostasis. No serious complications were observed in the rest of the cases The median survival time was 30,133 months. Overall survival is at the 1th-year 70.7%; at the 3rd-year 44.9% and at 5th-year 39.3%. At the end of the study 15 (51.72%) patients died, 13 of them with disease progression and 2 due to another reason.

**Conclusion**

Radical surgery in LCNELC patients is a method of choice and even in the early stages it is recommended to be combined with adjuvant chemotherapy and/or radiotherapy due to tumor aggressiveness.

## O63 Comparison of the effect on endothelial dysfunction of single dose cardioplegia (Del Nido) and multi dose cardioplegia (St Thomas II)

### Erman Sureyya Kiris^1^, Eren Gunertem^2^, Uğur Aksu^3^, Hakkı Zafer Iscan^2^, Ertekin Utku Unal^2^

#### ^1^Hakkari Devlet Hastanesi Kalp Damar Cerrahisi, Hakkari, Turkey; ^2^TC Saglik Bakanligi Ankara Sehir Hastanesi Kalp Damar Cerrahisi, Ankara, Turkey; ^3^Istanbul Universitesi Fen Fakultesi Biyoloji Anabilim Dalı, Istanbul, Turkey

##### **Correspondence:** Erman Sureyya Kiris

**Aim**

Cardioplegic solutions play a key role on myocardial protection which is amajor issue in modern cardiac surgery. Different cardioplegic solutions have beengenerated throughout history of cardiac surgery. There are several clinical studies oncomparison of St. Thomas II cardioplegic solution -which has been used for decadesin our clinic- and Del Nido cardioplegic solution, which is also in use for a couple ofyears in our clinic. The aim of this study is to compare the difference of their effectson cardiac endotelial dysfunction and cardiac oxygen metabolism.

**Patients and Methods**

Patients who undergone mitral valve surgery were enrolledin two groups according to cardioplegic solution (ST n:33, DN n:30). Syndecan-1, malondialdehyde, ischemia modified albumin and lactate levels and blood gasanalyses are measured from the samples taken from coronary sinus at times of pre-ischemia (t0) and post-reperfusion (t1). Postoperative complications, mortality, intensive care unit and hospital stay were recorded.The study was approved by Ankara Numune Eğitim ve Araştırma Hastanesi‘s Ethics Board, approval number E-17-1389.

**Results**

Cross clamp and cardiopulmonary bypass times were similar in both groups(respectively p=0.186 and p=0.126). Total amount of cardioplejic volume wassignificantly higher in ST group (p<0.001). In blood gas analyses, DN group wasmore acidotic (p=0.036) and venous oxygen parameters were higher in DN group(PvO 2 , p=0.019, SvO 2 p=0.087) at the reperfusion period. Oxygen extraction ratio ofST group was increased earlier than DN group (respectively 30% and 21%, p=0.035). Syndecan-1 levels was decreased after reperfusion at both groups, howeverthe decrease was significant in ST group (p=0.016).

**Conclusion**

In our study, the only advantage of single dose cardioplegia was thelesser volume. On the other hand, normalization of oxygen consumption andendothelial dysfunction at reperfusion following cardioplegic arrest was more fasterat the ST group.

## O64 Use of incentive spirometry in adults following upper abdominal surgery to prevent postoperative pulmonary complications: a systematic review

### Kerrie A Sullivan^1,2^, Isabella F Churchill^1,2^, Waël C Hanna^1,2^

#### ^1^Health Research Methodology, McMaster University, Hamilton, Ontario, Canada; ^2^Department of Thoracic Surgery, St. Joseph’s Healthcare Hamilton, Hamilton, Ontario, Canada

##### **Correspondence:** Kerrie A Sullivan

**Background**

Following upper abdominal surgery (including cardiac and thoracic surgery), patients are at risk of experiencing postoperative pulmonary complications (PPC). Incentive spirometry is a rehabilitation therapy that is used to improve pulmonary expansion and maximal inhalation. The objective of this review is to determine if the use of postoperative incentive spirometry is more effective than other therapies at reducing the risk of PPC, mortality and hospital length of stay in adults following such surgeries.

**Methods**

We searched the Cochrane Central Register of Controlled Trials, EMBASE, MEDLINE, CINAHL and Web of Science for randomized controlled trials comparing incentive spirometry to a control group of another rehabilitation therapy or no intervention. Two review authors independently assessed risk of bias and abstracted data from the included studies. Additional information was sought from trialist when data were missing or not reported. Data were pooled for meta-analysis and GRADE was used to assess the quality of evidence.

**Results**

Data on the incidence of PPC were pooled for meta-analysis from 20 studies (3059 participants). The risk ratio was 0.95 (95% CI: 0.75-1.22), which translated to an absolute effect estimate of 1 fewer patient per 100 (95% CI: 4 fewer to 1 more). Eight studies reported mortality (2132 participants), in which the pooled analysis had a risk ratio of 0.51 (95% CI: 0.25-1.06) and an absolute effect estimate of 19 fewer deaths per 1000 (95% CI: 28 fewer to 2 more). Lastly, 10 studies reported length of hospital stay (2229 participants). The pooled analysis showed a mean difference of 0.40 fewer days (95% CI: 1.02 fewer to 0.22 more).

**Conclusions**

Incentive spirometry likely results in little to no reduction in the number of patients with PPC, mortality or hospital length of stay, following upper abdominal surgery in adult patients.

## O65 Mini-thoracotomy approach with central cannulation for repair of ventricular septal defects – a better alternative

### Anil Sharma, Sunil Dixit, Mohit Sharma, Jaikishan Suthar

#### Department of cardiothoracic and vascular surgery, S. M. S. Medical College, Jaipur, Rajasthan, India

##### **Correspondence:** Sunil Dixit

**Background**

At present thoracotomy with femoro-femoral bypass is established approach for minimally invasive open heart surgeries but thoracotomy with conventional cannulation is yet to be established. After doing many valvular surgeries and ASD closures at our center we performed 34 cases of VSD closure by Single Incision Right Anterior mini-thoracotomy approach with central cannulation (like sternotomy) without femoro-femoral by-pass. This study shows our results and experience to determine the feasibility and safety of this approach for congenital heart disease.

**Aim and objective**

Aim of our study was to evaluate the outcomes of VSD repair via Single Incision Right Anterior Mini-Thoracotomy (SIRAMT) with central cannulation.

Exclusion criteria: Patients with right ventricular outflow tract obstruction, Ischemic VSDs , atrioventricular canal defects were excluded.

**Methods**

This is a retrospective observational descriptive type of study. Fifty four patients underwent VSD repair via right anterior mini-thoracotomy with age ranges from 3 to 22 years. Data were collected from May 2017 to Jan 2019. Demographics, operative techniques and post operative morbidity and mortality along with follow up were recorded. CPB was instituted from the same thoracotomy incision.

Results: Successful repair of the defects was achieved in all the patients. No patients died or converted to median sternotomy. Average duration of cardiopulmonary bypass (CPB) was 58.22 ± 15.30 min (range, 48–78 min) and aortic cross-clamp time was 41.51 ± 12.03 min (range, 26–56 min). The average postoperative ICU stay was 1.63 ± 1.02 days (range, 1-3 days) and hospital stay was 4.62 ± 1.62 days (range, 4–7 days). There were no postoperative complications related to the operative procedure. Early results and midterm results in follow-up were found good.

**Conclusion**

Our experiences demonstrate that minimally invasive cardiac surgical technique via right anterior mini-thoracotomy can be a safe and better alternative for the repair of VSDs like valvular and ASD surgeries. In this technique no groin incision and related complications.

**Keywords**

Ventricular septal defect, Central Cannulation, Right anterior mini thoracotomy.

## O66 Video-assisted thoracoscopic surgery for bronchogenic cysts in early childhood

### Yanko Pahnev^1^, Hristo Shivachev^1^, Zdravka Antonova^1^, Nikola Kartulev^1^, Nia Atanasova^1^, Vanya Strahinova^2^, Daniela Antonova^2^

#### ^1^Pediatric Thoracic Surgery Department, Clinic for Pediatric Surgery, UMHATEM ‘N.I.Pirogov’, Sofia, Bulgaria; ^2^Radiology Department, UMHATEM ‘N.I.Pirogov’, Sofia, Bulgaria

**Introduction**

Bronchogenic cysts (BC) represent a rare anomaly in the development of the ventral part of the foregut and budding of the lung between 4-6 weeks of gestation (7-15% of all foregut-abnormalities, second in this group after enterogenic cysts [1]). The incidence ranges from 1/45,000 to 1/68,000 live births and have a good prognosis. [2] The clinical presentation includes various non-specific symptoms according to their localization – compression, inflammatory complications, pain, pneumothorax, cough, haemoptysis etc. The differential diagnosis includes a wide range of congenital cystic and tumor masses in the chest, mediastinum and abdomen, for instance: CPAM, pulmonary sequestration, pericardial cyst, cystic hygroma, lymphangioma, mesenteric cyst, anterior or posterior meningocele, oesophageal duplication, thymic cyst, thyroid colloidal cyst, pancreatic pseudocyst, lung pseudotumor and more. Operative treatment is mandatory after the disease is diagnosed. The main operative methods are devided in 2 major groups - open (conventional) surgery and mini-invasive surgery (thoracoscopy / laparoscopy).

**Material and methods**

We present 3 clinical cases of children with mediastinal bronchogenic cysts treated by VATS, aged 1 to 3 years old. Chest X-ray and thoracic CT scan were used for imaging diagnostics. An endobronchial balloon-catheter is used for the purpose of single-lung ventilation. 4-ports VATS, 30/5 mm optics were used.

**Conclusions**

The use of video-assisted thoracoscopic surgery (VATS) causes minor surgical trauma, reduced anesthetic risk and has a significantly shorter recovery period in comparison with the open surgery. It is also presented with better cosmetic results.

## O67 The efficacy of box lesion+ procedure in patients with atrial fibrillation: Five-year follow-up results

### Oleg Sapelnikov, Dmitrii Cherkashin, Igor Grishin, Olga Nikolaeva, Darin Ardus, Andrey Shiryaev, Renat Akchurin

#### Russian Cardiology Research Center, Moscow, Russia

##### **Correspondence:** Oleg Sapelnikov

**Background**

Different modifications of MAZE procedure nowadays lead to significant improvement in surgical treatment of atrial fibrillation (AF). Our study compares two approaches of Cryo-MAZE procedure presenting 5-year follow-up results.

**Methods**

Fifty-two (52) patients with atrial fibrillation (AF) were included in the study. Twenty (20) patients had concomitant coronary bypass grafting and thirty-two (32) patients had mitral valve replacement. Mean age was 60,1±10,25 years, prevalence of men was admitted (59,6%). Mean duration of AF was 4.75±5.44 and 7.07±8.14 years. We divided patients into 2 groups according to endocardial Cryo-MAZE procedure type – the first group underwent pulmonary vein (PV) isolation with ablating line at left atrium roof and ablation of mitral isthmus; patients in the second group had isolation of posterior wall of the left atrium (LA) and perimitral area (Box-lesion+ group). The groups were comparable including the majority of demographic

**Characteristics**

Ablation was performed with the Cardioblate Cryoflex Cryoablation System (Medtronic).

Results: Cryo-MAZE procedure directly lasted 18±1,7 min in the first group and 20±2,1 min in the second group (p – 0,398); the whole operation time was 192±24 min and 199±19 min (p – 0,435) and artificial circulation time was 103±12 min and 104±10 min (p –0,547). By the third year of follow-up the efficacy of ablation in the second group significantly exceeded results of the first group 79,2% versus 41,6 (p –0,013). In 5 –year follow-up we received that the group of box lesion + had no AF recurrences in 72,8% versus 39,4 % (p – 0,038).

**Conclusion**

Box-lesion+ procedure demonstrated encouraging results in AF treatment during the whole follow-up period.

## O68 Aortic valve leaflet reconstruction with unequal sinuses of Valsalva guided by a virtual reality image

### Takeo Tedoriya, Ryoi Okano, Tadamasa Miyauchi, Yuko Galatea, Masaomi Fukuzumi

#### Cardiovascular Center, Ageo Central General Hospital, Ageo, Japan

##### **Correspondence:** Takeo Tedoriya

**Objectives**

We developed a novel aortic valve leaflet reconstruction (AVLR) technique using three uniform leaflets created from autologous pericardium, sized according to the diameter of the sinotubular junction (STJ). Using a new three-dimensional (3D) image workstation, the anatomical structure of the aortic root was evaluated preoperatively in order to decide additional steps for adjustment of the aortic annulus following our basic leaflet procedures. We evaluated surgical outcomes and three-year follow-up.

**Method**

The technique uses original leaflet templates, selected according to the diameter of the sinotubular junction (STJ), to tailor leaflets from autologous pericardium treated with 0.6% glutaraldehyde solution. Before attaching these leaflets, the anatomical structure of the aortic root was assessed using a virtual reality image, which was obtained by contrast enhanced ECG-triggered cariac computed tomography (CT). Axial images using 264-row CT (slice thickness, 0.625 mm) were obtained during mid-to-end diastole. Then, subtracted volume-rendered CT data were converted to stereolithography files for Visalius3D on a C-Station workstation.

From 2015, we have performed AVLR in 24 patients (AS=10, AR=14). There were 4 bicuspid cases. In 5 patients, we performed additional annular adjustment procedures (nadir plication and/or creation of new commissaries).

**Results**

There is no operative mortality. One redo AVR was done due to perforation of leaflet. Echocardiographic evaluation revealed no AR more than mild, and mean AVPG was 8.7mmHg after the surgery, 7.0mmHg in 1 year, 8.8mmHg in 2 years.

**Conclusion**

The AVLR technique described herein can provide a simple and reproducible procedure that allows anatomical physiologic correction of the aortic valve for various types of aortic valve disease. Notably, preoperative evaluation using a VR image of the aortic root has provided valuable information for the adjustment of the neo-commissure and nadir in our AVLR surgery.

## O69 64 year old male with left main coronary dissection and aortic root perforation with tamponade at the time of elective cardiac catheterization

### Jeko Madjarov, Francis Robicsek

#### Carolinas Medical Center- Atrium Health, Charlotte, NC

##### **Correspondence:** Jeko Madjarov

The patient is a 64 y/o male with PMH CAD, dyslipidemia, anxiety, depression, GERD and COPD (heavy smoking). He presented for elective cardiac cath. Lcx and LAD were stented with DES. Left main was attempted without success with no flow post PCI. Left main coronary dissection with acute cardiogenic shock from coronary malperfusion as well as pericardial tamponade secondary to focal aortic dissection with aortic root perforation. The patient was coded for 45 minutes. Impella CP (4 liters) was introduced via the right CFA in the cath. lab. The patient was then taken to the OR where he underwent salvage/emergent mediastinal exploration, aortic exploration with repair of aortic wall perforation and CABG with greater saphenous vein x2, SVG to LAD, SVG to OM; Impella 5.0 LD placement via side branch (10 mm Vascutek) to the distal ascending aorta; Removal of RCFA Impella CP ; VA ECMO cannulation via RCFA and RCFV; open chest with wound VAC. Starting lactate was 9.0.

The patient suffered periop. bleeding with acute blood loss anemia and coagulopathy; Multifactorial shock: cardiogenic, circulatory and later septic; Post-op arrhythmias (VF and VT); acute respiratory failure; serratia pneumonia with associated septic shock; shock liver; AKI, critical illness weakness.

The patient progressed in ICU and was ultimately transferred to rehab and subsequently discharged home.

**Conclusion/Discussion**

The simultaneous use of Impella 5.0 LD and VA ECMO as a “salvage strategy” in the case of acute cardiogenic shock with multiorgan failure.

## O71 Retrograde cerebral perfusion as an appropriate method of cerebral protection during surgical correction of ascending aorta and aortic arch aneurysms

### Vitalii Kravchenko, Ivan Kravchenko, Iryna Osadovskaya, Diana Gorban, Djachenko Valerii, Vasyl Lasoryshynets

#### Amosov National Institute of Cardiovascular Surgery, Kyiv, Ukraine

##### **Correspondence:** Diana Gorban

**Introduction**

Antegrade and retrograde cerebral perfusion (ACP, RCP) remain a topic for discussion in ascending and aortic arch aneurysms surgery.

**Aim**

To select the most efficient technology for RCP based on one-center experience.

**Materials and methods**

377 patients were operated during 1994-2017 due to ascending and aortic arch aneurysms, 300 of them were males (79.6%), 77 – females (20.4%). Mean age was 54,5 ± 9,8 years. Among them, 319 patients (84.6%) were diagnosed with acute or subacute dissection, 24 (6.4%) – chronic; 34 pts (10.3%) had no dissection. Possible predisposing factors/illnesses were: arterial hypertension & atherosclerosis (248 pts – 65.9%), Marfan syndrome (41 pts -10,9%), congenital bicuspid aortic valve (39 pts –10.3%), cystomedionecrosis (25 pts – 6,6%), syphilis (14 pts – 3.7%), Takayasu’s arteritis (3 pts – 0.8%), falling from a height (2 pts – 0.5%), unknown reasons (5 pts – 1.3%).

In most cases operations were performed with deep hypothermia (DH) and RCP, held via SVC, however, in 4 cases ACP was used. Femoral artery was utilized for arterial cannulation (366 pts –97.1%). Supracoronary grafting of ascending aorta with hemiarch or arch was done in 260/7 cases correspondingly (68.9%), the Bentall operation with hemiarch/arch – 83/8 cases (22%), arch grafting – 15 (4%), Wheat operation with aortic arch grafting – 7 (1.9%), aortic arch plasty – 4 (1.1%), hybrid repair with Elephant Trunk procedure – 8 (2.1%). In 23 cases (6.1%) patients had also CABG.

**Results**

Depending on hypothermia level and perfusion parameters we’ve divided all our data in three groups/periods. Group I (1994-2001) included 25 operations with DH level 16-18°C, perfusion flow 500-750 ml/min/m², SVC pressure 15-25 mm Hg and mortality rate 28% (7pts). Brain injury was a cause of death for 2 pts. Group II (2002-2007) included 63 operations, DH 12,5-14°C, perfusion flow 250-500 ml/min/m², SVC pressure 10-12 mm Hg, mortality rate 17,4% (11 pts). 5 pts developed lung complications, 3 of them died. There was 1 pts (1.6%) with lethal brain injury. Group III (2008-2017) included 289 operations, DH 18-20°C, perfusion flow 250-500 ml/min/m², SVC pressure 10-12 mm Hg. In 31 cases circulatory arrest was used. 30-day mortality rate composed 4.5% (13pts). Overall 30-day mortality composed 8.2% (31 pts).

**Conclusions**

RCP with deep hypothermia (18-20°C), SVC pressure 10-12 mmHg and blood flow rate 250-500 ml/min/m² with continuous perfusion via femoral artery is a safe method of brain protection during operations on ascending and aortic arch aneurysms.

## O72 Pulmonary Resections For In Situ And Minimally Invasive Adenocarcinomata In The Elderly: Prognostic Factors For Mortality And Recurrence

### Walid Mohamed, Mohammed Chowdhry

#### Department of Thoracic Surgery, University Hospitals of Leicester, Leicester, UK

##### **Correspondence:** Walid Mohamed

**Objectives**

Adenocarcinoma in situ (AIS) and minimally invasive adenocarcinoma (MIA) have an expected 5-year disease-free survival of near-100%. However, there is a paucity of data regarding prognostic indicators and outcomes of pulmonary resections for pure AIS or MIA lesions, especially in elderly patients.

**Methods**

All patients who underwent anatomical resection (AR) or wedge resection (WR) for solitary pulmonary nodules (SPNs) with a histopathological diagnosis of adenocarcinoma ≤ 3 cm in size from 2012-2016 were assessed. Patients were divided into 3 cohorts: Group 1 had patients at least 70-years-old with pure AIS or MIA lesions, group 2 had patients less than 70-years-old with pure AIS or MIA, and group 3 had patients at least 70-years-old with invasive adenocarcinoma without local or distant metastasis (IA). Preoperative risk factors, histopathology results and resection type were examined to determine prognostic significance for in-hospital and 3-year mortality and morbidity.

**Results**

77 patients were included: Group 1 (n=22; mean age= 75.4 ± 4.5 years; 4 males; 8 WR), group 2 (n=18; mean age= 56.6 ± 9.6 years; 6 males; 13 WR) and group 3 (n=37, mean age= 74.8 ± 3.7 years; 13 males; 6 WR). Patients in all 3 groups showed no in-hospital mortality but in-hospital cardiopulmonary complications were significantly higher in group 1 (n=4) (18.2%) than group 2 (n=2) (11.1%) (p<0.0001). Three-year recurrence was significantly higher in group 1 (n=5) (23%) than group 2 (n=3) (17%) (p<0.0001). Group 3 showed a higher complication rate (40.5%) and 3-year recurrence (35.1%). Three-year mortality in group 1 (9.5%) and group 3 (5.5%) was lower than group 2 (46%), while disease-specific mortality (DSM) was 4.5% in group 1, 0% in group 2, and 27% in group 3 (p<0.0001 for all).

**Conclusion**

Pure AIS or MIA lesions in elderly patients have a relatively high recurrence rate but low DSM compared to similar patients with IA, and a higher in-hospital morbidity compared to the same pathology in younger patients. Risk-benefit analysis is therefore essential in elderly patients with SPNs ≤ 3 cm suspicious of AIS, MIA or IA to determine the necessity and type of lung resection.

## O73 Esophagectomy for Esophageal adenocarcinoma and postoperative complications in kidney transplant Patient

### Yankov Georgi^1^, Vladimir Stanoev^1^, Anatoli Semkov^1^, Rossen Petkov^2^, Danail Petrov^1^

#### ^1^Surgery Department, “Saint Sophia” MHATPD, Medical University, Sofia, Bulgaria; ^2^First Clinic for Treatment of Non-specific Lung Diseases, “Saint Sophia” MHATPD, Medical University, Sofia, Bulgaria

##### **Correspondence:** Anatoli Semkov

**Objectives**

The overall risk of cancer for immunosuppression is increased between two and threefold compared with that of the general population due to various reasons, and probably include many factors such as the types, duration, and burden of immunosuppression. Cancers in transplant recipients were proven to be more aggressive than those in patients without transplants. Esophageal surgery in kidney transplant recipients is very rare.

**Methods**

A 33-year-old man was listed for kidney transplantation for end-stage renal failure due to Bardet-Biedl syndrome. Before surgery he underwent long term hemodialysis, during which infection with HCV and CMV was established. Postoperatively immunosuppressive therapy with Cyclosporin A was administered. Three years after the transplantation the patient was admitted to our department with chest pain, difficulty in swallowing and serious body weight reduction. There were no pathological findings on CT and abdominal ultrasound, but fiberoptic esophagogastroscopy revealed an ulcer at about 25 cm from the dentition. The histopathological examination established a low differentiated adenocarcinoma.

**Results**

A subtotal esophagectomy with a gastroesophagoplasty a modo Ivor-Lewis-МcKeown and a pyloroplasty were performed with uneventful postoperative period (pStageI). Seven months later the patient was hospitalized again for spontaneous bilateral hemorrhagic pleural effusions. Right thoracoscopy for a coagulated right-sided hemothorax and a left pleural drainage was carried out. Six years after esophagectomy a coronary stent was placed due to myocardial infarction. During the last follow up (postoperative year 8th) the patient being on immunosuppressant therapy (Certican 0.75-1mg and Deltacort 0.5mg) displays a good condition without complaints.

**Conclusions**

Here we present an extremely rare case of esophagectomy for early adenocarcinoma in a kidney transplant recipient with excellent long-term result. Late spontaneous pleural hemorrhage due to immunosuppressive therapy is extremely rare, and it was successfully treated. There are no reports in the literature relating to similar patients. We believe that routine cancer screening is recommended for all transplant recipients.

**Keywords**

Esophageal cancer, Esophagectomy, End-stage renal failure, Kidney transplantation, Bardet-Biedl syndrome.

The patient provided written consent to the publication of clinical or personal details.

## O74 Lymph Node Metastases of Esophageal Cancer and Blood Cell Circuit

### Oleg Kshivets

#### Surgery Department, Roshal Hospital, Roshal, Moscow, Russia

**Objective**

Significance of blood cell circuit in terms of detection of esophageal cancer (EC) patients (ECP) with lymph node metastases was investigated.

**Methods**

We analyzed data of 543 consecutive ECP (age=56.4±8.8 years; tumor size=6±3.5 cm) radically operated (R0) and monitored in 1975-2019 (m=405, f=138; esophagogastrectomies (EG) Garlock=280, EG Lewis=263, combined EG with resection of pancreas, liver, diaphragm, aorta, VCS, colon transversum, lung, trachea, pericardium, splenectomy=151; adenocarcinoma=308, squamous=225, mix=10; T1=126, T2=114, T3=178, T4=125; N0=275, N1=69, N2=199; G1=157, G2=139, G3=247; early EC=107, invasive=436). Variables selected for study were input levels of blood cell circuit, sex, age, TNMG. Differences between groups were evaluated using discriminant analysis, clustering, nonlinear estimation, structural equation modeling, Monte Carlo, bootstrap simulation and neural networks computing.

**Results**

It was revealed that separation of ECP with lymph node metastases (n=268) from ECP without metastases (n=275) significantly depended on: erythrocytes (abs, total), leucocytes (total), segmented neutrophils (total), eosinophils (%, abs, total), monocytes (%, abs, total), thrombocytes (abs), coagulation time, protein, residual nitrogen, cell ratio factors (CRF) (ratio between cancer cells- CC and blood cells subpopulations), T, G, tumor size, tumor growth, histology (P=0.045-0.000). Neural networks computing, genetic algorithm selection and bootstrap simulation revealed relationships of lymph node metastases and CRF: healthy cells/CC (rank=1), erythrocytes/CC (2), monocytes/CC (3), lymphocytes/CC (4), thrombocytes/CC (5), segmented neutrophils/CC (6), eosinophils/CC (7), leucocytes/CC (8), stick neutrophils/CC (9). Correct classification N0—N12 was 100% by neural networks computing (area under ROC curve=1.0; error=0.0).

**Conclusion**

Lymph node metastases significantly depended on blood cell circuit.

## O75 Surgical ventricular restoration in patients with left ventricular aneurisms

### Boyan Baev, Dimitar Kyuchukov, Tihomir Hristov, Dimitar Petkov, Rumen Iliev, Gencho Nachev

#### Department of Cardiovascular Surgery, University Hospital “St. Ekaterina”, Sofia, Bulgaria

##### **Correspondence:** Dimitar Kyuchukov

**Objective**

Endoventricular patch reconstruction of the left ventricle is increasingly performed in patients with LV aneurisms . We report our 15 year results comparing conventional linear closure to endoventricular reconstruction (Dor procedure).

**Methods**

Between January 2003 and January 2018, 467 consecutive patients underwent SVR, with mean age of 61.7 and male gender in 365 . The patients are divided in two groups: Group I -214 pts with Dor procedure , Group II –243 pts with linear resection. The patients in both groups have similar pre- and intra-operative parameters with angina in 369 pts and congestive heart failure in 204 pts.. Euroscore averaged 10 (6,8- 57,9). Concomitant to SVR, mitral valve reconstructions, VSD closure, CABG and trombectomy have been performed in 84 (18.7%), 8 (1.78%), 444 (99.3%) and 135 (30,2%) patients, respectively.

**Results**

Mean follow-up was 67 ± 41 months with actuarial survival at ten years of 54% . The operative mortality was 9,6%(44 pts) for the whole group and 14,2%(12pts) in the group with concomitant MVR . In both groups EF improved from 30% to 36,6%, NYHA-class was reduced from 3,3 to 1,5 , LVEDV was reduced by 42 ± 43 ml and LVESV- by 40 ± 47 ml post-operatively. No difference was found between the Dor procedure and linear resection in terms of mortality, postoperative NYHA class or LV volume reduction.

**Conclusions**

SVR shows good results for the treatment of patients with LVA. Additional mitral valve procedure increase significantly the operative risk. The type of surgical reconstruction had no influence on mortality and survival. We recommend the endoventricular pach reconstruction in large aneurisms with septal involvement.

## 076 Endovascular treatment of chronic total occlusions of subclavian artery

### Chavdar Bachvarov, Georgi Todorov, Veselin Petrov, Emil Jordanov

#### Department vascular surgery and angiology, Medical University, Varna, Bulgaria

##### **Correspondence:** Veselin Petrov

**Aim**

We present one centre experience in recanalisation and stenting of chronic occlusions of subclavian artery.

**Material and method**

Since January 2010 till june 2019 16 patients were managed by endovascular treatment. They suffered chronic occlusions of the subclavian artery. Preceding doppler sonography and CT angiography were performed in all patients. Indications for recanalisation was subclavian steal syndrome, pain during movement of the affected extremity, or symptoms of coronary ischamia in patients with elective or former aortocoronary bypass.

**Result**

Successful recanalisation was achieved in 14 patients, all with occlusions of the left subclavian artery.

In two patients repeated PTA with drug eluting balloon was performed because of presence of critical restenosis.

**Conclusion**

Endovascular treatment of chronic occlusions of subclavian arteries is effective mini-invasive procedure with low risks and good long term results.

## O78 Outcomes of primary complete repair of common arterial trunk: a 25 year experience

### Ventsislav Boshnakov^1^, Ivaylo Mitev^1^, George Konstantinov^1^, Plamen Mitev^1^, Dimitar Pechilkov^2^, Anna Kaneva^3^, Stojan Lazarov^1^

#### ^1^Department of Congenital Heart Surgery, National Heart Hospital, Sofia, Bulgaria; ^2^Pediatric Cardiac Intensive Care Unit, National Heart Hospital, Sofia, Bulgaria; ^3^Department of Pediatric Cardiology, National Heart Hospital, Sofia, Bulgaria

**Background**

During the last decades, neonatal primary repair has progressively evolved as the procedure of choice in patients with common arterial trunk (CAT). We sought to determine the outcomes after correction of CAT in all patients operated in our institution over 25 years.

**Methods**

Between 1993 and 2018, 36 patients underwent repair of CAT. Mean age was 3.5 months (6 days - 6 years). According to Van Praagh’s classification 22 patients were type A1, 11 patients were type A2, 2 patients were type A3 and 1 patient type A4. Repair with reconstruction of the right ventricular to pulmonary artery continuity was performed using a valved conduit in 34 patients, 1 patient was repaired with valveless conduit and 1 with direct anastomosis. Preoperative truncal valve regurgitation was seen in 30% of the patients, and concomitant congenital malformations had 21%. Survivors were repeatedly examined echocardiographically or catheterised for assessment of residual heart lesions.

**Results**

The early mortality is 19%. There are 4 late deaths, mainly due to septic complications. Reoperation for right ventricular outflow tract obstruction and truncal valve regurgitation was necessary in 14 patients (39%), 3 of them had subsequent second reoperation. Mean freedom from reoperation interval is 6.5 years (1.2-10.5 years). In 5 patients (14%) percutaneous valve interventions were necessary. Recent clinical examination finds all patients in good functional outcome, 1 patient with moderate valve insufficency.

**Conclusions**

Neonatal common arterial trunk repair remains a challenging operation with high morbidity and mortality despite advances in the last decades. Numerous reinterventions for conduit obstruction, pulmonary branch stenosis and truncal valve insufficiency are often required.

## O79 Risk factors and prevention of wound infection after cardiac surgery in Department of Cardiac Surgery, University Hospital “St. Marina”, Varna, Bulgaria

### Pavlin Manoilov^1^, Plamen Panayotov^1^, Veselin Hadjiev^2^

#### ^1^Department of Cardiac Surgery, University Hospital “St. Marina”, Varna, Bulgaria; ^2^University of Economics, Varna, Bulgaria

##### **Correspondence:** Pavlin Manoilov

**Key words**

cardiac surgery, wound infection, risk factors.

**Background**

Chest wound infections are a life threatening, time and money consuming complication of open-heart surgery with median sternotomy.

**Objective**

Characterization of the incidence and spread of wound infections at the Department of Cardiac Surgery, St. Marina University Hospital, Varna, identification of the risk factors, analysis of the results of the introduction of a Protocol for the Prevention of Wound Complications (PWC) after cardiac surgery, focused on reduction of the incidence of postoperative wound infections.

**Materials and methods**

Retrospective study of 1354 consecutive patients operated on by median sternotomy between 2011–2013 analyzing potential risk factors for postoperative wound infection. Prospectively collected data of 505 patients operated on between January - December 2014 were analyzed regarding risk factors and results after the Protocol for the PWC was applied. Data about wound infection after cardiac surgery were collected for the period 2011 –18.

**Results**

We found that the relative incidence of wound infection prior to the introduction of the Protocol for the PWC was higher than the incidence of wound infections after its introduction (P- 0,0007). Significant risk factors for developing wound infection after cardiac surgery in our Department include: diabetes mellitus (P-0,000), BMI > 25 (P-0,001), duration/h of mechanical lungs ventilation in the ICU (P-0,030, P-0.0141), chronic congestive heart failure (P-0.0499) and low levels of albumin (P-0.310).

**Conclusions**

Recognizing the risk factors for wound infection and preventing wound complications are key to limit the incidence and spread of wound infections. Strict adherence to a Protocol for the PWC leads to a reduction in wound complications. Weight reduction and strict glycemic control would reduce the risk of wound complications.

## O80 Surgical Resection of Pulmonary Lesion in Patients with Known Primary Cancer Site – Not Only Metastases, but Also Second Primaries and Benign Tumors

### George Kalaydjiev, Maria Yancova, Petko Tzonev, Michail Shindov

#### Clinic of Thoracic Surgery, University Specialized Oncological Hospital, Sofia, Bulgaria

##### **Correspondence:** George Kalaydjiev

**Background**

In lack of Evidence Based Guidelines for pulmonary metastasectomy, we try to present potential additional reasons in decision making processes for surgical resection of a new pulmonary lesion in cancer patients with known primary site (KPS).

**Materials and Methods**

We analyzed retrospectively the files of 303 patients with KPS for 10 years period (2009-2018). Nine of them had two previously treated KPSs. They were referred to our clinic for diagnosis and treatment of new pulmonary lesions. In a group of 234 pts fiberoptic bronchoscopy or transthoracic needle biopsy were performed and pulmonary metastases were proven in 69 pts. In other 79 pts. a second primary (lung) cancer was histologically diagnosed. In 86 pts. morphological diagnosis was not reached. All these patients were not eligible for surgery, due to different medical conditions. Surgical treatment was performed in 69 pts. – 48 with lung metastases, 12 with second primary (lung) cancer and 9 with benign lesions. In this communication we focus on the last two groups.

**Results**

No operative mortality and major surgical complications were registered. We noted 17,4% second primary (lung) cancer and 13% benign tumors in the operated group. At the time of surgery, a frozen section evaluation was performed by two pathologists. Thorough immunohistochemical examination was performed subsequently and comparison with histological specimens from the operated KPS to prove the difference between the KPS and the pulmonary lesion.

**Conclusions**

In cancer patients with known (treated) primary site and new pulmonary lesion a multidisciplinary approach is necessary in decision making for surgery. The potential existence of second primary or benign lesion must be kept in mind during this decision. Such a possibility is an additional reason for more aggressive surgical approach.

## O81 Early outcome, mortality and major morbidity after lung cancer surgery for primary carcinoma

### Fadil Gradica^1^, L. Lisha^1^, Dh. Argjiri^1^, A. Cani^1^, F. Kokici^1^, I.Skenduli^1^, D. Xhemalaj^1^, I.Avdiu^2^, H. Nino^2^, A.Pere^2^, L. Zhegu^1^, A. Menzelxhiu^1^, A .Kalenja^1^

#### ^1^University Hospital , “Shefqet Ndroqi” Tirana, Albania; ^2^QSUT “Mother Theresa Oncology Service”

**Background**

Radical surgical resection of lung cancer with or without adjuvant treatment is still a prerequisite for cure. Advances in operative and postoperative care led to a decline in complications and mortality rates during the last decades. In spite of different additional modes of treatment, survival is still poor.

**Methods**

We analyzed 968 patients who underwent lung resection for bronchial carcinoma, with non-small cell lung cancer during a 12-year period (January 2004-December 2017). Postoperative events studied were divided into major and minor complications or death during the first 30 days after surgery.

**Results**

Of 968 patient, 690 (70.5%) were male and 278 (28.7%) female. Mean age 65.5±9.4 years (range 15 - 87 years). Lobectomy was the most used surgical modality in 566 (58.5%) patients, meanwhile pneumonectomy was performed in 112 (11.6%) of patients. Minor complications during surgery occurred in 45 (11.7%) of patients. Continuous air leakage was the most complication after surgery in 25.3%, followed by lung atelectasis in 21.3% and cardiovascular complications in 17%. Of the life threatening complications respiratory failure was the most events in 20.0% of patients, followed by broncho-pleural fistula in 18.7% and pulmonary edema in 15% of patients. The 30 day mortality rate was 3.8% (37) patients, 1.2% after single lobectomy and 13.4 % after pneumonectomy.

**Conclusion**

Our results show low mortality and morbidity after lung cancer surgery. However, patients with reduced lung capacity, older age and those undergoing pneumonectomy should be treated with great care.

**Key words**

outcome, complications, lung cancer, thoracic surgery.

**References**

1. Pastorino U, Yang XN, Massimo F, et al. Long-term survival after salvage surgery for invasive thymoma with intracardiac extension. Tumori 2008;94:772-6.

2. Solaini L, Prusciano F, Bagioni P, et al. Long-term results of video-assisted thoracic surgery lobectomy for stage I non-small cell lung cancer: a single-centre study of 104 cases. Interact Cardiovasc Thorac Surg 2004;3:57-62.

3. Imperatori A, Mariscalo G, Riganti G, et al. Atrial fibrillation after pulmonary lobectomy for lung cancer affects long-term survival in a prospective single-center study. J Cardiothorac Surg 2012;7:4.

4. Daly BD, Fernando HC, Ketchedjian A, et al. Pneumonectomy after high-dose radiation and concurrent chemotherapy for nonsmall cell lung cancer. Ann Thorac Surg 2006;82:227-31.

5. Inoue M, Okumura M, Minami M, et al. Cardiopulmonary co-morbidity: a critical negative prognostic predictor for pulmonary resection following preoperative chemotherapy and / or radiation therapy in lung cancer patients. Gen Thorac Cardiovasc Surg 2007;55:315-21

## O82 Diabetes mellitus and abdominal aortic aneurysm: systematic review and 10 –year retrospective analysis

### Margaret Dimova, Nadelin Nikolov, Bistra Boneva, Detelina Lukanova, Mario Stankev, Stefan Stanev

#### National Heart Hospital, Sofia, Bulgaria

##### **Correspondence:** Margaret Dimova

**Background**

According to a number of epidemiological studies, diabetes mellitus is associated with a reduced risk of abdominal aortic aneurysm, but there is no evidence from prospective studies. We conducted a systematic review of prospective studies in search for a correlation between diabetes mellitus and abdominal aortic aneurysm. The second part of our study is a retrospective analysis for a 10-year period involving patients, treated in National Heart Hospital.

**Methods**

We summarized several prospective studies that report the relative risk of the natural history of abdominal aortic aneurysm and its association with diabetes. In the retrospective analysis the following characteristics were assessed: age, risk factors, type of reconstruction of AAA, and pharmacological treatment of diabetes mellitus.

**Results**

The retrospective analysis comprise the period between 2008-2018. A total of 403 patients are included, of which 8.4% are women and 91.6% - male. The mean age for the women is 69.9 years, and for the men 67.9 years The total number of patients with diabetes mellitus was 53 (13.2%). Of these, 10 patients had ruptured AAA, 10 patients were with symptomatic clinical presentation and 31 patients underwent elective repair. The mean diameter of the AAA in men without or with DM is 65.5mm and 63.1mm, and for women - 65.8mm and 57.6mm respectively.

**Conclusion**

The results revealed that patients with diabetes mellitus are at a lower risk of developing an abdominal aortic aneurysm. Nevertheless, whether the use of pharmacological agents for the treatment of diabetes explain these results should be discussed in further studies. They should focus on development of new therapeutic agents aimed at slowing or even preventing the progression of abdominal aortic aneurysm.

## O83 Comparison between Nd:YAG laser and conventional methods for pulmonary metastasectomy – 7 year trial

### Vasil Yordanov, Teodor Badarov, Deyan Yordanov, Svetlin Nikolov, Alexandra Dimitrova

#### Department of Thoracic Surgery, Military Medical Academy, Sofia, Bulgaria

##### **Correspondence:** Vasil Yordanov

**Purpose**

To assess the role of neodymium:yttrium-aluminum-garnet (Nd:YAG) laser in the resection of pulmonary metastases and compare it with conventional methods using prospective randomized trial conducted in the Department of Thoracic Surgery – Military Medical Academy, Sofia.

**Design**

Randomized trial conducted between 2012 and 2018.

**Site**

Military Medical Academy – Sofia, Bulgaria - Department of Thoracic Surgery

**Materials and methods**

Fifty-eight patients who underwent pulmonary resection due to metastases using two different techniques and distributed randomly: 33 of them treated with Nd:YAG laser (group A) and 26 patients treated with conventional methods (group B). A total of 158 pulmonary lesions are resected, 104 by laser and 54 conventional (electrocautery/stapler). One hundred and thirty eight lesions were diagnoses as active metastases from different primary sites.

**Results**

No deaths occurred during the operations. Nine patients (7 from group B) developed minor complications. In four patients from group B, lesions recurred at the resection site. The usage of Nd:YAG laser is not associated with significantly longer survival (p=0.47). Laser resections allow sparing more functional parenchyma (mean ratio lesion diameter/resected volume, 0.92 vs 1.13, p<0.008). Analyses reveal the significance of the laser for reduction the number of days with postoperative air leakage (3.93 vs 5.22 days), as well as the hospital stay (7.44 vs 9.48 days).

**Conclusions**

Using Nd:YAG laser for resection of pulmonary metastases significantly reduces the loss of parenchyma, postoperative air leakage time and the hospital stay. By means of statistics, the long-term survival was not significantly influenced.

## O84 Effectiveness of hybrid coronary revascularization in patients with intermediate-high SYNTAX Score

### Aliaksandr Charniak, Vladislav Podpalov, Oleg Kozak, Kiryl Rubakhov, Elina Shkrebneva, Alexey Ostrovsky

#### Minsk Scientific and Practical Center of Surgery, Transplantology and Hematology, Minsk, Belarus

##### **Correspondence:** Aliaksandr Charniak

Effectiveness of hybrid coronary revascularization in patients with intermediate-high SYNTAX Score

Aliaksandr Charniak, Vladislav Podpalov, Oleg Kozak, Kiryl Rubakhov, Elina Shkrebneva, Alexey Ostrovsky

Minsk Scientific and Practical Center of Surgery, Transplantology and Hematology, Minsk, Belarus

**Background**

The effectiveness of hybrid coronary revascularization (HCR), combining off-pump coronary artery bypass grafting (OPCAB) and percutaneous coronary intervention (PCI), is a debatable question in modern coronary surgery, especially in patients with intermediate-high SYNTAX Score. The objective of the study: to estimate early outcomes after hybrid coronary revascularization in comparison with conventional OPCAB in patients with multi-vessel coronary artery disease (CAD).

**Materials and methods**

112 consecutive patients with CAD valued with intermediate-high SYNTAX Score were divided into 2 groups: 1st group - 77 patients underwent OPCAB resulting in 2-3 grafts through a full sternotomy; 2nd group - 35 patients with performed HCR. HCR consisted of 2 stages: minimally invasive direct coronary artery bypass grafting and later on the 3d day postoperatively PCI stage was performed.

**Results**

Two groups had no significant differences in main parameters. There were no hospital deaths in both groups. In HCR group there was no need in conversion to cardiac-pulmonary bypass because of hemodynamic instability, early postoperative reoperation due to graft failure, no cerebrovascular accident was seen. The dose of cardiotonic support in intra and early postoperative period was significantly lower in HCR group in comparison with OPCAB group. The level of post-operative high-sensitive troponin I (2,74±5,43 vs 0,082±0,06), amount of intraoperative blood loss (407,5±137,9 vs 193,57±93) were significantly lower in HCR group in comparison with OPCAB group. Treatment in ICU (1,2±0,53 vs 1,06±0,24) after operation was significantly longer in OPCAB group (p<0,05) as well as hospital stay length before discharge (10,9±2,83 vs 7,241,06±2,14) (p<0,05).

**Conclusions**

Hybrid coronary revascularization in group of patients with intermediate-high SYNTAX Score can offer minimization of surgical trauma and better early postoperative results as compared with conventional coronary surgery.

## O85 Carotid stenosis in cardiac surgery patients - a literature review

### Bistra Boneva, Nadelin Nikolov, Margaret Dimova, Detelina Lukanova, Mario Stankev

#### National Heart Hospital, Sofia, Bulgaria

##### **Correspondence:** Bistra Boneva

**Background**

Carotid stenosis is a well-known risk factor for perioperative stroke following cardiac surgery. Simultaneous carotid endarterectomy (CEA) and coronary artery bypass grafting (CABG) has less favorable outcome than the cardiac surgery alone.

**Methods**

A systematic review of randomized trials assessing indications of screening and management in patients, suitable for CABG and coexisting carotid stenosis, was performed.

**Results**

The incidence of stroke after CABG is 1-2%. In patients with unilateral carotid stenosis over 80% undergoing CABG the stroke risk increases to 9%. 86% of the strokes can not be related to carotid involvement. According to available evidence, the relationship between significant asymptomatic unilateral stenosis and stroke after CABG is overestimated.

**Conclusion**

For symptomatic plaques СEA is a gold standard. There is no strong evidence that carotid stenosis is relevant cause of perioperative stroke, beside the bilateral severe bifurcation stenosis. There is no conclusive evidence that prophylactic revascularization for unilateral asymptomatic stenosis in candidates for bypass surgery reduces the risk of perioperative stroke. Thus, the only substantial indication of preoperative revascularization is high grade bilateral carotid stenosis. In patients with severe coronary and carotid artery disease an individualized surgical approach is recommended as the most appropriate management.

## O86 Neocuspidization of the aortic valve with autopericardial tissue: results of 90 consecutive operations

### Igor Mokryk, Vitaly Demyanchuk, Natalia Ponich, Chrystyna Monastyrska, Olga Epanchinceva, Iryna Aksionova, Igor Kuzmich, Borys Todurov

#### Department of Adult Cardiac Surgery, Heart Institute, Kyiv, Ukraine

##### **Correspondence:** Igor Mokryk

**Objective**

Optimal prosthesis for aortic valve replacement (AVR) is still under debate. We evaluated immediate results of Aortic Valve Neocuspidization (AVNeo) carried out at our Institution and performed analysis of most important aspects of perioperative care of these patients.

**Methods**

In the period from December 2016 till November 2018 at 90 patients underwent AVNeo. Surgical technique is based on replacement of every single cusp of aortic valve with neocusp trimmed from patients own pericardium treated with glutaraldehyde.

**Results**

There were 48 males (53.4%). Mean age was 64.2 ± 6.8 years. BMI of more than 25.0 had 73 (81%) patients. 82 (91%) of them were in NYHA Class III and IV. Preoperative EchoCG demonstrated AV average peak pressure gradient of 87.4±22.5 mm Hg; mean aortic annular diameter measured 21.9±1.5 mm. LV EF below 39% had 9 (10%) patients.

AVNeo was successfully performed in every patient. In-hospital mortality occurred in 1 (1.1%) case. There were no conversions to AVR or additional procedures on new valve. Mean bypass and ischemic times were 117.5±16.8 and 85.6±16.4 min respectively. ICU stay was 3.7±2.5 days.

Average peak pressure gradient at discharge from the hospital was 14.5±2,3 mm Hg. Aortic regurgitation was moderate in 4 (4.4%) cases, and trivial or mild in the rest. Half-year follow-up underwent 59 (65.5%) patients. Average peak pressure gradient was 13.6±3,9 mm Hg. Aortic regurgitation was moderate in 3 (3.3%) cases, and trivial or mild in the rest.

**Conclusions**

Ozaki procedure is a new method of biologic AVR. Immediate results of this technique are promising. Method is especially valuable in patients with small aortic root, as it permits to eliminate surgical problem of patient-prosthesis mismatch. Randomized trial is needed to verify advantages and limits of AVNeo in comparison to standard AVR in the mid- and long-term follow-up.

## O87 Posterior leaflet augmentation patch-plasty as a part of complex repair of secondary mitral regurgitation

### Plamen Panayotov, Daniela Panayotova, Vladimir Kornovski, Yavor Peychev

#### Department of Cardiac Surgery, St.Marina University Hospital, Varna, Bulgaria

##### **Correspondence:** Plamen Panayotov

**Background**

There is no agreement in cardiac surgery community regarding surgical treatment of secondary mitral regurgitation (SMR) – many surgeons, disappointed by recurrent mitral regurgitation (rMR), advocate subvalvular apparatus sparing mitral valve replacement with mechanical or biological prosthesis (MVPr), instead of mitral valve repair (MVR). But if repair is successful without rMR, the reverse remodeling of the left heart is significantly better, compared with MVPr.

**Materials and Methods**

Base of theoretical knowledge about mechanisms related to SMR, and publication in medical literature, we consider the augmentation patch-plasty (APP) of posterior mitral valve leaflet (PL) as a useful tool to overcome tethering forces and reduced PL motion – type IIIb mitral regurgitation (MR). We report the echocardiographic criteria and surgical technique (APP + ring annuloplasty ± additional procedure) applied in our first 15 patients with SMR – ischemic and non-ischemic. In all cases preoperative transthoracic (TTE) and intraoperative trans-esophageal (TEE) echocardiography guided our surgical strategy, and gave us the early results. Follow-up criteria are clinical condition, ECG, TTE evaluation for rMR, left heart function and signs of reverse remodeling.

**Results**

Our early and mid-term results are encouraging – all patients with APP left the operation theater and were discharged with no or only trivial MR. There is no early postoperative mortality. Follow-up evaluation is between 3 and 24 months with no signs of significant recurrent MR.

**Conclusions**

SMR is a complex disease and restrictive ring annuloplasty alone can not solve the problem for 30-50% of the patients. More complex approach addressing MV leaflets and subvalvular apparatus is needed to achieve good long-term results. We become our preliminary evaluation with such a complex MVR, but more cases and longer follow-up are necessary to prove the long term success.

## O88 Cryo-ablation for atrial fibrillation as a concomitant procedure for patients with ischemic or valvular heart diseases

### Plamen Panayotov, Ani Raynova, Daniela Panayotova, Yavor Peychev

#### Department of Cardiac Surgery, St.Marina University Hospital, Varna, Bulgaria

##### **Correspondence:** Plamen Panayotov

**Background**

Atrial fibrillation (AF) is registered in 6 – 14% of patients (pts.) for revascularization (CABG), and in about 30% of pts. indicated for mitral valve (MV) surgery. Surgical treatment of AF by cryo-ablation is a highly effective and low risk procedure.

**Materials and methods**

Cryo-ablation as a concomitant to CABG, mitral (MV) or aortic valve (AoV) procedure was introduced in our Department in 2015. Endocardial probe of Nitrous Oxide driven devise was used in 28 patients (18 man), following patients selection criteria – different from permanent AF, preferably lasting less than a year persistent AF, and left atrial (LA) size less than 60 mm. The last two are only relative contraindications in our practice. In valve cases cryo-ablation was performed first, but in CABG patients revascularization is first. Bi-atrial ablation is performed in 16, and only LA ablation in 12 pts. LA appendage was excluded in all. Main indication for surgical treatment was MV disease in 6 pts., AoV – in 5, CABG – in 5, combined CABG + valve procedures – in 12 pts.

Follow-up 3 to 40 months postop was done by telephone interview, clinical evaluation, standard 12 leads ECG, and trans-thoracic echocardiography (TTE).

**Results**

On admition AF was registered in 16 pts. All patients were operated on using cardio-pulmonary bypass (CPB) machine with perfusion 103 to 206 min, aortic cross clamp time 53 to 127 min. All patients, except one, survived the early postoperative period, and 26 are discharged with sinus rhythm (SR). One needed permanent PM implantation because of AV block. Tree months postop 25 pts. are in SR. Two patients died before the end of the first postoperative year.

**Conclusions**

Our experience confirmed the effectiveness of surgical cryo-ablation, even during “learning curve” period. The frequency of recurrent AF is low during the follow-up and cryo-ablation should be a useful tool in daily cardiac surgery practice. Longer follow-up is necessary.

## O89 Secondary mitral regurgitation surgery guided by Echocardiography

### Daniela Panayotova, Plamen Panayotov, Yavor Peychev

#### Cardiac surgery, St. Marina University Hospital, Varna, Bulgaria

##### **Correspondence:** Daniela Panayotova

**Background**

Secondary mitral regurgitation (MR) is a result of left ventricular remodeling and dysfunction in the absence of mitral valve apparatus diseases. It is a more common and with worse prognosis than primary MR. The mechanisms of secondary MR are multifactorial, and very precise imaging assessment is necessary. Transthoracic (TTE), transoesophageal (TEE), and three-dimensional (3D) echocardiography are the main imaging modalities to select the patients with significant secondary MR suitable for surgical intervention.

**Materials and methods**

We present our Heart Team approach, focusing on the possibility for mitral valve repair (MVR) in patients with secondary MR. All patients are examined by TTE preoperatively, intraoperative 2D and 3D TEE, and early postoperative TTE. For all patients with ischemic and non-ischemic significant secondary MR, with a posterior mitral valve leaflet (PL) height less than 15 mm, the surgical correction is a combination of PL augmentation patch-plasty (APP) plus non-restrictive ring annuloplasty, with or without concomitant surgical revascularization (CABG). In all cases echocardiographic prognostic criteria for MV repair failure are taken into account. All the patients underwent PL augmentation patch-plasty combined with non-restrictive ring annuloplasty.

**Results**

In all 8 patients augmentation patch-plasty of PL was successfully applied as a part of MV repair, with intraoperative 2D and 3D TEE control at the end of the procedure, showing no-MR, coaptation length of more than 7 mm, preserved leaflet motion and reduced PL tethering. Early TTE follow-up was before discharge and a month postop. Mid-term results are obtained 5 to 24 months postop.

**Conclusions**

Secondary MR with a severely tethered short PL is a real challenge for the Heart Team in determining optimal treatment strategy. The decision should be guided by an accurate echocardiography assessment – TTE and TEE, giving information about morphology and function of LV and MV.

## O90 Emergency surgical coronary revascularization in patients with acute myocardial infarction

### Alexander Bitsadze, Borys Todurov, Yurii Kharenko

#### SE “Heart Institute of MOH of Ukraine”, Kiev, Ukraine

##### **Correspondence:** Alexander Bitsadze

In the period from January 2015 to January 2018 in the “Institute of the Heart” of ministry of Health of Ukraine were urgently operated 129 patients with acute myocardial infarction (29 (22.5%) - without ST-segment elevation (NSTEMI), 100 (77.5 %) - with ST-segment elevation (STEMI). In all cases the values of troponin I were positive .On all patients were performed emergency coronary artery bypass surgery within 6 hours after coronary angiography .Ratio of men to women was 87 ( 67.4%) and 42 ( 32.6% ), respectively. Mean patient age - 62 ± 12,5 years.In the analyzed group of patients 3- vessel disease of the coronary vessels were detected in 114 (88.3 %) cases , obstruction of the main trunk of the left coronary artery - in 89 ( 69 %) patients , isolated lesion of left main in 5 ( 7 %) , cardiogenic shock was in 13 ( 12.1%) patients , pre-and postoperative intraaortic balloon contrapulsation was used in 35 ( 27.2% ) patients. Average ejection fraction (EF ) of the left ventricle was 35 % or higher in 84 ( 65 %) patients, below 35 % in 36 ( 28 % ) and below 25 % in 9 ( 7%).

In most cases, CABG , we used venous grafts .In 20 patients with stable hemodynamics Left anterior descending artery(LAD) was shunted using the left internal thoracic artery(LITA) .In 31 ( 24.1% ) patients for distal anastomoses were used intracoronary shunts . Complete revascularization ( shunted at least 3 of the arteries) is achieved in 105 ( 81.4 %) patients. In 15 ( 11.7 %) patients , preoperative stents implanted in the infarct - related artery , but because of the inefficiency of this procedure, patients were operated . Total number of grafts on per patient was 2,7 ± 0,4. The average duration of cardiopulmonary bypass was 61 ± 2,6 min. Hospital mortality in patients was 12.4% ( 16 patients died , all with ST-segment elevation ).

## O91 Huge pulmonary arteriovenous malformation diagnosed in 80-year-old patient

### Mertay Boran^1^, Ertay Boran^2^

#### ^1^Department of Thoracic Surgery, Duzce University, Duzce, Turkey; ^2^Department of Anesthesiology and Reanimation, Duzce University, Düzce, Turkey

##### **Correspondence:** Mertay Boran

**Background**

Pulmonary arteriovenous malformations (PAVM) are rare pulmonary vascular anomalies. Although most patients are asymptomatic, PAVMs can cause dyspnoea from right-to-left shunt. Herein we describe a huge PAVM between right lower pulmonary vein and right basiler pulmonary arterial branch diganosed at 80 year old man.

**Case Report**

80 yera old manpresented with dispnea. His Po2, sO2 and Co2 were 61mmHg,% 90, and 31mmHG. Thorax angiographic tomography revealed a 5.5.cm PAVM at right lower superior segment, the right lower pulmonary vein branch and right basilar pulmonary arterial branch performed PAVM(Fig1).Echocardiography revealed severe mitral and tricuspid deficiency. Pulmonary arterial pressure was 45. Right heart failure and pneumonia treatment were applied.Due to elderly age and his comorbidities surgical resection was not performed. Written informed consent was obtained.

**Conclusion**

Pulmonary arteriovenous malformations are rare pulmonary vascular anomalies that may stay undiagnosed for a long years like in our geriatric patient. Treatment options include angiographic embolisation with metal coil or balloon occlusion and surgical excision.Patient’s comorbidities may prevent surgical resection choices.

Written informed consent for publication of the clinical details and/or clinical images was obtained from the patient.

## O93 Intrapulmonary multiple ectopic thyroid- A different diagnosis in a patient with bilateral lung nodules

### Mertay Boran^1^, Ertay Boran^2^, Elif Nisa Ünlü^3^

#### ^1^Department of Anesthesiology and Reanimation, Duzce University, Duzce, Turkey; ^2^Department of Thoracic Surgery, Duzce University, Duzce, Turkey; ^3^Department of Radiology, Duzce University, Duzce, Turkey

##### **Correspondence:** Mertay Boran

**Background**

Ectopic thyroid is a rare developmental abnormality. Ectopic thyroid tissue can be found anywhere along the thyroglossal duct, from the tongue to the mediastinum. Less frequently, thyroid tissue has been reported in the trachea, the heart, the esophagus, the diaphragm, in the duodenum, the biliary tract, the vaginal wall and the sellar region. Bilateral multiple ectopic intrapulmonary thyroid is extremely rare, with only few cases reported in the literature.

**Case report**

A 46-year-old woman was referred to the our thoracic surgery outpatient clinic due to bilateral pulmonary nodules with a one-year history of endometrioma and previous history of partial thyroidectomy performed at the age of 18 years. Her thyroid laboratory tests(TSH 2.091, ST3 3.64, ST4 0.81, chromogranin <2 pg / ml, anti-TPO 0.3 IU / ml, thyroglobulin 7.94 ng / ml) were normal. Thorax CT showed a significant contrast enhancement of 1.4 cm × 1.2 cm nodüle in the right lower lobe posterobasal segment, and a 10x9 mm nodule with contrast enhancement in the left lingula superior. Peripheral subpleural perifissural lymph nodes, not more than 5 mm in the right anterior lobe, lower lobe superior, middle lobe lateral basal segment were observed (Figure1 and 2). PET CT reported SUD maks as 2.1 for two nodular lesions. Right VATS-wedge resection for the largest pulmonary nodule was performed. The pathological evaluation showed that nodule was consisted of follicular thyroid cells. 5 m Technetium 99 pertechnetate Thyroid scintigraphy was within normal limits. Thyroid Ultrasonography was perfomed and the thyroid gland parenchyma revealed a heterogeneous isoechoic nodule of 12x17 mm in the right lobe and several cystic necrotic contents of 13x18 mm in the left thyroid lobe. The pathology of thyroit glands nodules biyopsis revealed regressed colloidal nodules. Postoperative 1 year follow-up thorax CT showed no progression of the nodule in left lung lingula. Written informed consent was obtained.

**Conclusion**

Multiple ectopic intrapulmonary thyroid is extremely rare and pulmonary metastases were initially considered as the most likely cause of the multiple pulmonary nodules. Ectopic intrapulmonary thyroid tissue should be carefully monitored due to the risk of malignancy.

**Referances**

1. Noussios G, Anagnostis P, Goulis DG et al Ectopic thyroid tissue: anatomical, clinical, and surgical implications of a rare entity. European Journal of Endocrinology 2011;165:375–382

2. Ryu HS, Chung YJ, Chong S, Lee JI. Ectopic intrapulmonary thyroid tissue mimicking

Written informed consent for publication of clinical details and/or clinical images was obtained from the patient.

## O95 Embologenic aorta with multiple peripheral embolism

### Ognian Matkov^1^, Angel Marinov^2^, Rosen Razboynikov^1^, Ivaylo Nenov^2^, Desislava Bojadjieva^3^, Boris Tsankov^1^, Georgi Voynov^1^

#### ^1^Clinic of vascular surgery UH “Heart and Brain”, Medical University Pleven, Bulgaria; ^2^Clinic of cardiac surgery UH “Heart and Brain”, Medical University Pleven, Bulgaria; ^3^Clinic of cardiology UH “Heart and Brain”, Medical University Pleven, Bulgaria

##### **Correspondence:** Ognian Matkov

**Background**

Acute arterial embolism of major limb vessels is severe problem worldwide, demanding adequate diagnosis and surgical treatment. Major causes of embolism are valvular lesions, endocarditis, acute aortic syndrome, aortic atherosclerosis, cardiac tumors, etc. These conditions are frequently accompanied with detachment of thrombi, atherosclerotic plaques or tumor particles.

**Case report**

We present the diagnostic modalities and surgical treatment of 47 y.o. patient with rare embologenic aorta and multiple peripheral embolism. Coronarography due to suspicion for myocardial ischemia showed no evidence of coronary pathology one year ago. Upon admission, the patient had signs of acute arterial insufficiency of left lower limb and after CT-angiography, a surgical embolectomy was performed. Within the next 7 days the patient was re-operated twice on left side and once on right due to new episodes of acute ischemia of the lower limbs. Transthoracic echocardiography didn’t detect thrombotic masses in cardiac cavities. Transesophageal echocardiography showed tumor formation above the right coronary ostium. Aorto-coronary bypass and tumor extirpation was done. Histological studies showed mixed thrombus.

**Conclusion**

Transesophageal echocardiography should always be perform alongside with transthoracic echocardiography and CT-angiography when searching the source of multiple peripheral embolism. Embologenic aorta should always be considered when usual causes such as thrombi in the left cardiac cavities, infectious endocarditis and cardiac tumors are rejected as a source of peripheral embolization.

The patient provided written consent to the publication of clinical or personal details.

## O96 Substitution of defects and thromboses of popliteal artery with autovenous conduits from small saphenous vein

### Ognian Matkov^1^, Yovcho Topalov^1^, Georgi Marinov^2^

#### ^1^Vascular and endovascular clinic of University Hospital “Heart and brain”, Medical University Pleven, Bulgaria; ^2^Medical University “Prof. Dr. Paraskev Stoyanov” Varna, Bulgaria

##### **Correspondence:** Ognian Matkov

**Background**

Acute ischemia below the knee involving the popliteal artery requires prompt operative revascularization to avoid necrosis of the limb.

**Materials and methods**

The authors look through the possibilities for surgical treatment of defects and thromboses of the popliteal artery with autologous angioplasty “in situ” using the small saphenous vein. They discuss the opportunities for restoring the damaged popliteal artery using two surgical techniques: Angioplasty after thrombendarterectomy using a flap from the small saphenous vein, sparing the blood supply of the conduit; Bypass procedure, using the small saphenous vein, while preserving the blood supply of the transplant.

**Conclusions**

Preserving the viability of the vascular endothelium of the autovenous transplant has a prophylactic effect against its thrombosis.

## O97 Hybrid approach in acute aortic dissection DeBakey type IIIb coexisting with dissecting abdominal aneurysm. Clinical case

### Valentin Govedarski^1^, Elitsa Dimitrova^1^, Emil Hadzhiev^1^, Peyo Simeonov^2^, Todor Zahariev^1^, Gencho Nachev^3^

#### ^1^Department of Vascular Surgery, University hospital “Saint Ekaterina”, Sofia, Bulgaria; ^2^Department of Cardiology, University hospital “Saint Ekaterina”, Sofia, Bulgaria; ^3^Department of Cardiothoracic Surgery, University hospital “Saint Ekaterina”, Sofia, Bulgaria

##### **Correspondence:** Valentin Govedarski

**Background**

Acute aortic dissection is associated with high mortality and morbidity determined by location of the affected aortic segment. The original description of a distal (Type III) aortic dissection according to DeBakey originated at level of the left subclavian artery.1 Type IIIa aortic dissections are confined to the descending thoracic aorta, while Type IIIb aortic dissections extend into the abdominal aorta.

**Case report**

We present a case of 57- year old patient with gluteal claudication 3 days. The pain initiated from the chest irradiating to the lower limbs. Neurologist consultation was conducted and abdominal aortic aneurysm with diameter 64mm was accidentally discovered. Contrasted computed tomography was made- aortic dissection DeBakey Type IIIb beginning from, the left subclavian artery to the bifurcation of the abdominal aorta. No hypo-perfusion of the visceral vessels detected. Abdominal aortic dissecting aneurysm (AAA) with maximal diameter 704/720mm and intimal flap followed to the left common iliac artery. Hybrid approach was performed with thoracic endovascular aortic repair (TEVAR) and surgical AAA replacement. Guidewire from the right common femoral artery was placed to the ascending aorta in the true lumen followed by replacement of the AAA with Dacron prosthesis. Endoproshesis was delivered subsequently.

**Conclusion**

Hybrid surgical and endovascular techniques are increasingly used and developed, improving the outcomes for complex patients with acute aortic dissection. With the above mentioned procedure we have the opportunity to successfully deliver both the abdominal prosthetic graft and the endoprsthesis to the true lumen. The combination of surgical abdominal aortic replacement and TEVAR is a reliable option with limited complications and mortality.

**References**

1. Surgical management of dissecting aneurysms of the aorta. DeBakey ME, Henly WS, Cooley Da, Morris Cc Jr, Crawford Es, Beall Ac Jr, Thorac Cardiovasc Surg. 1965 Jan; 49():130-49.

2. Management of acute aortic dissections. Daily PO, Trueblood HW, Stinson EB, Wuerflein RD, Shumway NE Ann Thorac Surg. 1970 Sep; 10(3):237-47.

## O98 Nutcracker syndrome- surgical treatment. Clinical cases

### Valentin Govedarski^1^, Milena Angelova^1^, Dimitar Chernev^1^, Ilyan Petrov^1^, Borislav Denchev^3^, Todor Zahariev^1^, Gencho Nachev^2^

#### ^1^Department of Vascular Surgery and endovascular treatment, University hospital “Saint Ekaterina”, Sofia, Bulgaria; ^2^Department of Cardiothoracic Surgery, University hospital “Saint Ekaterina”, Sofia, Bulgaria; ^3^Department of Vascular Surgery, Universiry hospital “Deva Maria” Hospital, Burgas, Bulgaria

##### **Correspondence:** Valentin Govedarski

**Background**

The Nutcracker phenomenon (NCP) is the compression of the left renal vein (LRV) leading to an increase in the pressure gradient between the LRV and the inferior vena cava and development of left-renal venous hypertension. The Nutcracker syndrome (NCS) is the clinical equivalent to the NCP characterized with complex of symptoms with significant variations.Because of the variety of clinical symptoms and lack of consensus on the diagnostic criteria, the exact incidence of occurrence is unknown.

**Case report**

We present three clinical cases in patients with Nutcracker syndrome and the applied surgical methods.

First clinical case- 25- year old man with anterior variant of NCS, treated conservatively and followed up in one-year period. Due to worsening clinical symptoms, left renal vein reimplantation was performed.

Second clinical case- 52- year old man with posterior variant of NCS (as an etiological factor- retroaortic left renal vein compressed by abdominal aortic aneurysm (AAA) without rupture). Resection of AAA and reimplantation with Dacron prosthesis was performed followed by debridement of left renal vein, left testicular vein and the retroperitoneal cavity.

Third clinical case- 36- year old women with anterior NCS coexisting with Willkie syndrome. Debridement of the fibro-lymphoid tissue surrounding the left renal vein and the para-aortic cavity with decompression of the LRV and division of the ligament of Treitz were performed.

**Conclusion**

The correlation between LRV compression and clinical symptoms remains challenging and surgical interventions are indicated in severe clinical symptoms and lack of conservative treatment options. The NCS consists of LRV compression with various etiologies and may coexist with other organ-confining diseases.

Written informed consent for publication of their clinical details and/or clinical images was obtained from the patients.

## O99 Surgical treatment of symptomatic carotid tortuosity – 13 years of clinical experience

### Valentin Govedarski, Svetoslav Dimitrov, Sofia Antonova, Тodor Zahariev, Gencho Nachev

#### Department of Vascular and Endovascular Surgery, University Hospital “St. Ekaterina”, Sofia, Bulgaria

##### **Correspondence:** Valentin Govedarski

**Introduction**

Extracranial carotid artery elongation presenting as tortuosity, kinking or coiling can be discussed as a reason for hemispheric and nonhemispheric symptoms. Surgical treatment of symptomatic internal carotid artery (ICA) elongation provides prevention of ischaemic stroke and progression of cerebrovascular insufficiency by improving the brain. The aim of our study was to evaluate, the incidence and the early and late postoperative results in patients with symptomatic isolated ICA elongations.

**Material and methods**

For a period of 13 years (2006-2018) 814 carotid reconstructions were performed in our unit. 213 (26.28%) of them were in 170 patients with isolated ICA symptomatic elongations. The routine technique consisted of partial resection of the elongated ICA followed by its reimplantation to the common carotid artery. Transcranial Doppler (TCD) monitoring was performed to assess the blood flow changes in the middle cerebral artery during all procedures. The operated carotid artery blood flow was evaluated simultaneously by flowmetry in 88 cases. Patients were followed up at the 1st and 3rd month postoperatively.

**Results**

In the early postoperative period in 1(0.005%) patient an ipsilateral acute thrombosis occurred, causing homolateral stroke and death. Transitory ischemic attacks (TIA), reperfusion syndrome and myocardial infarction were registered in 7 (0.03%) other cases. A few patients with hypertension and traumatic lesions of n. hypoglossus, r. reccurens nervi vagi, n. facialis and n. vagus were diagnosed. Most of the patients with hemispheric symptoms had complete remission. During the follow-up in 158 cases a reduction of the neurological symptoms was observed and they were completely resolved by the third postoperative month.

**Conclusion**

The incidence of clinical manifestation of symptomatic isolated ICA elongations proved not to be so rare – 26%. Endovascular treatment in these cases is not an option for treatment. Surgery of symptomatic ICA elongations is safe, effective and reliable method for stroke prevention and reduces the symptoms of cerebrovascular insufficiency.

**Key words**

carotid elongation, surgical treatment, stroke prevention

## O100 Arsenic in myocardium: analysis of tissue samples from living human hearts

### Nazmul Hosain^1^, Fahmida Wali^2^, Jibran Alam^2^, Rubhana Rakib^3^, Farzana Amin^4^, Abdul Chowdhury^1^, Fazle Maruf^1^, Mamunur Rahman^5^, Zillur Rahman^6^, Mohammad Al-Forkan^2^

#### ^1^Department of Cardiac Surgery, Chittagong Medical College & Hospital, Bangladesh; ^2^Department of Genetic Engineering & Biotechnology, University of Chittagong, Bangladesh; ^3^International Center for Diarrheal Diseases and Research Bangladesh, Dhaka, Bangladesh; ^4^Northern Health, British Columbia, Canada; ^5^Department of Cardiac Anesthesia, Chittagong Medical College & Hospital, Bangladesh; ^6^Department of Pathology, Chittagong Medical College & Hospital, Bangladesh

##### **Correspondence:** Nazmul Hosain

**Objective**

Groundwater contamination of Arsenic, a toxic element present in the environment, is an alarming concern. It may accumulate in drinking water, plants, crops, vegetables and finally to the human consumers through consumption of these. Arsenic deposition in various tissues may cause serious human health hazards. The common sites of Arsenic deposition include hair, nail and skin. Deposition of high level of Arsenic in myocardium may be related to cardiac ailments. Little is known about myocardial Arsenicois. Objective of this study is to determine the Arsenic level in myocardial samples collected from living human hearts during open heart surgical procedures.

**Methods**

Atrial myocardium samples were collected from 50 adult patients undergoing open heart surgical procedures at the Department of Cardiac Surgery Chittagong Medical College Hospital and National Heart Foundation Hospital Dhaka , Bangladesh between July 2017 and June 2018 in collaboration with the Department of Genetic Engineering and Biotechnology, University of Chittagong. These patients included Correction of congenital defects, valvular replacements and CABGs. A small piece of myocardium was collected from the right atrial appendage within the venous cannulation purse-string site. Arsenic measurement was performed by Hydride Generation Atomic Absorption Spectrometry (HG-AAS) in ICDDRB, an international health research organization in Dhaka.

**Results**

In the atriIal myocardium Arsenic concentration ranged from 1 to 11.75 ppb with a Mean±SE value of 4.07±0.40 ppb. 40 samples had less than 5 ppb of As; 8 samples had between 5 and 10 ppb, where as 2 samples had more than 10 ppb. Further results from this ongoing study would provide better understanding of myocardial Arsenicosis in the future.

**Conclusions**

Though very little in amount, the atrial myocardium contains various levels of Arsenic. Further studies and analyses are going on to figure out any relationship between the level of Arsenic and cardiac diseases.

## O101 Concept of surgical cardiology featuring frustrations, foresight and future of the cardiac surgeons

### Nazmul Hosain^1^, Abdul Chowdhury^1^, Minhazur Chowdhury^2^, Farzana Amn^3^, Anisuzzaman^1^, Fazle Maruf^1^, Mamunur Rahman^2^

#### ^1^Department of Cardiac Surgery, Chittagong Medical College & Hospital, Bangladesh; ^2^Department of Cardiac Anesthesia, Chittagong Medical College & Hospital, Bangladesh; ^3^Northern Health, British Columbia, Canada

##### **Correspondence:** Nazmul Hosain

Cardiac surgery owns a history of merely a few decades. Once thought absolutely impossible operations of the heart became a reality thanks to a series of inventions and innovations during the 1950s. Despite technological advances Cardiac Surgery seems to have reached its peak and now it’s likely to take a downward numerical trend due to continued rapid expansion of medical and intervention cardiology. This might change the spectrum of cardiac surgery as a subject in future. The Cardiac Surgeons might have to mould themselves to a different role.

**Methods**

The various historic events, related of the development of Cardiac Surgery and Invasive Cardiology were explored. The trending pattern in the number of cardiac surgery and Cardiac intervention performed during chronologically successive years was analyzed. Related printed and electronic materials have been made thoroughly searched.

**Results**

The arena of Invasive Cardiology is rapidly expanding through availability of newer technology and innovations. Difficult newer coronary, valvular and congenital interventions are being practiced by the cardiologists outpacing and invading the cardiac Surgery territory. Newer pharmacological, genetic and molecular tools are arriving to improve the scope of treatment. All these are shrinking the area of Cardiac Surgery. In future there would be requirement of a very few in number but highly skilled Cardiac Surgeons.

**Conclusion**

The newer technological developments are gradually shrinking the arena of Cardiac Surgery. The number of cardiac operations required is likely to be grossly reduced in next two decades as many procedures won’t be required anymore. The cardiac surgical procedures would be much fewer in number but much difficult to perform. There would be requirement of a few surgeons, fewer surgical procedures with requirement of high skills. Capacity building would becpme very difficult. Hence the new generation of surgeons should acquire diagnostic, echocardiograpic and cath lab skill as well as efficiency in minimal invasive surgery. They should consider preparing themselves not only as Cardiac Surgeons but rather as complete Surgical Cardiologists.

## O102 Sub-lobar resection versus radio-frequency ablation in the treatment of stage I NSCLC in high risk patients

### Nizar Asadi^1^, Vladimir Anikin^1^, Emma Beddow^1^, Paras Dalal^2^

#### ^1^Thoracic Surgery Department, Harefield Hospital, Royal Brompton and Harefield Foundation Trsut, London, UK; ^2^Radiology Department, Harefield Hospital, Royal Brompton and Harefield Foundation Trust

##### **Correspondence:** Nizar Asadi

**Introduction**

Lobectomy and systemic lymph nodes evaluation is currently the gold standard treatment of patients with stage I NSCLC. In the elderly population, however, with poor lung function and multiple comorbidities, lobectomy becomes more challenging. It may significantly increase mortality and morbidity, resulting in compromised quality of life in this patient group. We evaluated the efficacy of alternative treatments for patients with stage I NSCLC not fit for lobectomy.

**Methods**

Prospective data collection of all patients with Stage I NSCLC admitted to our department for treatment. All patients has been previously discussed in the lung MDT and identified to be not fit for lobectomy. Patients were considered for sub-lobar resection as first choice and for RFA if surgical option was excluded.

**Results**

From January 2011 to June 2017 171 patients with stage I NSCLC were identified not fit for lobectomy in our department. 83 (48%) were female and the median age of the cohort was 73 years. 108 (63%) patients underwent sub-lobar resection and 63 (37%) underwent radiofrequency ablation. The mean age for surgical patients and RFA patients was respectively 71 vs 74 year old (P=0.11). 72 (67%) of patients underwent sub-lobar resection were not fit for lobectomy due to poor respiratory function test and 36 (33%) had severe comorbidities. Patients underwent RFA had significantly worse respiratory lung function with mean of FEV1 respectively 60% vs 74% (P=0.001). The overall 5 years survival was 68% with median of 73 months. Comparing the two groups, patients underwent sub-lobar resection had significant better survival than those underwent RFA with respectively 5 years survival of 89% vs 49% (P<0.001) (Graph 1).

**Conclusion**

Lung resection with lymphadenectomy remains treatment of choice for patients with stage I NSCLC; however sub-lobar resection is a valid option for patients with high risks for lobectomy. RFA showed excellent results in terms of disease control and survival; patients not fit for surgery should be strongly recommended for RFA treatment if other options are not available.

## O103 Successful multistage hybrid treatement of aaa and intraluminal thrombus in thoracoabdominal aorta- case report

### Stefan Stefanov^1^, Ivo Petrov^1^, Vlado Galacev^1^, Petar Valcanov^2^

#### ^1^Acibadem City Cliniic, Sofia, Bulgaria; ^2^Medical University, Varna, Bulgaria

##### **Correspondence:** Stefan Stefanov

The combined pathology of the thoracic and abdominal aorta is always a challenge in the modern vascular surgery. In contrast to aortic aneurysms and aortic dissections in the thoracoadominal segment, other pathological conditions are encountered, though rarely, in which the endovascular or surgical approach are inappropriate. In these cases, the hybrid treatment method is the only option.

The case is about a patient with proven juxtarenal abdominal aortic aneurysm and intraluminal thrombus in the descending and abdominal aorta. The reduction of the lumen reaches 80% in the visceral aortic segment. High-grade stenosis of the left renal artery and left sub-articular artery were diagnosed.

A stepwise hybrid treatment was performed. As a first step, a left subclavian artery was debranched by a left carotid artery reposition to provide a proximal landing zone. In the second stage, resection of the abdominal aneurysm above the level of the left renal artery, which is reanastomosed by a lateral graft to the aortic prosthesis.

As a final - implantation of endovascular stent graft from the level of the left carotid artery to the middle of the descending aorta. In a distal direction, two open aortic stents were implanted to a truncus coeliacus level to isolate the intraluminal thrombus and to normalize the aortic lumen. After a smooth postoperative period, the patient left the hospital.

The present case is a rare combination of dilated and thrombotic pathology of the thoraco-abdominal aortic segment without the option of a single endovascular or surgical solution. The applied stepwise hybrid method successfully solves the severe combined vascular pathology in the thoracic and abdominal aortic segment and minimizes the risk of complications occurring in a classical open surgery.

## O104 Surgical treatment of ruptured thoracoabdominal aortic aneurysm type V – case report

### Stefan Stefanov^1^, Vlado Galacev^1^, Asen Kelcev^1^, Ivet Tasheva^1^, Petar Valcanov^2^

#### ^1^Acibadem City Cliniic Sofia, Bulgaria; ^2^Medical University, Varna , Bulgaria

Even in the endovascular era, the treatment of thoracoabdominal aneurysms remains one of the greatest challenges in modern cardiovascular surgery. The rupture of these aneurysms significantly complicates their treatment due to the urgency of the condition and the dramatic deterioration in a short term of the functions of all the organs and systems of the patient. The treatment team faces a tremendous responsibility within a limited time to stabilize the patient in shock, to accurately diagnose, select and perform an error-free, extremely difficult, huge volume and lengthy surgery, which corresponds to the specific anatomy and concomitant illnesses of the patient .

The case is about a male patient aged 58 years who has been admitted to the Clinic due to an emergency in deteriorated condition and chest and abdominal pain lasting for several hours.

From CT Angiography - data on a thoracoabdominal aneurysm Crawford Type V with a rupture of the thoracic part to the mediastinum. Coeliac trunc and upper mesenteric artery originated from the lower pole of the aneurysmal sac. Double left renal artery. Existence of tortuosity of the common iliac arteries. The case was discussed by a team of vascular specialists, invasive cardiologists and cardiac surgeons. The possibility of endovascular treatment was discarded due to the anatomical features of the iliac vessels and a decision was made for open surgical intervention.

A resection of the thoracoabdominal aneurysm was performed by replacing of tube graft and replantation of celiac trunk and upper mesenteric artery such as Carrel patch. Lower limb perfusion and visceral vessels was made with an external pump from an extracorporeal perfusion system.

The patient left the hospital on the 10th postoperative day with stabilized vital functions. On a check-up after 4 months, the patient is in good general condition and functioning reconstructions. The surgical behavior in emergency patients with TAAA rupture and anatomical peculiarities without the possibility of endovascular treatment is discussed.

## P1 Improving the results of the surgical treatment of patients with acute arterial occlusion of leg

### Yuri Kazakov, Andrei Kazakov, Andrei Gerasin, German Dokshokov, Maxim Strakhov

#### Department of vascular surgery, Tver regional hospital, Tver, Russia

##### **Correspondence:** Andrei Kazakov

**Purpose**

To improvise the results of the surgical treatment of patients with acute arterial occlusion of the lower extremities.

**Methods**

Results of surgical treatment of 95 patients (mean age 67.4 ± 3.7 years) with acute arterial occlusion of the lower limbs were studied. Embolism occurred in 41 (43.2%) patients, thrombosis without the identified causes - 4 (4.2%), thrombosis in the background of atherosclerosis - in 50 (52.6%). 12.6% of patients were hospitalized with ischemic period of more than 24 hours, and the vast majority of 44.2% of people - more than 48 hours. Acute ischemia according to the VS classification. Savel'eva I B stage was recorded in 7 patients, II A-in 28, II B-in 24, III A-in 18, III B-in 18. For the study of the main arteries of the lower extremities, angiography and duplex ultrasonography were used.

All patients underwent an emergency surgical removal of the emboli to restore the main blood flow to the limb. In 14 (14.7%) patients, it was not possible to perform thrombectomy due to the presence of widespread atherosclerotic lesions of arteries. In 5 patients, shunting operations were performed, 6 patients required limb amputation. 81 (85.2%) patients underwent thrombectomy from the arteries of the lower extremities. 16 (16.8%) patients with severe limb ischemia and severe skeletal muscle damage, a fasciectomy was performed on the shin.

**Results**

In 10 (12.4%) patients in the early post-operative period there was a re-thrombosis of the reconstruction zone, which required repeated thrombectomy. Limb amputation was performed in 19 (23.4%) patients after thrombectomy, and in 16 patients a multilevel atherosclerotic lesion of arteries was revealed. 9 (9.4%) patients died, including 5 with atherosclerotic lesions of the arteries of the extremities due to the development of acute myocardial infarction, stroke, and multi-organ failure. In 52 patients, 2 - 5 days after thrombectomy, we underwent with the angiography of the arteries of the lower extremities. In 38 (73%) people, we detected a significant atherosclerotic lesion of the arteries of the arterial bed. The patients, thereafter, underwent various reconstructive surgeries-endovascular and shunt interventions.

**Conclusion**

In 53% of patients with acute arterial occlusion there was a pronounced atherosclerotic lesion in the arteries of the lower limbs. After the standard thrombectomy, all these patients were advised to perform angiographic examination of arteries of the limb with subsequent reconstructive operations.

## P2 Endoscopic stapler selection on bleeding at the vascular stump in thoracic arterial transection for lung cancer lobectomy

### Yoshio Tsunezuka

#### Department of General Thoracic Surgery, Ishikawa Prefectural Central Hospital, Kanazawa, Ishikawa Prefecture, Japan

**Background**

Thoracoscopic endoscopic staplers are essential tools for surgical procedures performed to treat pulmonary diseases including lung cancer, but sometimes inraoperative bleeding or oozing can occur from transected tissue, can obscure the surgical area and is a source of stress for surgeons. Recently, powered staplers are used to cut pulmonary vessels and lung tissue because those staplers are associated with a reduced risk of bleeding compared to manual staplers because they minimise the impact of surgeons’ hand tremors when firing. We retrospectively studied and analized the relationships between various types of staplers and bleeding following stapling of pulmonary arteries.

**Methods**

Patients who underwent VATS and open thoracic surgery for non-small cell lung cancer at Ishikawa Prefectural Central Hospital between 2012 and 2018 were studied. Incidences of bleeding during stapling of the right upper branch of the pulmonary artery (Truncus superior) with three groups of endoscopic staplers were assessed: Group 1: ECHELON powered vascular stapler [PVS], Group 2: Medtronic Endo-GIATM powered stapler, Group 3: ECHELON and Medtronic manual staplers. Stapler characteristics were investigated to determine associations with bleeding. Patients with diabatic, empyema/pyothorax, and/or steroid intake, due to the potential vulnerability of the pulmonary artery, and patients on anti-coagulation medicines, due to increased hemorrhagic tendency. Also, cases with recurrent cancer, neoadjuvant chemotherapy, chemoradiotherapy, and robot assisted cases were excluded in this study.

**Results**

Of 239 lung cancer patients, 82 cases (34.3%) were Group 1, 94 cases (39.3%) were Group 2 and 63 cases (26.3%) were Group 3. In each group, the majority of patients were male (53.2–91.5%), mean age was 36–88 years, and most patients received a VATS right, upper lobectomy resection (80.5–88.9%). No bleeding cases required suturing in all cases.Twelve cases used soft coagulant device and achieved hemostasis. Bleeding following stapling occurred in 17/94 (18.1%) cases in Group 2 and in 4/63 (6.3%) cases in Group 3. No bleeding occurred in Group 1. The loaded ECHELON PVS and Endo-GIA™ iDrive™ gray cartridge combination had the greatest and smallest closed anvil jaw gaps (>0.63 μm and <0.15 μm, respectively). Endo-GIA™ i-Drive white and gray reload combinations resulted in ruptures in the inner and middle membrane of the pulmonary artery. No ruptures were observed using the ECHELON PVS. Histological sectioning revealed that stapling of the pulmonary artery with Endo-GIA™ i-Drive loaded with white and gray cartridges resulted in ruptures in the inner and middle membrane of the pulmonary artery. No ruptures were observed when using the ECHELON PVS.

**Conclusion**

An excessively narrow gap between the cartridge and anvil may disturb the blood vessel wall and result in bleeding . Appropriate endoscopic stapler choice is therefore important to reduce bleeding incidences during pulmonary artery transection.

## P4 Successful conservative treatment of Boerhaave syndrome related to gastric ulcer with severe pyloric stenosis

### Fatmir Caushi, Engjell Bejtaj, Alban Hatibi, Ilir Skenduli, Ornela Nuredini, Fahri Kokiqi, Alma Cani

#### Department of Thoracic Surgery at University Hospital ‘Shefqet Ndroqi” of Tirana, Albania

##### **Correspondence:** Fatmir Caushi

**Background**

Boerhaave syndrome is a rare pathology consisting of spontaneous rupture of the esophagus, generally induced by persistent vomiting due to a sudden increase in its intraluminal pressure. In 50% of cases, it manifests by Mackler's triad: vomiting, lower thoracic pain and subcutaneous emphysema. Delayed diagnosis may result in serious complications and high mortality. This case highlights that conservative management may be a viable option in certain situations.

**Case report**

We report a case of Boerhaave syndrome related to gastric ulcer with pyloric stenosis. A 52-year-old male presented in emergency complaining of severe chest pain, progressive dyspnea, and general weakness. He had suffered episodes of forceful foul-smelling vomiting and epigastric pain 36 hours before presenting. On thoracoabdominal CT scan performed in a regional hospital, it was noticed bilateral pleural effusion, pneumomediastium and gastrectasia. Past medical history revealed gastric ulcer for 25 years. On physical examination, the patient appeared pale, dehydrated and malnourished. The abdomen was tense with tenderness in the epigastric area and clapotage. Thoracostomy tubes were placed in both sides, draining 1.5L of brownish fluid and air in the right hemithorax, and 1L of similar fluid in the left side. Nasogastric suction was performed. Oral enhanced chest CT scan revealed rupture of thoracic esophagus and obstruction of contrast passage to duodenum. After 24 hours of reanimation, the patient underwent surgery to bypass stenotic passage by performing a posterior retrocolic gastroenterostomy and to provide nourishment through Kader gastrostomy. He was dismissed from the hospital after 15 days in good conditions.

**Concluions**

We present a case of Boerhaave syndrome where the patient was managed by reanimation and gastrostomy as a feeding route for the patient. This conservative treatment proved to be successful, accompained by gastroenteric anastomosis intervention for pyloric stenosis.

The patient provided written consent to the publication of clinical or personal details.

## P6 Four new clinical symptoms in thrombophlebitis of the deep veins

### Yovtcho Topalov, M. Topalova

#### MHAT Heart and Brain, PLEVEN MHAT “Serdica”, Sofia, BULGARIA

##### **Correspondence:** Yovtcho Topalov

Clinical symptoms play a basic role in diagnosis of the diseases. The authors observe 5500 patients with thrombophlebitis of the major veins, describe four new and less familiar symptoms for clinical diagnosis of the diseases.

The first symptom is called “The Symptom of the brachio-jugular reflux”.

The second is “The Symptom of the straightened gluteal fold”.

The third is “The symptom of the oedemic-hypotrophic extremity”.

The fourth is “The Modified Hommans Symptom”.

There is no doubt that these are new clinical described symptoms that play a significant role in prompt the clinical diagnosis in the veins thrombophlebitis.

## P7 Treatment of operative “closed cavities” and traumatic decollement of the skin with the help of conciliation displaced of fixed points “antiseptic dra

### Iovcho Topalov^1^, Kalina Casadiego^2^

#### ^1^Mental Heart and Brain Hospital, Vascular and Endovascular Clinic Surgery, Pleven, Bulgaria; ^2^Ullern High School, Oslo, Norway

##### **Correspondence:** Iovcho Topalov

Conciliation displaced of fixed points were introduced by I. Topalov in patients with post-phlebitis syndrome in1967. Their application has also been permitted in patients with amputation of the mammary gland (1968), retroperitoneal hematomas (1974), extensive traumatic decollement of the skin (2016), and others.

To improve the drainage functions of the sutures, in 2018 the authors created “drainage sutures”. Moving through several stages and optimizing the drainage technology, the authors developed their technique creating “antiseptic drainage sutures” with an optimal number of drainage channels, occupying 50% of 35% of the thread volume.

The authors created with their own original preparation the antiseptic effect of the threads.

As a counter of cutting the skin from the fixing sutures, the authors also developed “antiseptic compression bands” that facilitates the prophylaxis of post-operative wounds such as seromas and hematomas and helps the healing process.

## P10 Pulmonary hydatid disease: experience of tertiary hospital in Palestine

### Moath Nairat, Ahmad K Darwazah, Hamad Madi, Mohammad Lutfi, Mohammad Eida, Adnan Abdelhadi, Baraa Ibrahim, Saeed Mostafa

#### Department of Cardiothoracic and General Surgery, Palestine Medical Complex – Ramallah

##### **Correspondence:** Moath Nairat

**Background**

Hydatid disease or hydatidosis is a parasitic infection caused by a tapeworm of genus Echinococcus. Lung is the second most common site affected after liver. It has a variable clinical course, ranging from being asymptomatic to a massive hemoptysis. Surgery is the main therapeutic approach to manage pulmonary hydatid disease (PHD). It is considered a common surgical problem in Palestine as well as in all Mediterranean countries. So, in this study we aim to report the frequency and outcome of PHD along with our experience in managing this surgical condition in Palestine.

**Methods**

We conducted a retrospective study in all patients who had been admitted to the Palestinian Medical Complex, in which it is a tertiary hospital, from 2012 to 2016 and underwent a surgical management for pulmonary hydatid cyst.

**Results**

Among 114 patients who had pulmonary hydatosis, 60 (53%) patients were male and 54 (47%) were female. The disease were more common in age group of older than 20 years with 65 (57%) patients (P value > 0.05). 73 (64%) of them were diagnosed to have isolated lung hydatid cyst while 34 (30%) had multiple hydatid cysts affected both lung and liver. The most common affected lung's lobe was right lower lobe (59%). Regarding surgical approaches, enucleation was done in 69 (60%) patients, cystectomy in 29 (25%), segmentectomy or lobectomy in 6 (5%) and thoracotomy with phrentomy at one stage was done in 18 (15%) patients who had combined cyst in right lung and liver. Post-opetrative complications developed in 25 (21%) patients with recurrence was recorded in 12 (10%) patients and mortality rate occurred only in one (0.8%) patient.

**Conclusion**

Pulmonary hydatid disease can be managed surgically with many different approaches depending on the anatomical location, status of the cyst and the general condition of the patient. Also, concomitant cysts in the right lung and subphrenic liver can be managed safely in only one stage operation to avoid another operation.

## P12 Intrathoracic thyroid carcinomas: report of three cases

### Alkin Yazicioglu^1^, Furkan Sahin^1^, Mahmut Subasi^1^, Nesrin Turhan^2^, Neslihan Zengin^2^, Erdal Yekeler^1^

#### ^1^Department of Thoracic Surgery, Ankara City Hospital, Ankara, Turkey; ^2^Department of Pathology, Ankara City Hospital, Ankara, Turkey

##### **Correspondence:** Alkin Yazicioglu

**Background**

A retrosternal goiter is defined as a goiter with ≥50% of its mass located in the mediastinum. The incidence accounts for 1-20% of all thyroidectomy cases. The thoracic and mediastinal localization of thyroid carcinomas is uncommon, and the mass may grow larger in the event of delayed diagnosis.

**Case report**

A 52-year-old female patient who presented with chest pain was diagnosed with a mediastinal mass. A mass measuring 11x10x8 cm was detected in the anterior mediastinum, extending towards the hilus and surrounding the right subclavian-artey, with a SUVmax uptake of 5.26 (Figure-1). A sample obtained by fine needle biopsy led to diagnosis of a papillary thyroid carcinoma. The mass was excised en-block via right thoracotomy + partial sternotomy + collar incision.

A 63-year-old male patient had previously underwent three goiter operations (1977,1993,2005). During follow-up, anterior mediastinal mass with lobulated edges measuring 6.3cm at its longest diameter was detected. Additionally, a further mass was detected on the manubrium sterni, caused the destruction of bone cortex (Figure-2). The mass, with SUVmax value of 3.50, was biopsied and diagnosed as well-differentiated thyroid carcinoma. The mass and manubrium sterni was excised en-block with collar incision. The pathology after resection was reported as follicular thyroid carcinoma.

A 53-year-old male patient, who had undergone a goiter operation 10 years ago presented with an anterior mediastinal mass. A fine needle thoracic biopsy revealed papillary thyroid carcinoma, and the mass was excised en-block with collar incision + partial sternotomy (Figure-3).

**Conclusion**

The potential for thyroid cancer in cases of retrosternal goiter is considered to be low, with previously reported rates varying between 5-15%. While operating on a retrosternal goiter confirmed as a thyroid carcinoma, attention must be paid to preserving the recurrent laryngeal nerve and avoiding residual tumor tissue. Additionally a cervical lymph node dissection must be added to surgery. Collar and partial sternotomy incisions are sufficient for the excision in the majority of cases, while a thoracotomy incision may be required in rare cases. Survival may vary, depending on the pathological type of the tumor.

The patients provided written consent to the publication of clinical or personal details.

## P14 Gastroepiploic artery harvesting for CABG - the alternative surgical technique

### Hristo Stoev, Zaprin Vazhev, Konstantin Dimitrov

#### St. George University Hospital– Plovdiv, Department of Cardiac Surgery, Medical University-Plovdiv, Department of Cardiac and Vascular surgery

##### **Correspondence:** Hristo Stoev

We present alternative approach in the harvesting of the right gastroepiploic artery (RGEA), in particularly the surgical technique for performing the opening of the diaphragm in 52 patients who underwent CABG using RGEA.

## P16 15-years of clinical experience in carotid thrombendarterectomy and coronary artery bypass grafting as single staged procedure

### Zaprin Vazhev^1,2^, Konstantin Dimitrov^1,2^, Hristo Stoev^1,2^, Asen Ivanov^1^, Todor Gonovski^1,2^

#### ^1^University Hospital “St.George”-Plovdiv, Bulgaria; ^2^Medical University of Plovdiv, Plovdiv, Bulgaria

##### **Correspondence:** Konstantin Dimitrov

**Background**

The management of concomitant coronary and carotid artery disease is still in evolution. Patients who have severe coronary artery disease, often have carotid stenosis. Although carotid stenting is increasingly performed in this setting, the haemodynamic changes that may accompany this procedure may be harmful to some of the patients.

Patients and Methods: The objectives of this study are to be represented the most typical prognostic factors and clinical manifestations of the patients, who underwent a combined carotid thrombendarterectomy(CEA) and coronary artery bypass grafting(CABG) in our institution .The study was designed as a retrospective study of all patients who underwent cardiac surgery in our institution from October 2002 to December 31, 2017. Data from clinical trials worldwide was also used for the preparation of this study.

**Results**

From October 2002 to December 2017 , we operated on 103 patients performing combined carotid endarterctomy and myocardial revascularization. Short- and long-term results were reviewed.

Conclusion: Our recommendation is that for patients with concomitant carotid and coronary artery disease a combined surgical procedure is indicated , especially if they have life threatening coronary anatomy consisting of LM disease or proximal LAD stenosis or high-grade stenosis three-vessel disease (high SYNTAX score).

**Keywords**

CABG, Carotid endarterectomy, Single staged

## P25 Retrospective long-term analysis of mitral valve surgery for ischaemic mitral regurgitation

### Sophia Mahboob, S. Mohamed, K. Mazhar, R. Warwick, A. Levine, CMRS Satur, Q. Abid, L. Balacurmaraswami, P. Ridley

#### Cardiothoracic Department, Royal Stoke University Hospital, Newcastle

##### **Correspondence:** Sophia Mahboob

**Aim/objectives**

Retrospective single institution analysis of mitral valve surgery for ischaemic mitral regurgitation performed as concomitant, hybrid or independent procedures to analyse NYHA status pre-and post-surgery and intergroup comparison with respect to survival and requirement for re-intervention.

**Methods**

Retrospective analysis of patients from dendrite intellect database and information regarding demographics, pre-operative NYHA status, LV function, operative procedure, post-operative NYHA dyspnoea status, requirement for re-intervention and procedure and long-term survival were parameters that were analysed from individual case records.

**Results**

105 amount of patients fund for the corresponding time period. Number of isolated repairs performed were 1 patient, number of isolated replacements performed were 2 patients, number of replacement valves with cardiac procedures were 26 patients, number of repair valves with other cardiac procedures were 76 patients. Re-intervention within the repair was compared to the replacement group

**Conclusion**

Analysis of the results demonstrates that mitral valve repair can be performed for ischaemic aetiology with favourable results demonstrating low rate of re-intervention and satisfactory and durable repair. Furthermore, long-term survival within this cohort was at least comparable and non-inferior to the replacement group. Although the rate of re-intervention and complication within the replacement group was low, when compared to the repair group was not statistically significant. From our results it can be inferred that mitral valve repair for ischaemic mitral regurgitation can be performed safely with excellent efficacy and comparable long term survival.

## P26 10 - years experience in surgical treatment of acute pulmonary embolism

### Todor Gonovski^1,2^, Zaprin Vazhev^1,2^, Asen Ivanov^2^, Hristo Stoev ^1,2^, Konstantin Dimitrov^1,2^

#### ^1^Department of Cardiovascular Surgery, Medical University, Plovdiv, Bulgaria; ^2^“St. George” University Hospital, Plovdiv, Bulgaria

##### **Correspondence:** Todor Gonovski

**Background**

Acute massive and submassive pulmonary embolism is a life threatening condition with high mortality rate. This study analyze the data from a single center experience of pulmonary embolism which include rapid diagnosis with contrast – enhanced chest computed tomography as well as echocardiography to document right ventricular strain followed by surgical intervention.

**Materials and methods**

Between the time period from January 2008 to December 2018, 57 patients (35 men and 22 women) underwent urgent surgery for acute pulmonary embolism. Mean age was 62 years with age range from 36 to 84 years. Indications for surgical intervention were: right ventricular dysfunction, severe dyspnea, cardiogenic shock and cardiac arrest due to PE.

**Results**

In-hospital mortality rate was 14% or 8 patients. One of them died on postoperative day 3 after recurrent PE. In five cases the main cause of death was severe right ventricular dysfunction and heart failure. Other two patients died due to neurological complications. ECMO was used perioperative in 3 (5,2%) cases. Mean in-hospital stay was 14 days ( range 7 – 35 days). Survival rate at 1 year follow-up was 81%.

**Conclusions**

Surgical pulmonary embolectomy should be a treatment of first choice in patients with massive and submassive acute pulmonary embolism with a reasonable mortality and morbidity rates.

Keywords: Surgical embolectomy, pulmonary embolism, ECMO.

## P28 Sternal wound infections following cardiac surgery: our experience a single centre study

### Sujeeth Suvarna, Joanne Flanagan, Fiona O Rourke, Maria Hayes, Delia Clune, Mary O Gorman, Edel Costigan

#### Blackrock Clinic, Blackrock, County Dublin, Ireland

##### **Correspondence:** Sujeeth Suvarna

**Background**

Sternal wound infection (SWI) remains a major concern in cardiac surgery despite many advances. Our aim was to evaluate the pathogens involved in sternal wound infections following cardiac surgery especially in a population with increasing age and co-morbidity factors.

**Methods**

A retrospective analysis from April 2018 to March 2019 of 328 patients undergoing cardiac surgery was carried out. These were performed by 5 surgeons at Blackrock clinic, Dubin, Ireland.The variables (Demography, Co-morbid factors, Pre, Peri and Post-operative factors), Swabs for Superficial and Deep SWI were analysed for microorganisms during their hospital stay.

**Results**

The 12-month analysis showed 7 out of 328 (2.13%) were diagnosed with SWI. Superficial SWI n=3(0.9%), Deep SWI n=4(1.2%). Male n=6, Female n=1

Age years range

No of Patients

50-59

1

60-69

1

70-79

5

Sex

M-6, F-1

BMI

<30

4

>30

3

Diabetic

NIDDM-3

Smoker

1 (Ex-smoker-6)

Previous Cardiac Surgery

1 (Valve)

Cardiac Procedures

Elective-4, Urgent-3

CABG

3

Valve

1

CABG+Valve+Other

1

Other

2

Patient Status at Discharge

Alive-6, Dead-1

SWI Classification & Causative Organism

No. of Patients

Deep Sternal SWI

4

Enterobacter Cloacae

1

Klebsiella pneumoniae

1

Protius Mirabilis

1

Staphylococcus Epidermidis

1

Superficial Sternal SWI

3

Staphylococcus Epidermidis

2

No Growth

1

**Conclusion**

Our analysis of microorganisms following SWI were mainly commensals and the most common pathogens being Staphylococcus epidermidis.A review of the literature suggests that with proper antimicrobial prophylaxis the incidence of SWI can be kept at a minimum.

## P30 Surgically treated saccular left main coronary artery aneurysm

### Asen Ivanov^1,2^, Zaprin Vazhev^1,2^, Todor Gonovski^1,2^, Hristo Stoev^1,2^, Konstantin Dimitrov^1,2^, Gencho Nachev^3^

#### ^1^Department of Cardiovascular Surgery, Medical University, Plovdiv, Bulgaria; ^2^“St. George” University Hospital, Plovdiv, Bulgaria; ^3^“St. Ekaterina” University Hospital, Sofia, Bulgaria

##### **Correspondence:** Asen Ivanov

Coronary artery aneurysm is not a common diagnosis, and those of the left main coronary artery(LMCA) are extremely rare with an incidence of 0.1% of patients undergoing percutaneous coronary intervention(PCI). We report a case of 68-year-old male patient, hospitalized in interventional cardiology department with a retrosternal pain, where PCI was performed revealing a saccular aneurysm of the LMCA. Computed tomography(CT) scan confirmed the diagnosis of isolated coronary artery aneurysm 15mm distal to the orifice of the LMCA. Cardiac surgery procedure was performed including: double coronary artery bypass grafting(CABG), occlusion of the LMCA orifice combined with distal occlusion of the aneurysm. The postoperative course was uneventful and the patient was discharged on postoperative day 6 without any remarks. Even though the etiology of the aneurysm was not fully investigated, it was suspected to be a congenital one.

**Keywords**

Left main coronary artery aneurysm, coronary artery disease, coronary artery bypass grafting.

## P33 Long-term results after adrenalectomy for isolated adrenal metstases in operable patients with nsclc- two institutions study

### Anatoli Semkov^1^, Georgi Yankov^1^, Emilia Naseva^3^, Dejan Djuric^2^, Danail Petrov^1^

#### ^1^Thoracic Surgery department, MHATPD “St. Sophia”, Medical University Sofia, Bulgaria; ^2^Thoracic Surgery Department, Institute for lung diseases, Novi Sad, Serbia; ^3^Faculty of Public Health, Medical University Sofia, Bulgaria

##### **Correspondence:** Anatoli Semkov

**Background**

Isolated adrenal metastasis (IAM) from non-small cell lung cancer (NSCLC) is a rare event and the management in such patients is still under discussion.

**Objective**

To evaluate the long-term results after surgery of IAM in radically resectable NSCLC as a part of multidisciplinary approach.

**Methods**

Eleven patients (mean age 58.4 years) underwent adrenalectomy for NSCLC IAM. IAMs were synchronous (6) and metachronous (5), 4 of them were contralateral and 7 ipsilateral. Locoregional pStage was I-II in 10 patients and IIIA in 1 patient. One stage left lower lobectomy, phrenotomy and ipsilateral adrenalectomy was performed in 1 patient. In 9 cases the radical lung resection was done first, followed by adrenalectomy via paracostal laparotomy (3), transperitoneal laparoscopy (1) and retroperitoneal endoscopic adrenalectomy (REA) - 6 patients. In the last case REA was performed first, followed by right lower lobectomy. Two years later, metachronous ipsilateral adrenal and contralateral lung metastases were eradicated by REA (first stage) and right polysegmentectomy S7-S10 (second stage). All patients were followed up either to the end of the study or to their death (mean interval of 54 months). The survival was studied by Kaplan-Meier method.

**Results**

No perioperative death was observed. Three patients are still alive until the last follow up.The mean overall survival (OS) time is 44.8 months. One- and 3-years OS rate is 90.9% and 64.6%, respectively. Mean progression free survival (PFS) time is 29.3 months. One-year and 2-years PFS rate is 80.0% and 40%. There is no significant differencesin median OS and median PSF time between synchronous vs metachronous IAM (p=0.914, CI 95%), ipsilateral vs contralateral IAM (p=0.244; CI 95%) and laparoscopic vs conventional adrenalectomy (p=0.163; p=0.754).

**Conclusions**

Long term survival is possible after resection of IAM in strictly selected NSCLC patients with early locoregional stage.

## P34 Experimental studies of methylene blue in the treatment of hemorrhagic shock in pigs

### Paulo Roberto Evora, Agnes Albuquerque, Andrea Celotto, Christiane Becari, Marelaine Prandi, Jessyca Barbosa

#### Department of Surgery and Anatomy, Ribeirão Preto Medical School, University of São Paulo, SP, Brazil

##### **Correspondence:** Paulo Roberto Evora

**Objective**

Hemorrhagic shock is an event many times fatal in cardiac surgery. This investigation was carried out to verify if intravenous methylene blue (MB) prevents and reverses the induced hemorrhagic shock in pigs.

**Method**

The following vessels were catheterized: right femoral artery and vein (volume replacement only with autologous blood), left jugular vein (continuous infusion of anaesthesia), right jugular vein (Swan-Ganz catheter for continuous measurement of cardiac output) and left carotid artery (continuous mean arterial pressure (MAP) recording). The experimental model adopted was “fixed pressure haemorrhage” (MAP 50 - 55 mmHg and cardiac index decrease). The protocols were:1) 1h BR: the animals received blood replacement 1 hour aftershock; 2) 1h MB: MB bolus injection (2mg/kg) 1 hour aftershock; 3) 1h MB+BR: MB bolus injection of MB and blood replacement 1 hour aftershock; 4) 15 min MB+BR: bolus injection of MB and blood replacement 15 minutes after shock; 5) 15 BR+ 1h MB: blood replacement 15 minutes after shock and MB bolus injection MB 1 hour aftershock; 6) 15 MB+ 1h BR: MB bolus injection 15 minutes after shock and blood replacement 1 hour aftershock.

**Results**

The biochemical and blood gases changes were not relevant considering the experimental time. Among all the observed hemodynamic parameters, mean arterial pressure (MAP), mean pulmonary arterial pressure (MPAP), pulmonary capillary pressure (PCP), central venous pressure (CVP), cardiac output (CO), cardiac index (CI), systemic vascular resistance (SVR), pulmonary vascular resistance (PVR), in general, showed improvement only with BR or MB + BR, but only the 1hMB + BR group had a better final MPAP and PVR.

**Conclusions**

MB should be useful as a temporizing measure for resuscitation after refractory hemorrhagic shock and warrants further study for its application to hemorrhagic patients

**Keywords**

Hemorrhagic shock, methylene blue, blood replacement

## P35 Standardization of an experimental model of chronic metabolic acidosis in rats

### Paulo Roberto Evora, Agnes Albuquerque, Willian Márcio da Silva, Luiza Mateus, Andrea Celotto

#### Department of Surgery and Anatomy, Ribeirão Preto Medical School, University of São Paulo, SP, Brazil

##### **Correspondence:** Paulo Roberto Evora

**Introduction**

The acid-base disorders are common in the medical practice and can vary from acidosis or pure alkalosis to a mixed, complicated and potentially fatal complication. The methodology to induce chronic metabolic acidosis in rats is not a simple activity needing specific technical details.

**Objectives**

To develop an efficient and reproducible model of chronic metabolic acidosis (CMA) in rats.

**Methodology**

CMA was induced using two protocols: 1) oral administration (ad libitum) of NH4Cl 0,5M dissolved in a powdered juice solution (acidosis 1) for ten days or; 2) oral administration (ad libitum) of NH4Cl 0,5M dissolved in a powdered juice solution associated with gavages of NH4Cl0,02M in water, for ten days (Acidosis 2). The treatment aimed to reach a pH close to 7.2.

**Results**

CMA induced by ammonium chloride (NH4Cl) reduced the pH to 7.17 (7.39 control), with levels of bicarbonate (HCO3-) about 9.8 mmol/L (21.9 control mmol/L). Animals that received NH4Cl also had lower weight gain during the study period. This reduction in weight gain could be due to a decrease in feed intake observed in these animals. The CMA induced by NH4Cl through gavage infusions, and association of powdered juice, was more efficient than ad libitumoral administration.

**Conclusions**

The experimental CMA model was reproducible, emphasizing that the association of powdered juice and gavage were excellent resources for the acceptance of the acid solution by the rats and allowing better results.

**Keywords**

Chronic metabolic acidosis, ammonium chloride, acid-base balance

## P36 Right lower bilobectomy for two synchronous lung neoplasm after minimally invasive aortic arch debranching: a multidisciplinary team success

### Fabio Davoli^1^, Alberto Tripodi^2^, Giorgio Grani^3^, Maurizio Salvi^3^, Paolo Bagioni^1^, Guido Caroli^1^, Riccardo Milani^4^, Ubaldo Turicchia^4^, Giulio Rossi^5^, Gianluca Danesi^6^, Manolo D’Arcagnelo^7^, Massimo Terenzoni^8^, Maurizio Fusari^8^, Domenico Palmarini^9^ and Franco Stella^10^

#### ^1^Department of Thoracic Surgery, AUSL Romagna, S. Maria delle Croci Teaching Hospital of Ravenna, Italy; ^2^Department of Cardiac Surgery, Villa Maria Cecilia Hospital of Cotignola, Italy; ^3^Department of Thoracic Surgery, AUSL Romagna, Ceccarini Hospital of Riccione, Italy; ^4^Department of Vascular Surgery, AUSL Romagna, S. Maria delle Croci Teaching Hospital of Ravenna, Italy; ^5^Department of Pathology, AUSL Romagna, S. Maria delle Croci Teaching Hospital of Ravenna, Italy; ^6^Department of Thoracic Disease, AUSL Romagna, S. Maria delle Croci Teaching Hospital of Ravenna, Italy; ^7^Department of Oncology, AUSL Romagna, S. Maria delle Croci Teaching Hospital of Ravenna, Italy; ^8^Department of Anesthesiology, AUSL Romagna, S. Maria delle Croci Teaching Hospital of Ravenna, Italy; ^9^Department of Radiology, AUSL Romagna, S. Maria delle Croci Teaching Hospital of Ravenna, Italy; ^10^Department of Thoracic Surgery, AUSL Romagna; Director of the School of Specialization of Thoracic Surgery, University of Bologna, Italy

##### **Correspondence:** Fabio Davoli

**Background**

To report the case of the successful surgical treatment of two synchronous lung neoplasm occasionally found in a patient with a damaged stent of the brachiocephalic trunk.

Case Report.

A patient was referred to our Thoracic Surgical Department with a diagnosis of a cT4N1 Adenocarcinoma of the lung for two masses located, respectively, at the hilum of the middle lobe and at the superior segment of the right lower lobe. The patient underwent a brachiocephalic trunk stenting two years before and the two lung tumours were found accidentally during the vascular surgery follow-up.

The angiography revealed the stent fractured conditioning a severe stenosis of both the brachiocephalic trunk than the common carotid artery; an endovascular repair was excluded for the high risk of unsuccess. The patient was symptomatic for syncope and the anaesthesiology team didn’t allow pulmonary resection at that time. On the other hand a neoadjuvant chemotherapy wasn’t recommended for comorbidities.

After multidisciplinary team (MDT) evaluation the patient was referred to the Cardiac Surgery Team and underwent an aortic arch debranching by a minimally invasive approach, consisting in a manubriotomy plus two centimetric cervical accesses. One month after Cardiac Surgery a Total Body Computed Tomography Scan was accomplished, showing no oncologic progression of disease.

Finally we performed a successful right lower bilobectomy with a systematic lymphadenectomy by a posterior-lateral muscle-sparing thoracotomy. Although a right pneumonectomy was avoided, the patient had a slow recovery and the post-operative period was complicated by a nosocomial pneumonia and the patient was discharged in post-operative day 18.

Histopathological examination disconfirmed the clinical stage showing two different neoplasm: a 4,5 cm pT2N0 poorly differentiated squamous cell carcinoma of the the right lower lobe and a 3,3 cm pT2N1 predominantly acinar adenocarcinoma of the middle lobe. Adjuvant therapies were excluded for comorbidities and a strict radiological four months follow-up was set up.

**Conclusion**

MDT evaluation and strictly collaboration between a wide range of Specialists can provide the best treatment for such complex cases like this report.

## P37 Reduced stay in high dependency unit for heart failure patients following out-patient iron replacement therapy: Our single centre experience

### Norma Caples, E. Cronin, C. Cawley, C. O'connor, J. Kumar, S. Asgedom, P. Owens

#### University Hospital Waterford, cardiology, Waterford, Ireland

##### **Correspondence:** Norma Caples

**Background**

In Ireland forty seven percent of total direct costs are related to heart failure care and heart failure accounts for seven percent of all inpatient beds. Despite international trial data demonstrating reduced rates of readmission and improved NYHA focused on the benefits of treating iron deficient patients with intravenous iron no previous study evaluated its effects on length of hospital stay and need for admission to high dependency unit within the Irish South-Eastern Health Board Heart Failure Service (SEHB-HFS) population. We therefore proceeded to conduct this single centre study by optimizing patients’ clinical condition in the out-patient setting, with intravenous iron replacement.

**Materials & Methods**

The study was designed as a self-controlled case series examining patients with systolic heart failure, iron-deficiency (ID) and anaemia. This include evaluation both at enrolment to study and following IV iron replacement over a follow-up period of 6 months at either tail. Data was collected from the SEHB-HFS database, providing in-depth data for each patient including: admission rate and course of admission during hospital stay. Iron replacement was conducted in accordance with the ESC guidelines for heart failure. Wilcoxon Sign Rank Testing was used as a basis for statistical analysis.

**Results**

Of the 178 patients included in the SEHB-HFS database, a total of 57 were found to have ID. Readmission rates were reduced in those patients who were treated with intravenous iron replacement (2.29 vs 1.00 p<0.005). In addition, the length of hospital stay had dropped significantly (7.3 days vs. 5.3 days p<0.05). The average admission to high dependency areas were reduced in the group who were treated with iv iron (0.66 days v 0.33 days, p < 0.05).

**Conclusion**

Our study demonstrate a significant reduction in length of hospital stay and admission to high dependency unit of heart failure patients with concomitant iron deficiency when treated with IV Iron supplement in a day care setting. This may be a refection in improvement in their general well being when their ID is treated, thus reducing the cost of hospital care.

## P38 Modified bt-shunt in surabaya, indonesia: patient characteristics and incidence of shunt thrombosis

### Yuletta Ambarsari, Arief Hakim, Heroe Soebroto

#### Departement of Cardiac, Thoracic and Vascular Surgery, Medicine Faculty of Airlangga University, Surabaya Indonesia

##### **Correspondence:** Yuletta Ambarsari

**Background**

Modified Blalock-Taussig shunt (modified BT-Shunt) is one of palliative heart surgery to increase pulmonary blood flow in case of children with ductal dependent or decreased pulmonary blood flow. This study aimed to review modified BT-Shunt patient characteristics and incidence of shunt thrombosis in the single-centre experience.

**Materials and Methods**

Eighty two patients between 2012 and 2017 were retrospectively reviewed. Inclusion criteria was all patient who underwent modified BT-shunt using polytetrafluoroethylene (PTFE) shunt as an initial palliative surgical treatment. Follow-up was accomplished using routine post operative echocardiography. Shunt patency was defined as an open graft until patient discharged. The association between patient characteristics and shunt thrombosis were calculated using Logistic Regression method.

**Results**

Forty three patients (52%) were female. Median age was 15 (range 1-252) months. Median weight and hemoglobin were 7.9 (range 2.8-75) kg and 11.7 (range 9.5-26) g/dL. Twenty six (31%) patients were planned for univentricular repair and 56 (69%) patients underwent biventricular repair. The sizes of shunt were 3 mm in 1 (1%) patient, 3.5 mm in 5 (7%) patients, 4 mm in 32 (39%) patients, 5 mm in 43 (52%) patients and 10 mm in 1 (1%) patient, respectively. Patients underwent modified BT-Shunt surgery with anterior sternotomy approach in 66 (80%) patients.

Shunt thrombosis occurred in 4 (5%) patients, 3 of them were female. All patients with shunt thrombosis were planned for future biventricular repair.

Median age, weight and hemoglobin were 96 (range 36-180) months, 22.5 (range 8.6-75) kg and 10.8 (range 21.6-22.8) g/dL, respectively. Of these variables, only two showed a statistically significant association with shunt thrombosis: hemoglobin (22.20 g/dL ± 0.84 with thrombosis versus 17.55 g/dL ± 4.65 no thrombosis; p = 0.018) and patients weight (32.15 kg ± 31.03 with thrombosis versus 8.55 kg ± 6.42 no thrombosis; p = 0.013).

The mean age was 102 months ± 76 with thrombosis versus 27 months ± 39 no thrombosis (p = 0.617).

Shunt sizes in patients with shunt thrombosis were 5 mm 3 patients and 10 in 1 patient. Shunt diameter showed no significant association with shunt thrombosis (6.25 mm ± 2.5 with thrombosis versus 4.47 mm ± 0.56 no thrombosis; p = 0.213).

Only 1 patient with shunt thrombosis underwent operation with thoracotomy approach. There was no significant difference in incidence of shunt thrombosis in patients who underwent operation with thoracotomy approach or without thoracotomy approach (25 ± 50% versus 19 ± 40%; p = 0.73).

The patients with shunt thrombosis who were operated without heart lung machine or with heart lung machine, had no significant difference in incidence of shunt thrombosis (25 ± 50% versus 8 ± 26%; p = 0.21)

**Conclusions**

The use of modified BT-Shunt in our center had a good result with less incidence of thrombosis.

**Keywords**

modified BT-Shunt, thrombosis, Indonesia

## P39 Needlescopic video-assisted thoracic surgery for pulmonary nodules and mediastinal tumors

### Makoto Oda, Koujiro Shiga, Hideaki Miyamoto

#### Department of Thoracic Surgery, Shin-yurigaoka General Hospital, Kawasaki, Kanagawa, Japan

##### **Correspondence:** Makoto Oda

**Background**

Thoracic surgeons’ concerns are toward to further less invasive video-assisted thoracic surgery (VATS) such as uniportal VATS. We evaluated our experience with novel needlescopic VATS wedge resection combined with subcostal trans-diaphragmatic (SCTD) approach for managing undetermined peripheral pulmonary nodules and with needlescopic VATS resection for mediastinal tumors.

**Methods**

Between September 2017 and March 2019, 21 patients underwent needlescopic VATS wedge pulmonary resection with SCTD approach and 6 patients underwent needlescopic VATS thymectomy for anterior mediastinal tumors. For undetermined pulmonary nodules, preoperative percutaneous computed-tomography guided marking of the nodule was performed. 1) Pulmonary nodules: Two 3-mm mini-ports were placed in the thorax for the thoracoscopic camera and mini-grasper. Just anterior of the tenth rib, a 2-cm subcostal incision was made and a 12-mm or 15-mm port was placed trans-diaphragmatically into the chest cavity. Wedge resection of the lung was performed with endostaplers introduced through a subcostal port. 2) Mediastinal tumors: One 5-mm port for energy device and two 3-mm mini-ports for the thoracoscopic camera and mini-grasper were placed.

**Results**

There was no mortality and no hospital death. None required conversion to a thoracotomy, and 1 patient with mediastinal tumor required conversion of one 3-mm port to 5-mm port. No diaphragmatic hernia was observed. The duration of chest tube drainage was no-tube in 25, 0 day in 1, 1 day in 1. Postoperative hospital stay was 1 day in 11 and 2 days in 16. The pathological diagnoses were lung cancer in 19 patients, other benign pulmonary nodule in 2, and thymic cyst in 6.

**Conclusions**

Our experience showed that needlescopic VATS wedge pulmonary resection combined with SCTD approach for pulmonary nodules and needlescopic VATS resection for mediastinal tumors are a safe and feasible procedure which offers specific advantages with minimally invasive and cosmetic outcomes.

## P40 Hybrid approach in the treatment of postcoarcation aneurysms

### I.Kravchenko, V. Kravchenko, B. Cherpak, Yuri Tarasenko, I. Ditkivskyy, V. Lazorishinets

#### M. M. Amosov Institute

##### **Correspondence:** Yuri Tarasenko

**Objective**

Coarctation of aorta (CoA) accounts for approximately 7% of congenital heart lesions. Most widespread method of correcting of CoA is surgery. In remote period there is a high risk of aneurysm formation.

AIM. Analysis the reason and frequency. Methods and results of treatment of aneurysms at the site of CoA correction.

**Materials and methods**

4245 patients with CoA were operated in the Institute during 1960-2017. Aneurysms after CoA repair developed in 184 (4.3%) patients (pts) in period from 1 months to 42 years. Patients age at first operation - 1-38 yy, average 17,1y; at the second one – 9-68 yy, average 32,5 yy. Mean term from CoA repair to postcoarctation aneurysm correction composed 15,4 years.

Aneurysms developed in 16 (0.6%) pts after CoA repair by end-to-end anastomosis, in 18 (5.9%) patients after aorta grafting and in 132 (10.9%) after patch aortoplasty.

Aneurysm diagnosis was established by X-ray, TT/TEEcho and CT.

150 (81.5%) patients were operated, among them 134 were operated by open surgery and 16 – by endovascular approach. Among 134pts, 14 pts were operated two times and 4 pts - three times. 34 (18.5%) of them abstained from surgery for various reasons (26 patients died during 5 years after conformation diagnosis).

Visceral organs protection during redo surgery in 123 (91.3%) patients was performed by passive shunt between ascending and descending aorta, in 9 (3.8%) patients left atrium-descending aortic bypass was used, “clamp and sew” technique – 2 pts. After aneurysms resection, graft replacement (131 pts-90.0%) was the main method, aortorrhaphy - in 3 (2.0%), TEVAR - 16 (8.0%) pts.

In 5 cases we had isolated TEVAR and in 11 – hybrid approach. Patients scheduled for elective surgery had debranching of aortic arch at the first stage and TEVAR at the second stage. If they had life-threatening conditions they underwent TEVAR immediately; then after their condition improved, open surgery stage was conducted. In 8 cases, patients had partial debranching, in 2 – subtotal, in 1 - total. We implanted 28 devices in 16 patients. In 3 cases we used scallop stent grafts and in 5 - physician modified stent grafts (self-made fenestration) to revascularize aortic branches.

**Results**

Hospital mortality composed 14 (9.3%). There were no renal and spinal cord complications.

**Conclusions**

1. The highest rate of complications in remote period is after the method of patch plasty (10,9%). 2. Grafting (open surgery or TEVAR) the aneurism at the site of CoA correction is a life-saving procedure. 3. To reveal complications in time at follow-up period lifelong observation of operated

## P41 Acute heart failure peripartum and after delivery

### Lea Ivanovska^1^, Lidija Jovcevska Ivanovska^2^

#### ^1^UKC Ljubljana, Slovenia; ^2^Obstetric Ward, General Hospital, Kumanovo, Republic North Macedonia

##### **Correspondence:** Lidija Jovcevska Ivanovska

Аccording to various authors, on every 7000-10000 births, 1 woman can develop acute heart failure. This term, peripartum cardiomyopathy, is used for patients who develop acute heart disorders during childbirth or short-term postpartum.

**Aim**

Aim of this article is to present 7 cases of peripartum acute cardiac failure.

**Materials and methods**

Histories of diseases of treated patients have been used, laboratory findings, examinations and EKG, RTG findings from the internist. The patients are 27 to 34 years of age. They were pregnant between 35-37 g.w. 3 patients were pregnant for the first time. 3 are pregnant for the second time.1 was a twin pregnancy. 4 patients are obese and have added more than 15 kg in pregnancy. All of them had occasionally elevated blood pressure in the last 4 weeks in pregnancy (more than 130/90). 6 patients come to the department due to dyspnoea, orthopnea, cough, weakness, tachycardia. A pregnant woman with twins, all symptoms appear in the first 12 hours after delivery. From clinical and hospital trials to mark is a mild to severe anemia among all 7 pregnant women - Hgb 98-111, as well as hypoproteinaemia. Echocardiography and EКG were made. The hallmark finding is cardiomegaly. After working diagnosis, acute heart failure, therapy is digoxin, anticoagulant, diuretics. Antibiotic was given to all patients. They are monitored all the time and the fetus also. When patients are stabilized, they are prepared for delivery and it was with a Caesarean Section. The postoperative course is within the normal range. In 3 patients, cardiomegaly recedes after 6-12 months; in 2 patients, permanent damage to the cardiac muscle remains. 2 patients were transferred to the Cardiology Clinic for unstable heart failure.

**Conclusion**

Peripartum heart failure is rare but very dangerous, frightening condition in obstetric.

## P42 Will combination of CT FFR coronary angiogram and adjunct software replace invasive diagnostic coronary angiography as a diagnostic modality?

### Sophia Mahboob, S. Mohamed, K. Mazhar, L. Balacumaraswami, S. Duckett

#### Heart and Lung Department Royal Stoke University Hospital, Newcastle

##### **Correspondence:** Sophia Mahboob

**Aim/objectives**

Fractional flow reserve (FFR) CT coronary angiogram is an evolving area of interest with respect to evaluating coronary artery disease and assessment of haemodynamically significant disease to reliably produce measurements comparable to invasive diagnostic coronary angiography and catheter based pressure wire measurements.

**Methods**

Retrospective single institution analysis of patients who underwent CT coronary angiogram and CT derived FFR measurements for assessment of haemodynamically significant disease in the coronary arteries. Identified patients then progressed towards diagnostic invasive coronary angiography for correlation. These were performed in the elective setting for patients seen through rapid access chest pain clinic or outpatient clinic setting. The FFT-derived CT guided data was combined with HeartFlow software to produce a 3D model of the coronary arteries and analyse the effect of the stenoses on blood flow.

**Results**

284 patients had undergone FFR-derived CT coronary angiogram. The patients who were identified to have important haemodynamically significant stenoses progressed to diagnostic invasive coronary angiogram for correlation to establish a concurrence rate.

**Conclusion**

Analysis of the results demonstrates that FFR-derived CT coronary angiogram can reliably establish haemodynamically significant coronary artery disease and a high degree of concurrency. Furthermore, the potential of the application in combination with software such as HeartFlow can produce personalised 3D models which can then be used to corroborate the lesions on invasive coronary angiography with a view to percutaneous intervention or even towards intervention in the form of surgery if indicated.

## P43 Hybrid approach: remote endarterectomy and endovascular recanalization in a patient with chronic upper extremity arterial occlusion. Case report

### Alexander T Daskalov, Nikolai Valchev, Vassil Chervenkov

#### Vascular surgery department, Acibadem City Clinic Tokuda Hospital, Sofia, Bulgaria

##### **Correspondence:** Alexander T Daskalov

**Aim**

We want to present a rare case of remote endarterectomy of the brachial artery, followed by endovascular recanalization of axillary and subclavian artery, performed in a patient with critical upper limb ischemia, due to chronic atherosclerotic disease. The aim of this case report is to show that this hybrid approach is a feasible option when treating complex multilevel arterial occlusive disease even in the upper extremity.

**Methods**

We report the case of a 66-year-old male with a critical upper limb ischemia, who underwent remote endarterectomy of the brachial artery and subsequent recanalization of the subclavian and axillary artery followed by stent placement.

**Results**

The patient was discharged 2 days after the intervention with palpable distal pulses and no signs of upper limb ischemia. He was followed two months later by a CT angiography showing patent reconstruction. To our knowledge there are only few cases reported in the literature of hybrid vascular reconstruction in the upper limb due to atherosclerosis.

**Conclusion**

We believe the combination of open and endovascular surgical techniques is preferable in carefully selected cases and should be included in the algorithm of treating upper extremity chronic atherosclerotic disease.

**Keyword**

Upper limb, atherosclerosis, ischemia, remote endarterectomy, endovascular recanalization.

**References**

1. Blaisdell FW, Steele M, Allen RE. Management of acute lower extremity arterial ischemia due to embolism and thrombosis. Surgery 1978; 84:822–834.

2. Both M, Jahnke T, Reinhold-Keller E, Reuter M, Grimm J, Biederer J, Brossmann J, Gross WL, Heller M, MuellerHuelsbeck S. Percutaneous management of occlusive arterial disease associated with vasculitis: a single center experience. Cardiovasc Intervent Radiol 2003;26:19 –26

3. Christos Argyriou1 , George S Georgiadis1 , Michael Mantatzis2 , Nikolaos G Schoretsanitis1 , George A Antoniou1 , Maria Z Papadopoulou3 , Theodosia Vogiatzaki3 and Miltos K Lazarides1-Hybrid reconstruction of the upper limb in a patient with chronic limb ischemia. Sage Journals Volume: 23 issue: 6, page(s): 653-656

4. Ciocca RG, Madson DL, Wilkerson DK, Graham AM. Fibromuscular dysplasia of the brachial artery: an endovascular approach. Am Surg 1995;61:161–164.

5. Gross WS, Flanigan P, Kraft RO, Stanley JC. Chronic upper extremity arterial insufficiency. Etiology, manifestations, and operative management. Arch Surg 1978;113:419 –42316.

6. Jivegard L, Holm J, Schersten T. Acute limb ischemia due to arterial embolism or thrombosis: in fluence of limb ischemia versus pre-existing cardiac disease on postoperative mortality rate. J Cardiovasc Surg 1988;29:32–36.

7.Lazarides MK, Staramos DN, Panagopoulos GN, Tzilalis VD, Eleftheriou GJ, Dayantas JN, Staamos DN. Indications for surgical treatment of angioaccess-induced arterial steal. J Am Coll Surg 1998;187:422–426.

8. Levine MP. The hemodialysis patient and hand amputation. Am J Nephrol 2001;21:498 –501.

9. Morsy AH, Kulbaski M, Chen C, Isiklar H, Lumsden AB. Incidence and characteristics of patients with hand ischemia after a hemodialysis access procedure. J Surg Res 1998;7

10. Motarjeme A, Gordon GI. Percutaneous transluminal angioplasty of the brachiocephalic vessels: guidelines for therapy. Int Angiol 1993;12:260 –269

11. Schmidt FE, Hewitt RL. Severe upper limb ischemia. Arch Surg 1980;115:1188 –11

12. SurenaNamdari, MD, Min Jung Park, MMSc, Arnold-Peter C. Weiss, MD, Wilfred I. Carney, MD-Chronic Hand Ischemia Treated With Radial Artery Balloon Angioplasty: Case Report. J Hand Surg Am. 2008 Apr;33(4):551-4. doi: 10.1016/j.jhsa.2007.12.021

13. Takach TJ, Duncan JM, Livesay JJ, Krajcer Z, Cervera RD, Gregoric ID, et al. Brachiocephalic reconstruction II: operative and endovascular management of single-vessel disease. J Vasc Surg 2005;42:55–61.

14. Whelan TJ Jr. Management of vascular disease of the upper extremity. Surg Clin North Am 1982;62:373–389.

15. Yeager RA, Moneta GL, Edwards JM, Landry GJ, Taylor LM Jr, McConnell DB, Porter JM. Relationship of hemodialysis access to finger gangrene in patients with endstage renal disease. J Vasc Surg 2002;36:245–249.

Written informed consent for publication of their clinical details and/or clinical images was obtained from the patient.

## P44 Acquired tracheoesophageal fistula after button battery ingestion in 11-month-old patient

### Zdravka Antonova^1^, Hristo Shivachev^1^, Yanko Pahnev^1^, Nadezhda Tolekova^1^, Velichka Oparanova^1^, Vania Kisimova^1^, Svetlana Velizarova^3^, Natalia Gabrovska^3^, Alexander Tcherveniakov^2^

#### ^1^Pediatric Surgery, UMHATEM “N. I. Pirogov”, Sofia, Bulgaria; ^2^Thoracic Surgery, UMHATEM “N. I. Pirogov”, Sofia, Bulgaria; ^3^MHATPD “Sveta Sofia”, Sofia, Bulgaria

##### **Correspondence:** Zdravka Antonova

**Introduction**

Button battery swallowing is increasing its incidence in the pediatric population. Esophageal impaction of such foreign bodies can lead to severe and even fatal complications, regardless of their timely extraction.

**Material and methods**

An 11-month-old boy was admitted in our hospital six days after button battery extraction from the upper third of the esophagus. The extraction took place in another hospital and was performed within 6-8h after the incident. At the time of hospitalization, the child showed symptoms of corrosive esophagitis and mediastinitis. An esophagoscopy was performed and conservative treatment started in ICU. Sixteen days after the incident a tracheo-esophageal fistula was formed, confirmed with CT scan and bronchoscopy. An operation with partial esophageal resection and tracheal suturing was performed via cervical incision.

**Results**

On day 8 the patient developed an esophago-cutaneous fistula in the cervical area, which resolved spontaneously in one month. A mild esophageal stricture, that required several balloon dilations in the next 8 months, was formed. The most recent X-ray investigation showed no esophageal dysfunction. The patient shows no feeding or respiratory problems as a result of the incident or the operation.

**Conclusion**

The widespread use of button batteries in consumer electronics and curiosity in children lead to incidents that may evolve into a crippling and even fatal pathology. The acquired tracheo-esophageal fistula in the upper third of the esophagus is a rare complication after battery ingestion and can be treated successfully with cervical access only. Non-severe complications can be managed conservatively.

Written informed consent for publication of their clinical details and/or clinical images was obtained from the parent of the patient.

## P45 Circular tracheal stenosis after intubation in 3-year-old

### Zdravka Antonova^1^, Hristo Shivachev^1^, Tsvetan Minchev^2^, Yanko Pahnev^1^, Nadezhda Tolekova^1^, Velichka Oparanova^1^, Vania Kisimova^1^, Svetlana Velizarova^3^, Natalia Gabrovska^3^

#### ^1^Pediatric Surgery, UMHATEM “N. I. Pirogov”, Sofia, Bulgaria; ^2^Department of Thoracic Surgery, Acibadem City Clinic Tokuda Hospital, Sofia, Bulgaria; ^3^MHATPD “Sveta Sofia”, Sofia, Bulgaria

**Introduction**

Tracheal stenoses are one of the most common complications after prolonged endotracheal intubation. They develop most often at the balloon level and become symptomatic in 4 to 8 weeks after extubation. Some strictures however develop even after a short intubation time.

**Material and Methods**

We present a case of a 3 year old girl with recurrent respiratory infections in the past. The patient was admitted in our clinic with dyspnoea and computer tomography data for a high grade circular tracheal stenosis 1.5-2 cm proximal to the carina, with a residual lumen width of 5 mm. According to the medical records two months earlier due to bilateral pneumonia and respiratory failure, the child was intubated for 72 hours. The intubation tube’s parameters were adequate for age and body mass. Transsternal transpericardial approach was used and resection of four tracheal rings was performed. The anastomosis was made with running sutures. Anesthesia was carried out by orotracheal and intrabronchial intubation. The patient was extubated immediately after the end of the surgical intervention.

**Results**

The bronchoscopies on 7th and 11th postoperative days did not reveal anastomotic insufficiency. There was a wound infection in the cervical area that was managed conservatively. The patient shows no symptoms of respiratory insufficiency.

**Conclusion**

The trachea is a sensitive and fragile organ. Even small lesions and short-term interventions can lead to severe complications that need extensive surgery. Non-traumatic intubation and caring attitude to the tracheo-bronchial tree are the best method of preventing complications such as post-intubation stenoses. The transsternal transpericardial approach gives complete and safe access to the trachea, main bronchi and large pulmonary vessels.

Written informed consent for publication of their clinical details and/or clinical images was obtained from the parent of the patient.

## P48 Spontaneous rib fractures analyses our cases

### Fadil Gradica, L. Lisha, Dh. Argjiri, A. Cani, F. Kokici, Z. Perduka, J. Beli,K. Gradica, A. Lala

#### University Hospital “Shefqet Ndroqi”, Tirana, Albania

##### **Correspondence:** Fadil Gradica

**Background**

Causes of ribs fracture are trauma but rib fracture can occur spontaneously due to a severe cough or sneeze. In this our study, patients with spontaneous rib fractures were analyzed according to underlying pathology, treatment, and complications.

**Methods**

Six consecutive patients are presented in our thoracic surgery service between January 2004 and December 2018 with spontaneous ribs fracture .This is retrospective study. The patients’ data were evaluated according to anamnesis, physical examination, and chest radiograpy and image CT.

**Results**

The ages of the patients ranged from 29 to 68years (mean 52. ± 10.20 years), and 4 (66%) were male. All patients had chronic severe cough and chest pain during respiration and efforts activity.. The fractures were most frequently between 6th and 12-th ribs; multiple rib fractures were detected in 1 (41.7%) patient. Four(66.7%) patients had chronic obstructive pulmonary disease, 1 (16.7%) had bronchial asthma, and 1 (16.7%) had idiopatic rib fracture. Bone densitometry were made only in two patients.. Patients with chronic obstructive pulmonary disease or bronchial asthma had been treated with high-dose steroids for over a year.

**Conclusions**

Spontaneous rib fracture due to severe cough may occur in patients withosteoporoses , chronic obstructive pulmonary disease, or bronchial asthma, receiving long-term steroid therapy. If these patients have severe chronic chest pain during the cough and efforts in the same region chest radiography should be performed to check for bone lesions.

**Key words**

Chest pain, rib fractures, spontaneous.

## P50 Early and long-term outcomes after CABG in patients with diffuse CAD

### Said Kurbanov, Elina Vlasova, Andrey Shiryaev, Vladislav Vasiliev, Damir Galyautdinov, Larisa Ilina, Garma Mayorov, Ruslan Latypov, Renat Akchurin

#### National Medical Research Center of Cardiology

##### **Correspondence:** Said Kurbanov

**Introduction**

According to research data, diffuse coronary atherosclerosis occurs in 20-40% of patients with coronary artery disease (CAD). Diffuse coronary atherosclerosis is a challenge for coronary artery bypass grafting (CABG). Despite the high level of coronary surgery, the possibilities for revascularization are currently limited. Therefore, adjunct techniques such as coronary endarterectomy (CEA) and onlay-flap anastomosis can be a solution.

**Objective**

To evaluate in-hospital and long-term clinical outcomes after CABG in patients with diffuse CAD as well as effectiveness and safety of adjunct techniques.

**Methods**

A single-center retrospective study. We enrolled 204 patients with diffuse CAD who underwent CABG (on-pump) between 2012 and 2017. Of these 177 patients who didn’t have severe comorbidity were included in this study. CABG was performed with CEA – group 1 (n=76) or with an onlay-flap anastomosis – group 2 (n=101) by using surgical microscope. We analyzed in-hospital and long-term outcomes

**Results**

Demographic and clinical characteristics were comparable in both groups. Operative and 30-day mortality were 0% in both groups. The incidence of non-fatal perioperative myocardial infarction was higher in group 1 (7,9% vs. 0%, respectively; p<0.05). During the follow-up period, data on 26 patients were lost; follow-up was therefore completed in 85.3% of patients. The median follow-up period was 45 months. Freedom from all-cause death was 93.4% in group 1 and 97.7% in group 2; freedom from cardiac death was 96.7% in group 1 and 100% in group 2. The incidence of verified myocardial ischemia recurrence was 29.9% in group 1 and 30.7% in group 2.

**Conclusion**

CABG by using surgical microscope with adjunct techniques is acceptable myocardial revascularization strategy in patients with severe diffuse CAD. CEA was associated with significant risk of perioperative myocardial infarction but did not increase operative mortality. Long-term outcomes were comparable in both groups

## P51 In-hospital outcomes after CABG in patients with coronary artery calcification

### Andrey Shiryaev, Vladislav Vasiliev, Elina Vlasova, Garma Mayorov, Said Kurbanov, Damir Galyautdinov, Larisa Ilina, Ruslan Latypov, Renat Akchurin

#### National Medical Research Center of Cardiology

##### **Correspondence:** Said Kurbanov

**Introduction**

According to the available literature data, coronary artery calcification (CAC) most often occurs in adults over the age of 70. Diffuse coronary atherosclerosis (DCA) and CAC can cause technical difficulties in coronary artery bypass grafting (CABG) and incomplete revascularization. Mostly bypass grafting associated with adjunct techniques using, such as patch-angioplasty, coronary endarterectomy and complex bypass grafts.

**Objective**

To compare in-hospital outcomes in patients with CAC of the target coronary arteries and in patients without CAC

**Materials and methods**

Patients who underwent isolated elective CABG surgery in 2017-2018 years were enrolled in the study. Based on angiographic data patients were classified in two groups: 1 – pts with calcification of the target coronary arteries (n=108) and 2 - without calcific lesion (n=354).

**Results**

Demographic and baseline clinical characteristics were comparable in both groups. Operative and 30-day mortality was 0%. The incidence of perioperative myocardial infarction was 1,8% and 1,1% in both groups respectively, all these patients were successfully discharged after recovery. Number of pts needed for inotropic support more than 24h was higher in group 1 (2,7% vs 1,7%); no significant difference. There was also no significant difference between groups for mean revascularization index (4,0±0,8 and 4,3±0,5 respectively), aortic cross-clamp time (4,0±0,8min and 4,3±0,5min respectively) and cardio-pulmonary bypass time (62±19min and 59±19min respectively). Complex bypass grafts (79,6% vs 23,7%, p<0.05), patch-angioplasty (21,3% vs 4,5%, p<0.05) and endarterectomy (16,7% vs 4,5%, p<0.05) were used more often in group 1.

**Conclusion**

CAC require higher prevalence of adjunct techniques in addition to standard approach of CABG. However, there was no evidence that CAC increased risk of in-hospital cardiac events. CABG in patients with CAC and without CAC demonstrates equal early outcomes.

## P52 10-years follow up of minimally invasive coronary revascularization

### Ruslan Latypov, Andrey Shiryaev, Vladislav Vasiliev, Damir Galyautdinov, Renat Akchurin, Said Kurbanov

#### National Medical Research Center of Cardiology

##### **Correspondence:** Said Kurbanov

Nowadays minimally invasive coronary surgery is becoming widespread in many clinics. The advantages of MIDCAB has been shown in many studies, but these results are shown on short term follow up. A discussion about the quality of anastomosis still goes on.

The aim of this study is to compare the results of MIDCAB and on-pump coronary direct revascularisation in long term follow-up.

**Materials and methods**

We included patients undergone procedures on coronary arteries in National medical research center of cardiology in period from 2007 to 2008 years. All the patients were divided into two groups: the first group includes 48 patients undergone MIDCAB and the second group includes 35 patients after on-pump CABG. We used LIMA in all cases.

**Results**

Mean age was 55 in 1 group and 59 in 2 group. We used such endpoints as general mortality, cardiac mortality, MI, angina recurrence, angioplasty with stent implantation. General mortality was the same in both groups (4,2% vs 5,7%, respectively), as well cardiac mortality (2,1% vs 2,9%, respectively). The incidence of myocardial infarction (8,3% vs 8,5%, p>0.05) and angina recurrence (39,6% vs 45,7%, p>0.05) were comparable in both groups. The results of control coronary angiography revealed the same patency rate of LIMA in both groups of patients (93% vs 95%, p>0.05).

**Conclusion**

MIDCAB procedure shows equal results vs on-pump surgery in level of total and cardiac mortality, perioperative MI and angina recurrence. LIMA patency is the same in both groups on 10-year follow up. Thus we suggest that MIDCAB procedure is safe and effective in patients with CAD on long-term follow up and can be performed in patients with LAD stenosis.

## P53 Pulmonary tuberculosis presented as spontaneous pneumothorax analyses our cases

### Fadil Gradica, L. Lish, Dh. Argjiri, A. Cani, F. Kokici, F.Gradica, V.Rexha, dr.Gulam, D.Thereska

#### University hospital ‘shefqet Ndroqi’ Thoracic surgery service and anestesie reanimacion; Public pharmacy service Tirane; Naional Center Cancer Academy hospital Beijing; University Hospital Center “Mother Theresa’ Tirane

##### **Correspondence:** Fadil Gradica

Pulmonary tuberculosis presenting as spontaneous pneumothorax is a rare but well-recognised complication. A pneumothorax implies the presence of air in the pleural space. As we know a spontaneous pneumothorax occurs without antecedent trauma to the chest wall. It may be primary or secondary. Primary spontaneous pneumothorax occurs in the absence of underlying lung disease while a secondary spontaneous pneumothorax occurs in persons with significant pulmonary disease. PTB remains an important cause of secondary spontaneous pneumothorax especially in the developing world. We present our cases with PTB presenting as secondary spontaneous pneumothorax treated in our thorax surgery service.

**Objectives**

We analyze of patients with pulmonary tuberculosis presented as spontaneous pneumothorax treated in the thoracic surgery for the period of time January 2010- March 2019.

**Materials and Methods**

During January 2010-2019 we have treated 31 patients with pulmonary tuberculosis presented as spontaneous pneumothorax .21 male and 10 female with mean age presentation 47 years (average 13-65 years old).Clinical presentation was with cough about 3 months (3days to 5 months) and breathlessness of several days’ duration and chest pain, orthopnoea, paroxysmal nocturnal dypsnoea. Cough was initially dry but quickly became productive of mucoid sputum. There was no haemoptysis or trauma to the chest wall .There was a history of fever, drenching night sweats and weight loss. Chest examination showed tachypnoea , Percussion note was hyper-resonant with loss breath sounds on the pneumothorax side .They had positive Mantoux Test and sputum was positive bacillus tuberculosis. An urgent chest radiography was requested and showed right pneumothorax in 17 patients and left side in 12 patients and hydropneumothorax (tuberculous pleural empyema et bronchopleural fistulae in 10 patiens).bilateraly spuntaneuos tubercular pneumothorax in two cases .Chest CT was performed after pleural drainage but only in five patients was performed befor chest drainage and showed the lung with reticulonodular shadows and intraparencimal cavity of the lung in one or both side .localised most comen in apex of the lung.

**Results**

All patients was inicialy treated with pleural drainage and high concentration oxygen inhalation along with anti-tubercular chemotherapy according DOTS program , daily doses of rifampicin 600mg, isoniazid 300mg, pyrazinamide 1.2g and ethambutol 800mg for 6 months. The chest tube was removed after 8 days and a post-drainage chest radiograph showed re-expansion of the lung with reticulonodular shadows. In five patients due to persistent airlake and not complete extension of the lung was treated by open surgery .Was performed decortications, aerostases, pleurectomy and adecuate pleural drainage. Mean hospital stay was 2 weeks (average 1- 6 weeks).One case operativ mortality.Comliactions was right fibrothorax two case. Following treatment they were clinically and radiologically improved, and was discharged with the advice to complete the anti-tubercular chemotherapy according DOTS –program.

**In conclusion**

PTB may present as spontaneous pneumothorax with acute severe dypsnoea and may be confused with that of the other causes of acute dypsnoea, , through history and careful examination did the diagnosis and the response to treatment is often rewarding. Morbidity and mortality from this curable disease is thus reduced.

**Keywords**

pneumothorax; pulmonary tuberculosis, pleural drainage.

## P54 Is there a necessity for medical treatment of patients with myocardial “bridges”?

### Olena Gogayeva, Liudmyla Dzakhoieva, Vasyl Lazoryshynets

#### Amosov National Institute of cardiovascular surgery NAMS of Ukraine, Kyiv

##### **Correspondence:** Olena Gogayeva

**Background**

Tunneled coronary artery (TCA) - is anomaly of location of coronary artery under muscular segment, so called myocardial bridge (MB), which leads to systolic compression of the artery. This anomaly sometimes causes myocardial infarction, severe angina pectoris, arrhythmia and even sudden death. Misunderstanding of the causes of development of symptoms is a main reason for disparaging attitude of doctors to this anomaly.

**Materials and methods**

During 13 years we observed 347 patients with symptomatic MB. ECG, ECHO, coronary angiography was performed for all patients.

**Results**

Treatment of patients is impossible without understanding of mechanisms of ischemia development. As mostly symptoms developed at the age of 30-40 years we found the key role of diastolic dysfunction in 84.1% cases, which prolong systolic compression on the first phase of diastole. There are the following methods of treatment patients with MB: drug therapy, stenting, supracoronary myotomy and CABG. For 296 patients (85.3%) we gave medical therapy, which include antiagregants, B-blockers, calcium antagonists, anxiolytics, magnesium and massive metabolic therapy. Nitroglycerin drugs may worsen symptoms and increase systolic compression. For 285 (96.3%) patients observed improvement of wellbeing, in 11 (3,7%) patients, who were resistant for therapy during 3-6 months we performed revascularization of myocardium (CABG or stenting). Also we noticed decrease of systolic compression for some patients on medical therapy after 2-3 years of drug treatment.

**Conclusions**

Method of treatment for patients determined strictly on individual basis. Medical therapy is a first step of treatment which could significantly improved of wellbeing for patients even with severe symptoms.

## P55 Neurological status of patients admitted for off-pump CABG

### Olena Gogayeva, Anatolii Rudenko, Vasyl Lasoryshynets, Liudmyla Dzakhoieva

#### Amosov National Institute of cardiovascular surgery NAMS of Ukraine, Kyiv, Ukraine

##### **Correspondence:** Olena Gogayeva

**Background**

Atherosclerosis is a systemic process, so patients admitted for CABG have not only ischemic heart disease, they also have atherosclerotic plaques of other localizations, such as carotid, limb, mesenteric arteries etc. According to the different studies- on-pump procedures have much more complications especially in neurological system.

**Material and methods**

We analyzed neurological status of 1187 patients admitted to the department for isolated CABG. For all patients we performed ECG, ECHO, coronary angiography, ultrasound of carotid arteries and CABG.

**Results**

Average age of patients was 65 y.o. Mostly all patients 1149 (96.7%) had dyscirculatory encephalopathy of varying severity, vestibulo-atactic disorders was in 569 (47.9%) cases. According to ultrasound of carotid arteries: 592 patients (50.1%) had different levels of carotid stenosis, among them: 11 patients (1.8%) had 100% occlusion of carotid artery, 217 patients (18.2%) more than 50% stenosis, 364 patients (61.4%) - less than 50% stenosis. While 80 patients (13.5%) had stroke in their anamnesis. Anesthetic and cardiological management depends on severity of carotid stenosis. It’s important to control blood pressure (BP) levels due to possible brain hypoperfusion, have to avoid BP reduction less then 125/70mmHg. 2 patients (0.8%) developed postoperative stroke, one patient (0.08%) died due to stroke. Additive Euroscore I was 4, logistic - 9.2%, while hospital mortality was 8 patients (0.6%).

**Conclusion**

Carotid stenosis screening helps to avoid neurological complications in perioperative period.

Using off-pump technic helps to minimize stroke occurrence in early postop period. Strict blood pressure control with daily injection of neuroprotectors is a way to overcome possible acute neurological events.

## P56 Advantages and disadvantages of the different thoracoscopic techniques in surgical treatment of pleural effusions

### Katerina Marinova, Rumen Nenkov, Borislav Petrov, Daniel Bulyashki

#### Thoracic surgery clinic, UMHAT “St.Marina”, Varna, Bulgaria

##### **Correspondence:** Katerina Marinova

**Introduction**

Pleural effusions are common diseases in the field of thoracic surgery. The choice and timing of intervention still remains an issue and need a discussion. In the modern era of minimally invasive video assisted surgery (VATS) these techniques must be offered as first line treatment.

**Aim**

To present and discuss the advantages and disadvantages of different thoracoscopic approaches in diagnostics and treatment of pleural effusions.

**Material and method**

In the past 6 years in our clinic have been treated with VATS techniques 122 patients with pleural effusions. 79 of them are males and 43 females in the age between 15 and 88 years. We had used Olympus video-system with 30˚ camera. The type of the surgical approach has been selected by the character of effusion - free or loculated, etiology, roentgenographic findings, patient’s habitus and the volume of surgery.

**Results**

VATS has been used in 122 patients.67 of them with inflammatory and 55 with malignant effusion - primary and metastatic. We performed triportal tehnique in 10 cases, biportal in 74 and uniportal in 38. It has been discussed the indications, advantages and disadvantages and overall benefits and outcomes of any approach, which leads to individualizing of treatment and better result for the patient.

**Conclusion**

The choice of VATS technique in the treatment of pleural effusion is important step of achieving best outcomes for every patient by using personalizing approach.

**Keywords**

Pleural effusion, video assisted thoracic surgery (VATS), Thoracic surgery, advantages and disadvantages.

## P57 Role of TGF-beta1 pathway and non-small cell lung cancer

### Zlatomir Ilinov^1^, Evelin Obretenov^1^, Maya Gulubova^2^, Mitko Mitev^3^, Julian Ananiev^2^

#### ^1^Department of Thoracic surgery, Medical Faculty, Trakia University, Stara Zagora, Bulgaria; ^2^Department of General and clinical pathology, Medical Faculty, Trakia University, Stara Zagora, Bulgaria; ^3^Department of Radiology, Medical Faculty, Trakia University, Stara Zagora, Bulgaria

##### Correspondence: Zlatomir Ilinov

**Background**

Non-small cell lung cancer (NSCLC) is still one of the most frequent and lethal neoplasm.Different prognostic biomarkers in a disease provide information regarding progression and therapy opportunities.

The aim of the present study was to evaluate the tissue expression of TGF-beta1 and TGF-beta RII and the relation with NSCLC development.

**Materials and methods**

Biopsy specimens from 43 patients with NSCLC (stage I to IIIA incl, radicaly operated) were examined for the presence of TGF-beta1 and TGF-beta RII by immunohistochemistry. The correlation between expression of the markers and patient clinicopathological parameters was evaluated.

**Results**

After analysis we found that 41.9% had high cytoplasmic TGF-beta1 expression and 30.2% was expressed on tumor cell membranesreceptor-TGF-beta RII. The protein and its receptor correlated positively in 33.7% of cases (χ2=4.09; p=0.025).In addition we found that20.9% of TGF-beta1 positive cases had lymph node metastasis (χ2=3.56; p=0.031). Also, compared TGF-beta1/TGF-beta RII expression and clinical stage distribution showed tendency.

**Conclusions**

This results suggest that TGF-beta1 and his receptor –RII may play an important role in development and progression of NSCLC.

